# Identification and Comparative Profiling of miRNAs in an Early Flowering Mutant of Trifoliate Orange and Its Wild Type by Genome-Wide Deep Sequencing

**DOI:** 10.1371/journal.pone.0043760

**Published:** 2012-08-28

**Authors:** Lei-Ming Sun, Xiao-Yan Ai, Wen-Yang Li, Wen-Wu Guo, Xiu-Xin Deng, Chun-Gen Hu, Jin-Zhi Zhang

**Affiliations:** Key Laboratory of Horticultural Plant Biology (Ministry of Education), College of Horticulture and Forestry Science, Huazhong Agricultural University, Wuhan, China; National Taiwan University, Taiwan

## Abstract

MicroRNAs (miRNAs) are a new class of small, endogenous RNAs that play a regulatory role in various biological and metabolic processes by negatively affecting gene expression at the post-transcriptional level. While the number of known *Arabidopsis* and rice miRNAs is continuously increasing, information regarding miRNAs from woody plants such as citrus remains limited. Solexa sequencing was performed at different developmental stages on both an early flowering mutant of trifoliate orange (precocious trifoliate orange, *Poncirus trifoliata* L. Raf.) and its wild-type in this study, resulting in the obtainment of 141 known miRNAs belonging to 99 families and 75 novel miRNAs in four libraries. A total of 317 potential target genes were predicted based on the 51 novel miRNAs families, GO and KEGG annotation revealed that high ranked miRNA-target genes are those implicated in diverse cellular processes in plants, including development, transcription, protein degradation and cross adaptation. To characterize those miRNAs expressed at the juvenile and adult development stages of the mutant and its wild-type, further analysis on the expression profiles of several miRNAs through real-time PCR was performed. The results revealed that most miRNAs were down-regulated at adult stage compared with juvenile stage for both the mutant and its wild-type. These results indicate that both conserved and novel miRNAs may play important roles in citrus growth and development, stress responses and other physiological processes.

## Introduction

Posttranscriptional and translational regulation mechanisms mediated by small RNAs are endemic in both animals and plants. Based on the difference of biogenesis and action, small RNAs can be divided into microRNAs (miRNAs) and small interfering RNAs (siRNAs) at a broad level [1]. MiRNAs were small, non-coding RNAs generated from single-stranded precursors with hairpin structures, while siRNAs were generated from double-strand RNA precursors [2]. Previous studies showed that miRNAs play pivotal roles in regulating diverse plant developmental processes by targeting mRNAs for translational repression, cleavage, or destabilization [3], including leaf morphogenesis [4], boundary formation/organ separation [5], root development [6], organ polarity [7], flower development [8], phase changes from vegetative growth to generative growth [8] and flowering [9]. Since the first identification of *lin4* in *Caenorhabditis elegans* [10], a total of 21,643 miRNAs were identified from 168 species. More recently, a growing number of new miRNAs in plants have also been identified. To date, more than 5500 genes encoding miRNAs have been annotated in *Arabidopsis*, rice and other plant species, belonging to 250 miRNA families (http://www.mirbase.org/, miRBase Release 18.0).

Identification of comprehensive sets of miRNAs and other small RNAs in different plant species was a critical step to facilitate our understanding of regulatory mechanisms or networks for target genes [11]. Three major approaches have been used for miRNA discovery in plants: forward genetics, computational prediction of conserved miRNAs by searching for homologous sequences using ESTs and genomic sequences as well as traditional Sanger sequencing of the small RNA library [12]. Only a few miRNAs have been identified by forward genetic studies [5], the computation-based approach is mostly limited to the discovery of conserved miRNAs and was difficult to predict species-specific miRNAs; the small-scale traditional sequencing approach identifies mainly conserved miRNA, because newly evolved and species-specific miRNAs were often accumulated at a lower level and often missing from the datasets derived [13]. The recently developed deep sequencing methods provided a rapid way to identify and profile small RNA populations in different plants, mutants, tissues, and at different stages of development [12]. This strategy has been successfully applied to both model plants [14], [15] and non-model plants [12], [16], and revealed that some miRNA families were well-conserved across plant species, suggesting that the regulatory role of many miRNAs may be universal in plants. Using the deep sequencing method revealed many species-specific miRNAs and provided the genomic landscape of miRNAs.

MiRNAs were first identified as regulators of the juvenile-to-adult transition in *Caenorhabditis elegans* [10]. Recent studies indicated that miRNAs and other endogenous miRNAs also regulated developmental transitions in plants. Several miRNAs, such as miR156 and miR172, have been shown to affect flowering time when over-expressed in *Arabidopsis* [8], [17]. Over-expression of miR156 prolongs the expression of juvenile vegetative traits and delays flowering in both *Arabidopsis* and maize [18], whereas over-expression of miR172 in *Arabidopsis* accelerates flowering [8]. MiR172 exhibits a similar temporal expression pattern in maize, where it targets *Glossy15* (*Gl15*), a gene required for the expression of juvenile epidermal traits [19]. However, woody plants and many annual plants show characteristic variation as they progress from a juvenile to a mature stage, a process known as phase change. Annual plants complete their life cycle in one year and initiate flowering only once, whereas most woody plants including most fruit crops have a long juvenile period, during which no reproductive development occurs. How perennials woody plants undergo a long silence juvenile stage followed by repeated vegetative growth and flowering has been extensively studied in transcriptional study. However, whether or how miRNAs repress woody plants flowering before transition has not been elucidated in post-transcriptional and translational regulator.

Citrus is one of the most important fruit crops in the world. Citrus fruit is produced commercially for fresh fruit, juice, raisins, and transformed into high value-added products such as wines and spirits [20]. Flowering is an essential stage for fruit production, and thus it is important to understand the genetic mechanisms underlying the flowering event for genetic improvement. In 1976, a spontaneous mutant derived from *Poncirus trifoliata* (L.) Raf with short juvenile phase, namely, precocious trifoliate orange (MT), was found in Yichang, Hubei province, China. Compared with 6 to 8 years of the wild-type trifoliate orange (WT), almost all of the seedlings germinated from the MT only have 1 to 2 years’ juvenile period, and 20% seedlings even flowered in the year after germination. The MT seedlings can flower 2–3 times per year while the WT only once per year [21]. So the MT was speculated to be a direct variant of the WT, which is an ideal material for studying floral induction and flowering molecular mechanism. Thereafter, transcriptional study including cDNA macroarray in combination with suppression subtraction hybridization was used to investigate gene expression changes in the MT, and a total of 368 differentially expressed genes were detected [22]. Meanwhile, comparative proteomic analyses were performed between the MT and its WT. And the proteomic data, when considered in combination with transcriptional data, suggest that post-transcriptional regulations are involved in shaping the early flowering trait of the MT (Zhang et al., unpublished data). However, it remains largely unknown what kind of post-transcriptional mechanism was involved in the MT.

The study of miRNAs in citrus has been reported [16], [23]. However, in comparison with other woody plants such as *Vitis vinifera* and *populus*, the number of citrus miRNAs identified so far is still quite small. The deep sequencing study is well suited for the discovery of species-specific miRNAs expressed at low abundance. Therefore, we describe here Solexa sequencing and analysis of small RNA transcriptome from the MT and its WT. Several million miRNAs were produced from four libraries for two genotypes. A total of 216 miRNAs, known and novel, were identified based on either sequence homology or the secondary structure of their precursors. The annotation of the potential targets of differential miRNAs indicated that highly ranked genes those that are required in biological processes. Our studies not only significantly increase the number of miRNAs in citrus, but also provide useful information for miRNAs that regulate citrus growth and development processes.

## Materials and Methods

### Plant Materials

Precocious trifoliate orange and its wild-type samples were collected in the experiment fields of the National Citrus Breeding Center at Huazhong Agricultural University, Wuhan, China (30°28′ N, 114°21′ E, 30). The seeds of the MT and its WT were planted in 20-cm pots containing a potting mix of a commercial medium (PeiLei, China) and perlite at a ratio of 3∶1 for 2 months in a greenhouse at 25°C with 16 h light/8 h dark. The juvenile potted seedlings were then transplanted and grown under field conditions. These juvenile trees were watered regularly with nutrient solution. Shoot meristems of the juvenile MT trees and its WT were collected in June. Lateral buds of spring shoot (in citrus, there are three flushes during the growing season, and the spring flush is the most important one for growth and flower formation, these buds develop to a spring shoot in the next year) of the MT and its WT trees were collected after self-pruning in April next year (the shoot tip has just started to fall, which occurs in mid-April). In addition, to analyze the fluctuation of miRNA expression and its relationship to flower development, the spring shoots at three distinct phases (15 days before self-pruning, during self-pruning, and 20 days after self-pruning) were collected from two-year-old trees of both the MT and its WT. All materials were collected from three individual plants and immediately frozen in liquid nitrogen and stored at –80°C until analyzed.

### Small RNA Library Construction and Sequencing

Total RNA was isolated using Trizol (Invitrogen, USA). A 1.2% agarose gel, stained by ethidium bromide, was run to preliminarily indicate the integrity of the RNA. All RNA samples were quantified and examined for protein contamination (A_260/280_) and reagent contamination (A_260/230_) by a Nanodrop ND 1000 spectrophotometer. In addition, the RIN (RNA integrity number) determined by the Agilent Technologies 2100 Bioanalyzer was greater than 8.5 for all samples.

Small RNA library construction and deep sequencing was as described previously [24]. Briefly, after PAGE purification of small RNA molecules (18–30 bases) and ligation of a pair of Solexa adaptors to their 5' and 3' ends, the small RNA molecules were amplified using the adaptor primers for 17 cycles and fragments of around 90 bp (small RNA + adaptors) were isolated from agarose gels. The purified DNA was used directly for cluster generation and sequencing analysis using the Illumina Genome Analyzer (Illumina, San Diego, CA, USA) according to the manufacturer's instructions. The image files generated by the sequencer were then processed to produce digital-quality data. After masking of adaptor sequences and removal of contaminated reads, clean reads were processed for computational analysis.

### Discovery of Conserved and Novel miRNA Families

Low quality sequence reads were removed according to the criteria of Solexa, and identical sequence reads were tabulated to produce a ‘read count’ score. Duplicated sequences were eliminated from the initial dataset to produce a non-redundant set of unique sequences, hereafter referred to as sequence reads. After trimming the ligated adaptor sequences, identical sequences were counted as their expression abundances. We clustered the remaining reads based on sequence similarity and the dominant reads were analyzed as follows: the reads were analyzed by BLAST against citrus genome (http://www.phytozome.net/clementine.php) to discard rRNA, tRNA and snRNA. Subsequently, the remaining sequences were analyzed by BLAST search against miRBase (http://www.mirbase.org/, miRBase Release 18.0). Sequences in our libraries with identical or with three or fewer mismatches to currently known miRNAs from other plant species were regarded as potential conserved miRNAs. The potential miRNA sequences were used for BLAST search against citrus genome dataset; the perfectly matched sequences were used for fold-back structure prediction by the Mfold program.

To identify novel miRNAs, the MIREAP algorithm was employed to obtain all candidate precursors with hairpin-like structures that were perfectly mapped by sequencing reads (http://sourceforge.net/projects/mireap). Briefly, the putative miRNA precursor sequences were folded using RNAfold [25]. The essential criteria [26] were used for selecting the miRNA candidates, e.g. sequences of miRNA precursors can fold into a hairpin secondary structure that contains the ∼21 nt mature miRNA sequence from one arm and miRNA* derived from the opposite arm, both of which form a duplex with two nucleotides, 3' overhangs.

### Prediction of Potential Targets of miRNAs

The potential targets of miRNAs were predicted using the psRNATarget program (http://bioinfo3.noble.org/psRNATarget/) with default parameters. The rules used for target prediction are based on those suggested [27], [28], with penalty scores ≤ 2 for mismatched patterns in the miRNA/mRNA duplexes. In addition, one mismatch was allowed in the region complementary to nucleotides 2–12 of the miRNA, but not at the cleavage site (nucleotides 10 and 11), and three additional mismatches were permitted between nucleotide positions 12 and 21, but no more than two consecutive mismatches within this region. The number of allowed mismatches at complementary sites between miRNA sequences and potential mRNA targets was no more than four, and no gaps were allowed at the complementary sites. Putative target genes were manually selected from these candidates based on their localization in citrus genome (http://www.phytozome.net/clementine.php). Functions of the predicted target genes were assigned manually according to the functions of the best hits from the BLAST search against the NCBI database. The hybridization energy of each miRNA-predicted target duplex (△G) was determined from the WMD3 output.

### Functional Annotations of the Potential Targets of miRNAs

To investigate biological processes possibly regulated by miRNAs, annotations of putative functions to potential target genes of miRNAs were performed using the program annot8r, which was run locally to BLAST against a reference database that stores UniProt entries, their associated Gene Ontology (GO), Enzyme Commission (EC) and Kyoto Encyclopaedia of Genes and Genomes (KEGG) annotations [29]. The GO categorization results were expressed as three independent hierarchies for biological process, cellular component, and molecular function [30].

### Stem-loop Quantitative RT-PCR Assay

Assays to quantify the mature miRNAs were conducted as described previously [31]. Briefly, 3 µg of total RNA was reverse-transcribed to cDNA using SuperScript III Reverse Transcriptase (Invitrogen, USA) by a pulse reverse transcription program and looped antisense primers. The mix was incubated at 65°C for 5 minutes, 37°C for 60 minutes, and 70°C for 15 minutes. This allowed for the creation of a library of multiple miRNA cDNAs. Stem-loop primers for reverse transcription and primers for quantitative PCR were list in Supporting Information S1. The electrophoresis of the PCR products was performed on 3% agarose gel for 40 min under 115V voltage. Real-time PCR was performed on the LightCycler™ 480 System (Roche Applied Science, Mannheim, Germany) using *β-actin* as endogenous control. Briefly, the primers for miRNAs and *β-actin* were diluted in the SYBER GREEN PCR Master Mix (PE Applied Biosystems) and 20 µl of the reaction mix was added to each well. Reactions were performed by an initial incubation at 50°C for 2 min and at 95°C for 1 min, and then cycled at 95°C for 15 s and 60°C for 1 min for 40 cycles. Data were evaluated by calibrator-normalized relative quantification with efficiency correction using the RelQuant software version 1.01 or the LightCycler™ 480 software version 1.5 (Roche Applied Science, Mannheim, Germany) and normalized to expression of *β-actin*. Real-time quantitative PCR was performed in four replicates for each sample, and data were indicated as means ± SD (*n*  =  4).

### Regional Amplification Quantitative RT-PCR (RA-PCR)

A signature of an mRNA target site cleaved by a miRNA-guided process is a decrease in the RT-PCR amplification of a region flanking the target site [32]. After miRNA-induced cleavage, only the 3′ fragment of the target mRNA possesses a poly (A) tail. When poly (T) adapters are used to prime reverse transcription, only the 3′ end of the cleaved mRNA should be copied into cDNA. Intact mRNA molecules will be copied in their entirety. Previous studies of mRNAs with miRNA target sites suggested that the 5′ region of RNA (upstream) of the cleavage site was less stable than the 3′ region (downstream) of the cleavage site [33]–[35]. One can generally expect the RT-PCR product from the upstream region to be present at levels no greater than the downstream region because reverse transcription with poly (T) adapters would not generate cDNA beyond a cleaved site. Thus, a method we refer to as regional amplification quantitative RT-PCR was developed to monitor the miRNA directed cleavage of mRNAs [34]. For each potential mRNA target, three sets of primers were designed to amplify three different regions: a 5′ region and a 3′ region, and a middle region. The middle region includes the target site. The reverse transcription of the miRNA-cleaved mRNA will not generate a cDNA beyond the cleaved site. The same protocols described above for the quantitative RT-PCR of miRNA were used for RA-PCR.

## Results

### Sequencing the Small RNA Library Using Solexa Sequencing

**Figure 1:**
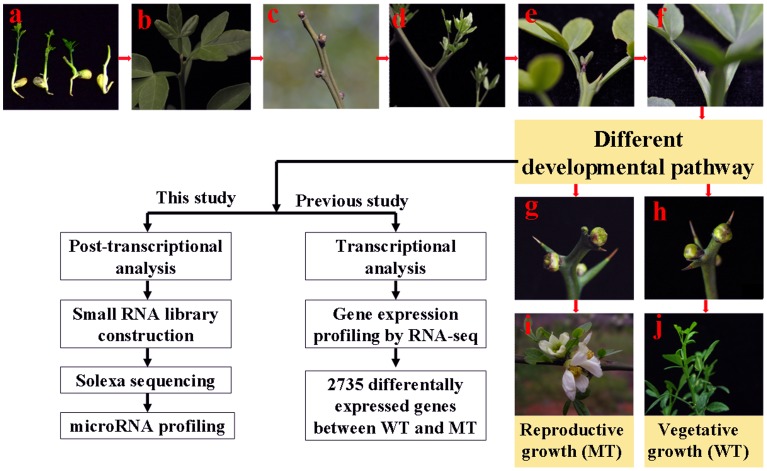
Schematic diagram of developmental stages involved in the flowering of precocious trifoliate orange. a, Seedling of precocious trifoliate orange. b, The shoot apical meristem of seedling begins self-pruning. c, The terminal bud and lateral buds of the juvenile trees became dormant after self-pruning. d, The lateral buds develop into a spring shoot in the next year. e, The spring shoot begins self-pruning. f, After self-pruning. g, The lateral bud becomes a leaf bud. h, The lateral bud becomes a floral bud. i, floral bud became flower. j, leaf bud became summer or autumn. The schematic presentation shows the strategy of transcriptional analysis by the Massive Parallel Signature Sequencing (previously) and post-transcriptional analysis by sRNA sequencing (this study) on the MT and WT.

Previous studies have demonstrated that the flowering development of precocious trifoliate orange was a highly complex and integrated set of developmental processes coordinated by the action of hundreds of genes [36]. To understand whether miRNA was involved in the process, the miRNA levels were investigated in juvenile and adult both the MT and its WT by Solexa sequencing. A major characteristic of the MT was that its juvenile phase was shortened to 1 to 2 years (Figure 1), whereas the WT has a long juvenile period of 6 to 8 years. On the other hand, our previous studies showed that the stage of self-pruning on spring shoots (April next year) was the critical stage for the MT; cytological observation revealed that the floral buds in the MT initiated differentiation immediately after self-pruning on spring shoots (Figure 1). For the WT, the spring shoots, which did not form floral buds, began to produce vegetative buds after self-pruning (Figure 1). So four independent RNA samples from the MT and the WT (June and April next year) were prepared in this study, four RNA libraries were generated and sequenced by Solexa sequencing (Solexa sequencing data have been deposited into the NCBI/GEO database with accession number GSE37115).

**Figure 2:**
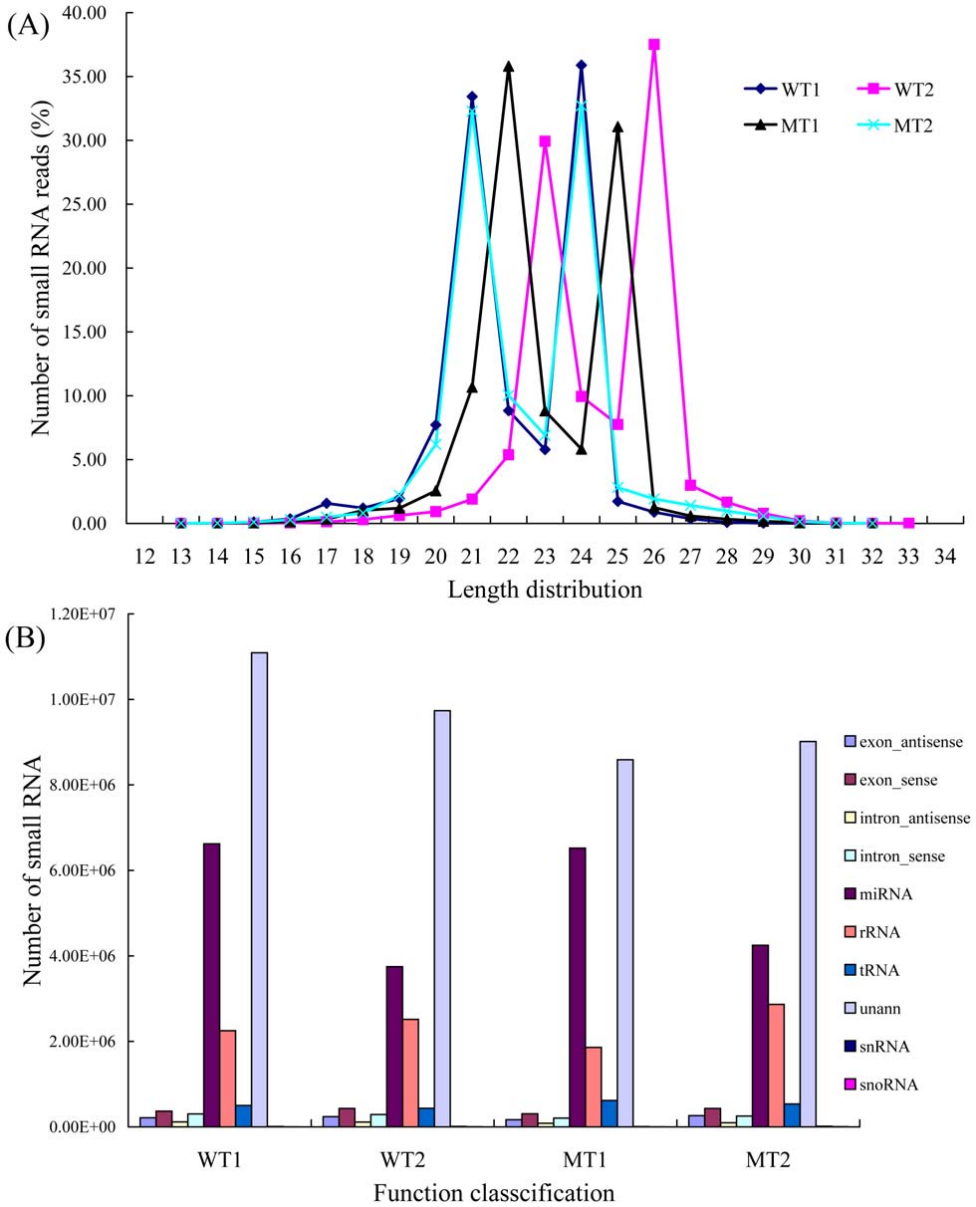
Length size distribution of small RNA. The length size distribution (A) and proportions of various categories (B) of small RNAs in the MT and its WT at different developmental stages.

More than 36 million original sequencing reads were produced with approximately 9–10 million raw reads from each library. After discarding low quality, filtering 5′ contaminant and trimming 3' adaptor reads, a total of 21,478,087 and 17,526,615, 18,364,794 and 17,736,041 clean reads were obtained from stage 1 of the WT (June, WT1), stage 2 of the WT (April next year, WT2), stage 1 of the MT (June, MT1), stage 2 of the MT (April next year, MT2), respectively. Since most of the small RNAs with known functions are 20–24 nt long, as shown in Figure 2A, the majority of small RNAs were 20–24 nt in four libraries, which is within the typical size range for Dicer-derived products and in agreement with most of the previous results [12], [13], [16]. Although the total numbers of reads in four RNA libraries were similar, the size distribution of reads was substantially different (Figure 2A). For example, 6,737,837 (35.81% of clean reads from the MT1 library) sequences were canonical 21 nt small RNAs with the most abundant small RNAs. While 5,726,967, 7,180,124 and 5,245,715 reads of 21 nt were in the other three libraries, accounting for 32.29% of clean reads from the MT2 library, 33.43% of clean reads from the WT1 library and 29.93% of clean reads from the WT2 library, respectively. Among these sequences, the number of 24 nt sequences was significantly greater than shorter or longer sequences in the WT2 library, the 24 nt reads showed the highest redundancies (37.51%). The 24 nt reads constitute 35.89% and 32.72% in WT1 and MT2 library, while they only account for 31.07% in the MT1 library. This suggested an expansion of miRNA families in the citrus, and a diversification of miRNA producing pathways.

It was essential to generate a reference set of annotations for exploring the small RNA categories. All identical Solexa reads in each library were sorted into unique sequence reads for further analysis. When aligned, all sequences were read against the citrus genome (http://www.phytozome.net/clementine.php) using SOAP2 [37], about 60% of reads matched perfectly and 40% were from un-annotated genome sites with one mismatch in four libraries. For instance, 11,807,452 (64.29%) clean reads that grouped into 1,044,117 unique reads were matched to the citrus genome in the MT1 library. Subsequently, for each library approximately 50% of clean small RNAs were identified as products processed from rRNAs, tRNAs, snRNAs, or other non-coding RNAs (Figure 2B). Another fraction (approximately 50%) was predominantly derived from un-annotated or repeated sequences. Large portions of annotated small RNAs were mainly non-coding RNAs. For the MT1 library, 6,520,783 clean sequences which were classified into 25,882 unique reads were considered to be potential miRNAs, 4,248,573 (33,598 unique reads, MT2 library), 6,621,559 (30,052 unique reads, WT1 library) and 3,748,679 (326,112 unique reads, WT2 library) clean sequences were considered to be potential miRNAs in the remaining three libraries, respectively. Notably, about 6 million reads were miRNA candidates at stage 1 (June), accounting for nearly 1.5 times (4 million reads) miRNAs of the stage 2 (April next year) in the two genotypes. It is estimated that miRNAs might be the most abundant class of small RNAs regulated at post-transcriptional levels during plant the transition from vegetative to reproductive development.

### Identification of Conserved Candidate miRNAs

To identify conserved miRNAs from two genotypes of trifoliate orange at different development stages, small RNA sequences were compared with miRBase 18.0. After a sequence similarity search, 78 miRNAs matched known miRNAs in citrus or other species (54 known miRNAs corresponding to 36 miRNA families in *Citrus sinensis* and 24 known miRNAs corresponding to 15 miRNA families in other species, Supporting Information S2). In our previous study, a total of 155 trifoliate orange-specific miRNAs were identified in precocious trifoliate orange [38]. In this study, we observed that 63 (41%) out of 155 miRNAs were detected in four libraries by deep sequencing (Supporting Information S2). In addition, a total of 24 additional sequences were highly homologous to miRBase-recorded plant miRNAs had hairpin-shaped precursors and were selected as miRNAs (Supporting Information S3). Among 54 known citrus miRNAs, four conserved miRNA*s (csi-miR482a*, csi-miR172a*, csi-miR166e*, and csi-miR162*) were also sequenced. The sequencing results showed that the expression abundance of csi-miR482* was extremely higher than that of miR408 in all four samples, the abundance of csi-miR482* ranged from 2,167 to 11,029 reads at different developmental stages. However, csi-miR172a*, csi-miR166e*, and csi-miR162* sequence expressions were rather low. Interestingly, the abundance of csi-miR482* was higher in the MT than in the WT (Supporting Information S2).

The sequencing frequencies for miRNAs in our four libraries were used as an index for estimating the relative abundance of 141 known miRNAs. The distribution patterns of miRNA frequencies varied greatly, indicating that these miRNAs were expressed ubiquitously in each library. For 78 conserved miRNAs, four miRNA family reads (miR156, miR166, miR157, and miR167) occupied 79.47% of expressed miRNA reads on average (Supporting Information S2). Several miRNAs families such as miR168, miR160 and miR535 had moderate abundance of expression. The sequencing frequency of the four most abundantly expressed miRNAs constituted 94% of the total trifoliate orange-miRNA sequencing reads, suggesting that they might be ubiquitously expressed in trifoliate orange. In contrast, some miRNA families showed very low abundance of expression in four libraries, with several read counts only. For 63 known trifoliate orange-specific miRNAs, the read number was much lower than conserved miRNAs except PtmiR59 and PtmiR165 (Supporting Information S2). It is possible that these miRNAs are expressed at very low levels, in limited cell types, and/or under limited circumstances.

### Analysis of Novel miRNA Candidates

We used the characteristic hairpin structure of miRNA precursors to predict novel miRNAs [39]. We identified 75 unique miRNA sequences with miRNA* as potential novel miRNAs based on recent criteria for miRNA annotation [26], their pre-miRNAs, secondary structures and chromosomal locations were listed in Table 1. The length of the predicted novel miRNA precursors varied from 74 to 343 bp, with an average of 132 bp. The average minimum free energy (MFE) value was −52.41 kcal/mol, with a range of −18 to −140.2 kcal/mol, this was similar to the computational values of *Arabidopsis* miRNA precursors (−57 kcal/mol) and much lower than folding free energies of tRNA (−27.5 kcal/mol) or rRNA (−33 kcal/mol) [40]. The structures of 75 novel miRNA precursors were shown in Supporting Information S4. Meanwhile, 75 miRNA-star sequences (miRNA*), which provided convincing proof for the presence of DCL1-processed stem-loops, which were characteristic of bona fide miRNAs. Only one member was identified in each novel miRNA family and the read number for each novel miRNA was much lower than conserved miRNAs except Novel56 (Table 1). The novel miRNA size distribution ranged from 20 to 24 nt, with 21 nt the most abundant in sequencing frequency (46.6%). Analysis of the nucleotides at the ends of these miRNAs revealed that uridine (U) was the most common nucleotide at the 5' end (54.4%). To investigate whether these novel miRNA sequences were conserved across plant species, we used them as query sequences to perform Blastn searches against the *Malus domestica*, *Eucalyptus grandis*, *Populus trichocarpa*, *Prunus persica* and *Vitis vinifera* genome databases in Phytozome v8.0 (http://www.phytozome.net/). No perfect matches were found, suggesting that these newly identified miRNA sequences were not broadly conserved within woody plants.

**Table 1:** Potential novel miRNAs from the MT and its WT by Solexa sequencing.

miRNA	Location in genome	MFE	5′ arm (5′-3′)	3′ arm (5′-3′)	WT1	WT2	MT1	MT2
Novel01	scaffold_109:95168:95314	−41.2	TCTTGCTCAAATGAGTATTCCA	AGATACTCATTTGAGCTAGAAG	22	0	19	0
Novel02	scaffold_10:2672216:2672314	−53.01	CCATACCACAGCTGGATTCAGCC	CTGGATTCAGCTGTGGTATGGTA	0	14	7	0
Novel03	scaffold_10:4971000:4971152	−61.94	TAGCCAAGGATGACTTGCCT	GCAAGTCGTCTTTGGCTAGTC	0	0	74	0
Novel04	scaffold_15:2215354:2215608	−91.75	TAACGTTGACCAGTTGCACTAGT	ATGTGCAATAGGTCAATGTTAGG	0	0	6	0
Novel05	scaffold_19:1984361:1984493	−63.1	GGGCAATTCTCCTTTGGCAGA	CGCCAAAGGAGAATTGCCCTG	0	0	12	0
Novel06	scaffold_1:13086671:13086815	−57.94	TGAGGAAATTTCTGGAATGGG	CATTCCTTGGATTTCCTGCATT	0	0	23	0
Novel07	scaffold_22:3381361:3381439	−34.2	TCCTTGGGGTGATCTCGTAGT	GATGGGGTCGGTCCATGGATT	0	0	13	0
Novel08	scaffold_27:847685:847792	−72.1	GTGCCACAGTTGCATCCAGTC	CTGGATGTAATTGTGGCACGG	0	122	130	0
Novel09	scaffold_27:1650451:1650560	−73.1	GCCCCAATCCGTGGACAAAGG	TTTGTCCACGGATTGGGGCCA	0	0	65	0
Novel10	scaffold_2:3455812:3455930	−44.1	AGCTTGAGTCTTGCTGAAAGTA	CTTTCAGCAGCCTCCGGCGTC	0	51	46	0
Novel11	scaffold_2:6255278:6255381	−45	TTCTTTTTGCTACTTCTACTG	GTGGAAGTAGCAAAGAAAAGC	0	0	10	0
Novel12	scaffold_64:1246869:1246958	−35.6	TTTTGTTGCATGATGCTGATAA	ACCCGCATCATGCAACAAAAG	0	26	47	0
Novel13	scaffold_73:771564:771820	−80.02	GCAAGTCGTCTTTGGCTATTT	TAGCCAAGAATGACTTGCCCG	202	0	96	0
Novel14	scaffold_73:771772:771902	−54.6	TAGCCAAGGATGACTTGCCT	GCAAGTCGTCTTTGGCTATT	231	0	104	0
Novel15	scaffold_82:519444:519576	−70.3	TGCAACTGTGGTACGGTACCA	GTACCATACCACAGTTGCAAC	0	0	26	116
Novel16	scaffold_83:437489:437629	−56.7	TAGCCAAGGATGACTTGCCT	AGCAAGCATCCTGGGCTAAT	239	39	210	53
Novel17	scaffold_8:587742:587859	−65.9	GTTGAACGTAATATACACACA	TGTGTATGTTACGTTCAACGT	0	26	47	0
Novel18	scaffold_95:631652:631790	−57.4	TAGCCAAGGATGACTTGCCT	AGGCAGTCTCCTTGGCTAAG	122	8	71	0
Novel19	scaffold_95:743113:743226	−51.21	TAGCCAAGGATGACTTGCCT	GCAGTCTCCTTGGCTAACT	103	0	39	0
Novel20	scaffold_118:430222:430304	−21.6	ATGGAGTAAATCATGGCCGTCGG	GGACGCTCAGGATTGCGCCATGT	0	0	0	21
Novel21	scaffold_128:225135:225278	−89.7	TATGTTGCAACTGTGGTATGGTA	CCATACCACAGTTGCAACATAGC	328	184	470	210
Novel22	scaffold_129:473882:473988	−28.4	ATTGACTGATGAGGTGTCACAAT	TGTGGCAGCATATCAGTGGACGA	0	0	0	23
Novel23	scaffold_12:4547645:4547798	−92.1	TTCGGGATTTTAAAGTGCGGG	CGCACTTTAAAATCCCGGATT	0	162	0	151
Novel24	scaffold_14:4093405:4093478	−20.6	GTGGATTGGATGCGGATTTGA	ATATCCAACCCATCATTTTACAG	0	0	0	9
Novel25	scaffold_15:3899911:3900025	−55	TGCCTGGCTCCCTGTATGCCG	GCGTACGAGGAGCCAAGCATA	9456	14747	7725	10985
Novel26	scaffold_16:1864426:1864631	−140.2	TCATGCAATTGTAGTCAAAGT	TTTGACTACAATTGCATGACA	0	14	0	10
Novel27	scaffold_18:88175:88313	−49.3	GGGACTGTTGTCTGGTTCAAGG	TCGGACCAGGCTTCATTCCCCT	0	0	0	163
Novel28	scaffold_1:4611433:4611594	−59.94	TCGCTTGGTGCAGGTCGGGAA	CCCGCCTTGCATCAACTGAAT	34741	58284	18691	38895
Novel29	scaffold_27:1236405:1236538	−46.7	TTGGACAGAGAAATCACGGTCA	ACCGTGTTTCTCTGCCCAATC	427138	300236	399942	423804
Novel30	scaffold_2:4942371:4942595	−100.8	AAGCCAAAATGACACCCTTTCCT	GAAAGAGTGTCGTTTTTGGCTTGG	0	0	0	6
Novel31	scaffold_30:2196438:2196578	−34.6	AGAGAAGTAAGATATTTCCTTGG	AAGGATGTATCTTTTGTCTTTGA	0	0	0	7
Novel32	scaffold_39:1391080:1391195	−57	CGCACCCCAGCGTGGAACCATC	TGGTGCCACGCTGTGTGCGTC	122	8	86	53
Novel33	scaffold_39:1799757:1799942	−70.5	CAGCCAAGGATGACTTGCCGG	CGGCAAGTTGTCTTTGGCTAC	757	185	712	164
Novel34	scaffold_3:6400070:6400194	−46.14	TGAAGCTGCCAGCATGATCTA	GGTCATGCTCTGACAGCCTCACT	0	5811	3490	9998
Novel35	scaffold_49:246191:246294	−42	TGACAATGAGAGAGAGCACAC	GTGCTCTCTACCATTGTCATA	180	73	80	85
Novel36	scaffold_51:957676:957782	−76.2	TTGTGCTGAGCACCGGATCAA	GATCCGGTGCTCAGCACAAGC	0	16	0	37
Novel37	scaffold_52:447370:447483	−37.6	AACAAGCGTGTAGATCAGCTG	GCTGCGTTAAATACTTGTTGG	0	0	0	24
Novel38	scaffold_53:1195181:1195275	−52.9	TTGTCGCAGGAGCGGTGGCACC	TGCCAGCATCCTGTGACAAGA	159	0	0	422
Novel39	scaffold_57:106595:106727	−87.6	TGCAACTGTGGTACCGTGCTA	GCACGGTACCACAGTTGCACC	43	0	42	68
Novel40	scaffold_5:715526:715624	−41.1	TGTTGGCATAGCTCAATCCGA	TGATTGAGCCGTGCCAATATC	0	0	0	68
Novel41	scaffold_6:4465828:4465981	−59.5	TTCCAAAGGGATCGCATTGATC	TCATGCGATCCCTTCGGAATT	374	1411	260	1834
Novel42	scaffold_77:492511:492620	−23.52	TGGAAAATAATGATCGTAGAA	CTACGCTCTACTGTTTGCCACCAAT	3192	616	2477	7
Novel43	scaffold_81:895369:895480	−84	TCATACCACAGCTGCACCTAG	AGGTGCAGCTGTGGTATGGTA	0	0	0	31
Novel44	scaffold_8:1444329:1444467	−56.5	GCGGCATCATCAAGATTCACA	GAATCTTGATGATGCTGCAT	0	0	26	116
Novel45	scaffold_8:3530405:3530512	−47.7	GTAGCATCATCAAGATTCACA	AGAATCTTGATGATGCTGCAT	9464	27362	8865	32423
Novel46	scaffold_9:4701627:4701969	−107.6	GGAATGTTGTCTGGCTCAAGG	TTGGACCAGGCTTCATTCCAC	3593	0	0	1931
Novel47	scaffold_11:564547:564628	−38	TTTAAAATCACACGGCTTTAA	AAAACCATGCGGTTTTAAAGG	7	0	0	0
Novel48	scaffold_11:2291314:2291391	−18.7	CAATCGATGGATTGGATATGGA	CGTAAAAATCCGTGAATCTGTA	18	0	0	0
Novel49	scaffold_164:51980:52061	−22.2	ACCCAAATCACACAATCAAAGTTT	ATTTATTGTTGATATGGGTCA	30	0	0	0
Novel50	scaffold_20:1575648:1575778	−58.9	CCACAGGGGCGACCTGAGAAC	TCTCATGTCGCCCCTGCGGGA	6	0	0	0
Novel51	scaffold_3:4619018:4619126	−42.79	TGGAGAAGCAGGGCACGTGCAAA	TGCACGCGCTCCCCTTCTCCAAC	23	15	25	0
Novel52	scaffold_4:4045480:4045585	−40.6	TCCCACAGCTTTATTGAACCGC	AGTTCAAGAAAGCTGTGGAAAA	11	0	0	0
Novel53	scaffold_4:4081925:4082032	−38.9	CGATATTGGTGAGGTTCAATC	TTGAGCCGCGCCAATATCACT	53	0	0	0
Novel54	scaffold_4:4121669:4121798	−60.7	GTGACAGAAGAGAGTGAGCAC	GCTCACTCTCTATCTGTCACC	47	16	0	0
Novel55	scaffold_76:1019946:1020071	−23.67	ATAGTCTGAAGTAGAAGATAGTT	CTAGCATTTTGACTTCAGATCGTGTTA	10	0	0	0
Novel56	scaffold_80:25066:25172	−49.8	TTGACGGAAGATAGAGAGCAC	GCTCTCTATTCTTCTGTCATCA	3993830	1867066	4155155	2288840
Novel57	scaffold_95:696868:696981	−44.9	TAGCCAAGGATGACTTGCCT	GCAGTCTCCTTGGCTAAGC	150	8	86	0
Novel58	scaffold_95:743113:743226	−51.21	TAGCCAAGGATGACTTGCCTGA	AGGCAGTCTCCTTGGCTAACT	103	0	39	0
Novel59	scaffold_9:4621734:4621925	−59.1	TTGGACCAGGCTTCATTCCTC	GGAATGTTGTCTGGTTCAAGA	2404	0	0	0
Novel60	scaffold_103:171417:171509	−30.4	GCTTTCTCCCTCTAACTCGTCG	TCAAGTTAGAGAGAGAAAGCGT	0	14	7	0
Novel61	scaffold_12:3883367:3883573	−86.8	GAGCTTTCTTCGGTCCACTT	TTGGACTGAAGGGAGCTCCC	0	42	0	0
Novel62	scaffold_13:4483423:4483508	−22	CTACTTGGTAGGATACTTGGG	TGAGTATTTTCTACTGAGTGGCT	0	98	0	0
Novel63	scaffold_15:395989:396110	−25.9	TACAACTGTGGCAAGAAATGGCA	TCATCAAATGCTCAAGGGTGTGTAGC	0	17	0	0
Novel64	scaffold_17:2390469:2390560	−63.9	CACGCGGCCATCTCTCATTGAG	CAATGAGAGCTGGCCATGTGGG	0	29	0	0
Novel65	scaffold_26:118376:118514	−42.2	CGAGCAGATCCTGACACGCTT	GCATGCCTTAGAAATTTTTCGAT	0	15	0	0
Novel66	scaffold_37:247375:247555	−38.5	TCCTATGAGAGTTCGCGATTTTA	AAATTACGTTCTCGTTGGGTA	0	33	0	0
Novel67	scaffold_39:1309710:1309970	−39.04	AGCAGATGATGATACAAAAACA	TTGTTGTAAGCAGAGTTTGTGGA	0	8	0	0
Novel68	scaffold_3:1219690:1219806	−26.2	TGCTGTGATGATTTATGAATGAAT	ATATTCGTAATTCTGAGCAGT	0	26	0	0
Novel69	scaffold_40:130452:130527	−18	TAAAGAGGGTAAAATAGCTAACA	TTAGTTATTTCCGTCTACAAA	0	21	0	0
Novel70	scaffold_43:656273:656366	−33.24	TCAACTCGGCGATGCATTAATTA	CTTAATGCATCGCCAAGTTGAAT	0	13	0	0
Novel71	scaffold_44:542896:542995	−53.5	TGCCTGGCTCCCTGTATGCTT	GCGTGCGAGGAGCCATGCATG	0	11	0	0
Novel72	scaffold_46:1225861:1225959	−54.3	TCATACACAACCAATAAGAACC	TTCTTATTGATTGTGTATGGTA	0	8	0	0
Novel73	scaffold_56:198262:198499	−51.7	AATTCGATTGGTAGATGGGTA	CCTGATTTAATCAATGAGTTGG	0	15	0	0
Novel74	scaffold_5:7200012:7200132	−24.8	GGCAATTCAGAGGCTCAGAGTAT	ATTTCGATCGTCATTGCCAA	0	9	0	0
Novel75	scaffold_8:587750:587857	−65.1	TTGAACGTAATATACACACAC	GTGTGTATGTTACGTTCAACG	0	26	47	0

**Figure 3:**
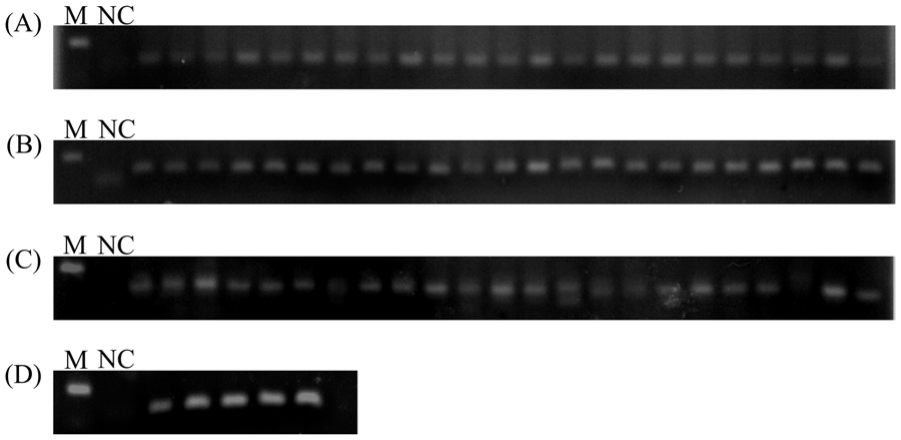
Stem-loop RT-PCR analysis of novel miRNAs expression with 30 cycles. (A) line 2–24 indicated novel miRNA from novel01 to Novel24; (B) line 2–24 indicated novel miRNA from novel25 to Novel50; (C) line 2–24 indicated novel miRNA from novel51 to Novel74; (D) line 2–6 indicated Novel75, Csi-miR156, Csi-miR172, miR396 and PtmiR93; NC indicated negative control; M indicated 100 bp.

All almost novel miRNAs had low read counts comparison with conserved miRNAs in this study, to confirm the expression of these novel miRNAs, these novel miRNAs were verified using PCR-based directed cloning approach. Out of 75 novel miRNAs tested (Figure 3, Supporting Information S1), 70 could be verified by sequencing, whereas five could not be found (Novel9, Novel47, Novel48, Novel49 and Novel65). For miRNAs that could not be detected by stem-loop RT-PCR, their precursors were detected using PCR. Of the 70 verified miRNAs by PCR-based directed cloning approach, 68 miRNA sequences showed completely consistent with deep sequencing, two miRNA sequences were detected length difference (Novel11 was found to have a single nucleotide addition, and Novel55 was observed had two nucleotides added compared with deep sequencing). For non-amplified products of miRNA, there were three possible explanations: First: we did not find a suitable primers and reaction conditions. Second: the expression of these miRNAs may be very low. Thirdly, these miRNAs might be hard to sequence due to physical properties or posttranscriptional modifications such as methylation. In addition, 31 miRNA precursors were also tested; the electrophoresis of the PCR products confirmed the size and expression of these novel miRNA precursors. All precursor amplicons were also verified by sequencing in this study. Therefore, these miRNAs were authentic miRNAs. These results suggested that Solexa sequencing was capable of successfully discovering novel miRNAs from this species with high accuracy and efficiency.

### Expression Profiling of Conserved and Novel miRNAs

**Figure 4:**
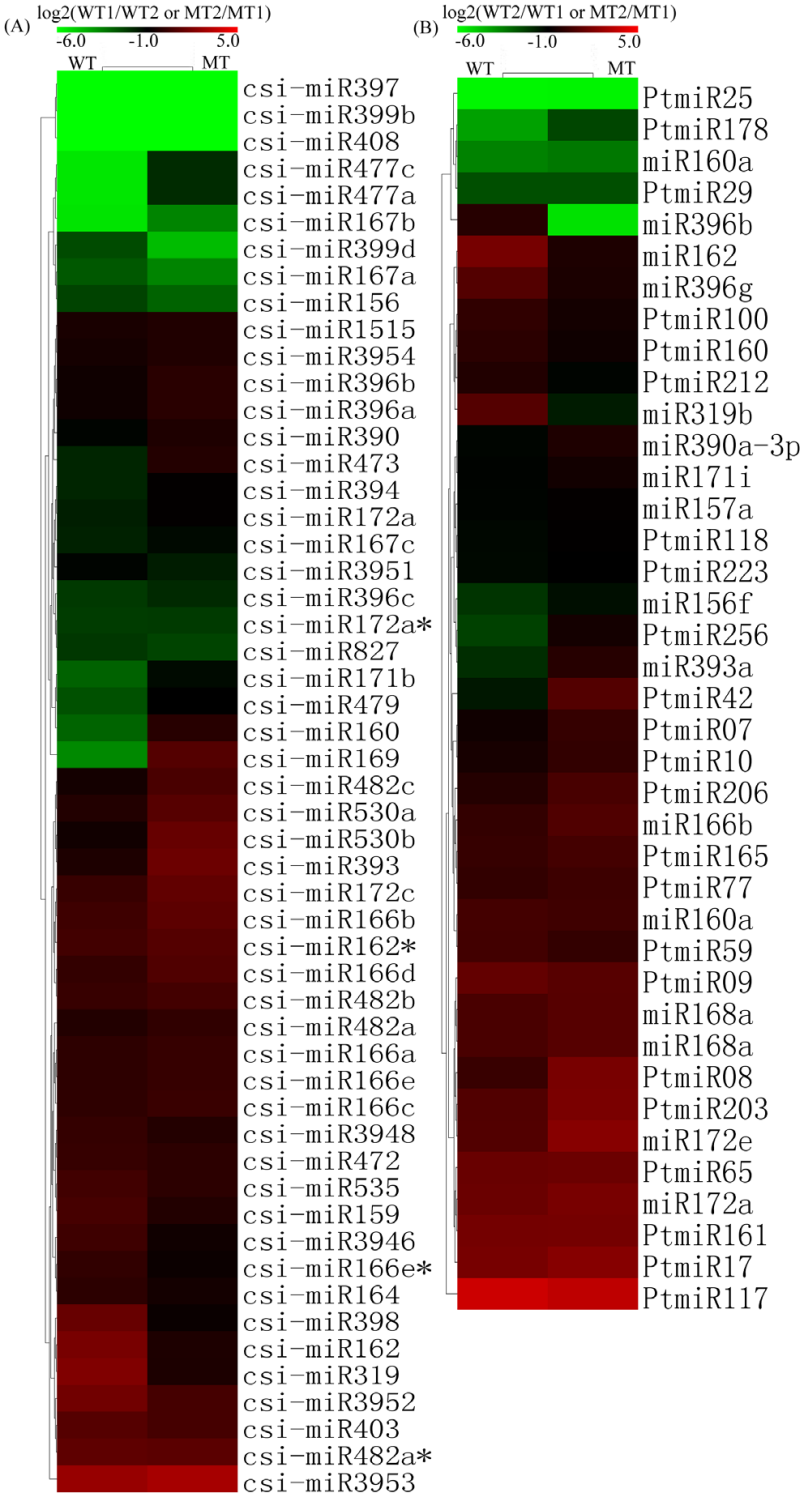
Expression patterns of the known miRNAs at different developmental stages. Several miRNA*s were included. Unsupervised hierarchical cluster analysis of two set array data (MT and WT), each column represents a sample, and each row represents a single miRNA. The bar represented the scale of relative expression levels of miRNAs, and colors indicate relative signal intensities.

We sequenced 141 known miRNAs (78 conserved and 63 known trifoliate orange-specific miRNAs) and 75 predicted novel miRNAs in this study. Among 78 conserved miRNAs, 43 miRNAs were down-regulated and 33 miRNAs were up-regulated at adult development stage of the MT (Figure 4, Supporting Information S2). In the WT, 43 miRNAs were also down-regulated and 32 miRNAs were up-regulated. Thus, down-regulation of miRNAs appeared to be more important during adult development process, plant miRNAs generally direct endonucleolytic cleavage of mRNAs [1], [41], [42], consistent with the suggestion that plant miRNAs enable rapid clearance of target mRNAs at specific points during plant development. The same expression patterns of most conserved miRNAs were obtained in two genotypes, indicating that they may perform a similar role during the flowering process. However, 22 miRNAs shown inverse expression patterns between the MT and the WT, the result shown that 10 miRNAs were up-regulated and 12 miRNAs were down-regulated at adult stage compared with the WT (Figure 4, Supporting Information S2). These results suggested that these miRNAs might be correlated with floral induction and flowering. Total conserved miRNA reads of the WT library was 1.4-fold greater than the MT library, which suggested that conserved miRNAs might had important functions during vegetative growth. Differential expression greater than two-fold between the two libraries (Ratio>2 or <0.5) was found for 47 miRNAs in the MT and 39 in the WT (Supporting Information S2). Several studies indicate that miRNA156/157 targets squamosa promoter binding protein (*SPL*) genes [23], [43], a plant-specific family of transcription factors involved in early flower development and vegetative phase changes [44]. MiR159 was implicated in floral and anther development by targeting the expression of *MYB33* and *MYB55* [45]. Meanwhile, most miRNAs were negatively correlated with their targets, being consistent with their functions in guiding the cleavage of target mRNAs in plants. In this study, the miR156/157/159 show significant down-expression from juvenile to adult in the MT compared with the WT, consistent with previous reports on the three miRNAs.

Among the known trifoliate orange-specific miRNAs, most miRNAs were obtained in both the MT and WT, except that 7 miRNAs were only detected in two MT libraries, and 4 miRNAs were only detected in two WT libraries (Figure 4B, Supporting Information S2). Of these miRNAs, 24 and 25 miRNAs were down-regulated and 35 and 29 miRNAs were up-regulated in the MT and the WT, respectively (Supporting Information S2); Twenty-nine miRNAs were expressed in inverse patterns between the MT and the WT, the results shown that 15 miRNAs were up-regulated and 9 miRNAs were down-regulated at adult stage compared with the WT. For novel miRNAs, most miRNAs were only obtained in the MT or the WT. For example, 17 miRNAs were only detected in two MT libraries, and 23 miRNAs were only detected in two WT libraries (Table 1). This was also the case for novel and conserved miRNA members in some miRNA families, partly because the relevant miRNAs might be repressed from the adult to the reproductive phase. In addition, all the novel miRNA reads had low read counts comparison with conserved miRNAs in the four libraries, where the highest was ranged from 1,867,066 to 4,155,155 reads (e.g. Novel56), and the least was only ranged from 0 to 6 reads (e.g. Novel50). It is well known that conserved miRNAs are highly expressed frequently and ubiquitously whereas non-conserved miRNAs are not. Further experimentation is needed to determine whether these novel miRNAs involved in early flowering of the MT.

### Expression Patterns of Conserved and Newly Identified miRNAs

**Figure 5:**
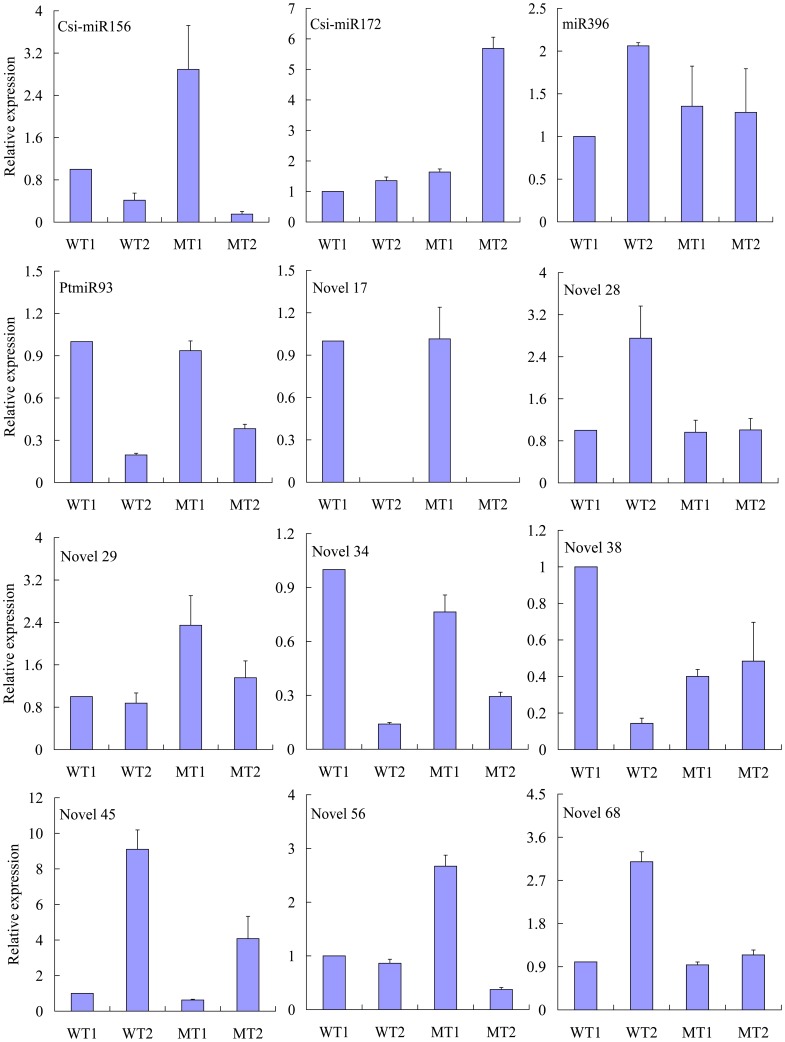
Expression analysis of conserved and novel miRNAs in the MT and its WT by real-time PCR. In the MT, Juvenile June: MT1; Adult April next year: MT2; In the WT, Juvenile June: WT1; Adult April next year: WT2. Csi-miR156, Csi-miR172 indicated *Citrus sinensis* conserved miRNAs; miR396 indicated other plants conserved miRNAs; PtmiR93 indicated known trifoliate orange-specific miRNAs; Novel17, Novel28, Novel29, Novel34, Novel38, Novel45, Novel56, and Novel68 indicated novel miRNAs. Data points represent the mean ± SE of at least four replications for the relative expression, which were calibrated by the amount of the *β-actin* control expression.

Knowledge about the expression patterns of miRNAs might provide clues about their functions. To understand the molecular mechanism of early flowering development process, and identify the expression pattern of the newly candidate miRNAs, the miRNA levels was investigated at different developmental stages of the MT and its WT by real-time PCR (Figure 5). Twelve miRNAs (including two *Citrus sinensis* known miRNAs, one other species miRNA, one trifoliate orange-specific miRNAs, and eight novel miRNAs) were selected in this study. These selected miRNAs previously reported to be associated with, or involved in, flowering developmental processes in other species, or their transcript levels were significantly changes (up- or down-regulated) at two stages. The miRNA abundance patterns of the MT and WT were compared with Solexa sequencing data. Results showed that for 10 of the 12 miRNAs, real-time PCR revealed the same expression patterns as the RNA-Seq data, despite some quantitative differences in expression level (Figure 5). Previous studies have shown that miR156 decreases during phase development in *Arabidopsis* [46], whereas miR172 increases [47], [48]. In this study, miR156 and miR172 were expressed in inverse patterns: miR156 declined from juvenile to adult stage whereas miR172 increased during this same period, consistent with previous reports on the two miRNAs.

### Target Prediction of miRNAs

It has been shown that plant miRNAs exhibit a high degree of sequence complementarity to their targets, which allows for effective target prediction [49]. To better understand the functions of the newly identified species-specific miRNAs, putative targets of these miRNAs were predicted using the described criteria and methods. Among the 75 novel identified miRNAs, 51 miRNAs had targets predicted. A total of 317 potential target genes were predicted (Supporting Information S5). Most miRNA families have multiple target sites, suggesting that these miRNAs are functionally divergent. On the other hand, a single gene may potentially be also targeted by several miRNAs, 8 genes have more than two miRNA-complementary sites located in full-length cDNA sequence in this study, such as clementine 0.9_002420m, which were likely to be targeted by Novel27 and Novel59, were found to have two complementary sites. Interestingly, clementine 0.9_002772m, which was likely to be targeted by Novel53, was found to have two complementary sites in the coding region (Supporting Information S5). Finally, 325 target sites were predicted based on these novel miRNAs.

In plants, the miRNA target sites were found predominantly in the coding regions [50]. Consistent with these findings, 276 of our predicted target genes have target sites in the coding region; 4 target genes have miRNA complementary sites in 3' UTRs whereas 46 target genes were found to have miRNA target sites in 5' UTRs. The analysis of novel miRNAs targets showed that a high proportion of the targets were transcription factors, including GATA transcription factor for Novel11, GRAS family transcription factor for Novel53, disease resistance protein family for Novel49, auxin response factor for Novel71 and Novel34, and squamosa promoter binding protein for Novel56. Besides transcription factor targets, targets involving defense response, transcription, chromatin remodeling, hormone regulation and other metabolic pathways were overrepresented (Supporting Information S5). These observations suggested that miRNA targeted genes in trifoliate orange play roles not only in development but also in diverse physiological processes.

### Annotation of Potential Targets of miRNAs

**Figure 6:**
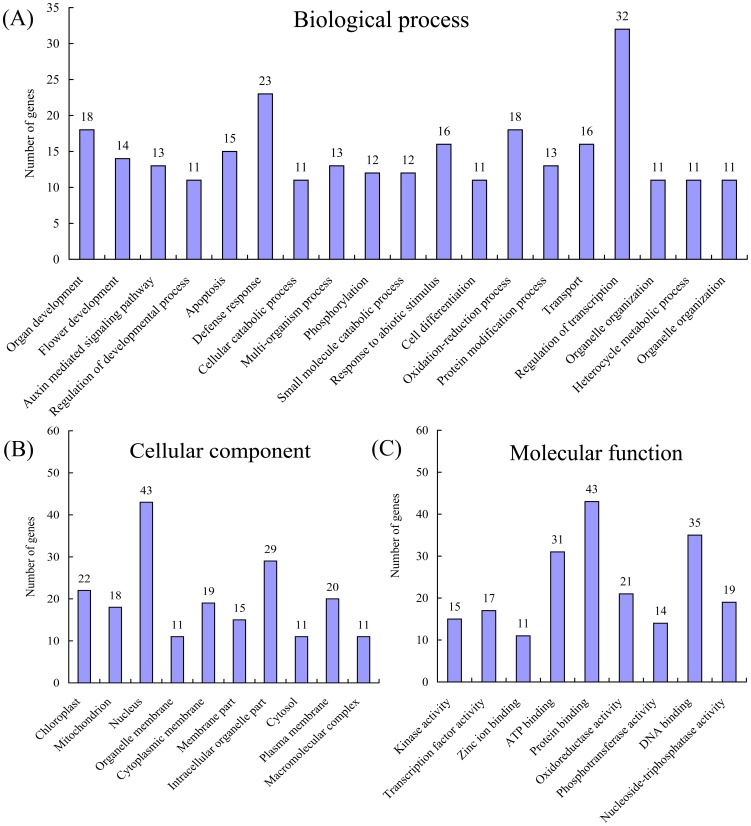
Gene ontology categories of target genes of the 51 novel miRNA families. Categorization of miRNA-target genes was performed according to the biological process (a), cellular component (b), and molecular function (c).

To evaluate the potential functions of these miRNA-target genes, gene ontology (GO) categories were assigned to the putative targets of the 51 novel miRNAs according to the method described by Morin et al. [51]. Figure 6 summarized the categorization of miRNA-target genes according to the biological process, cellular component and molecular function. Based on biological process, these genes were finally classified into 20 categories, as shown in Figure 6A; the most four over-represented GO terms are regulation of transcription (32 genes), defense response (23 genes), organ development (18) and oxidation-reduction process (18). Interestingly, 14 and 11 genes were involved in flower development and regulation of development process, respectively. These results suggested that citrus miRNAs were involved in a broad range of physiological functions; it will be an interesting area to identify the functions of these predicted target genes in citrus. Categories based on cellular component revealed that the miRNA-target genes were related to 10 cellular parts (Figure 6B); the most three frequent terms were nucleus (43 genes), intracellular organelle part (29) and chloroplast (22). The most three over-represented GO terms were protein binding (43 genes), DNA activity (35) and ATP activity (31) based on molecular function (Figure 6C). In addition, the biological interpretation of these target genes was further examined using KEGG pathway analysis. A total of 57 different pathways were found, of which some were consistent with biological processes already revealed by GO analysis. The most frequently represented pathways included purine metabolism pathways (12 enzymes represented), thiamine metabolism (8), starch and sucrose metabolism (6), amino sugar and nucleotide sugar metabolism (3), and fatty acid metabolism (3).

### Monitoring Expression of Potential mRNA Targets of miRNAs

**Figure 7:**
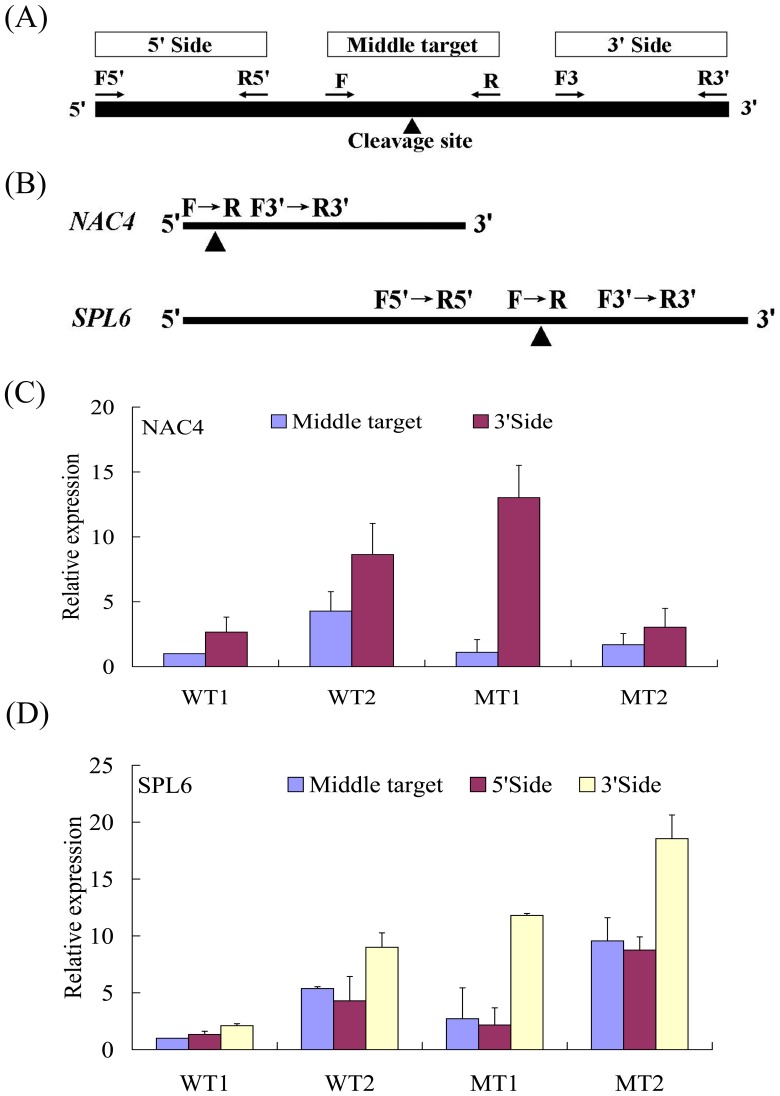
Diagram of regional amplification quantitative RT-PCRs (RA-PCR) and Stage-specific RA-PCR of miRNA-targeted *NAC4* and *SPL6*. (A) Diagram of primers designed to amplify fragments of cleaved and noncleaved miRNA (miRNA)-targeted mRNA present in tissues. miRNA target sites (▴); (B) Real-time PCR for sense and antisense expression of selected miRNA targets; (C) Relative quantification of two fragments of *NAC4* (target of Novel29) by RA-PCR with the middle target fragment being set to a value of 1; (D) Relative quantification of two fragments of *SPL6* (target of Novel56) by RA-PCR with the middle target fragment being set to a value of 1. Data points represent the mean ± SE of at least four replications for the relative expression, which were calibrated by the amount of the *β-actin* control expression.

Quantitative RT-PCR was employed to assess the abundance of three regions of several mRNAs. Figure 7(A) illustrates the relative positions of the three primer sets for a given mRNA for the RA-PCR method. The first set (F5′, R5′) amplifies a region upstream from the potential cleavage site. The second set (F, R) synthesizes a fragment that contains the target site and flanking regions, and the third set (F3′, R3′) amplifies a fragment downstream of the target site. Among the above eight novel miRNAs, only four miRNAs had targets predicted (Novel17, Novel28, Novel29 and Novel56). We initially examined all potential transcription factor targets of four miRNAs by using end-point PCR method. The amount of PCR products for the 5′, middle, and 3′ regions of the Novel17 and Novel28 were similar for all tissues and stages tested (data not shown). Differences were noted for these regions in the *SPL6* and *NAC4* mRNAs, which merited more detailed analyses. Figure 7(C) shows the products of the RA-PCR reactions for the *NAC4* mRNA, a potential target of Novel29 from juvenile stage to adult stage of two genotypes. For *NAC4*, only middle region was investigated because of its target site in 5' UTRs, the target region was decreased relative to the 3′ regions. The amount of product of the middle region was 0.08 to 0.51 of the amount observed for the 3′ region. This is consistent with miRNA-induced cleavage of this mRNA. Figure 7(D) shows the results from RA-PCR analysis of the three regions amplified from *SPL6*. The ratio of product of the middle region to the 3′ region varied from 0.23 to 0.47. This is again in agreement with miRNA-induced cleavage of this mRNA. These results indicated that *SPL6* and *NAC4* may be regulated by Novel29 and Novel56, respectively.

### Expression Pattern of Conserved and Newly Identified miRNAs during Self-pruning

**Figure 8:**
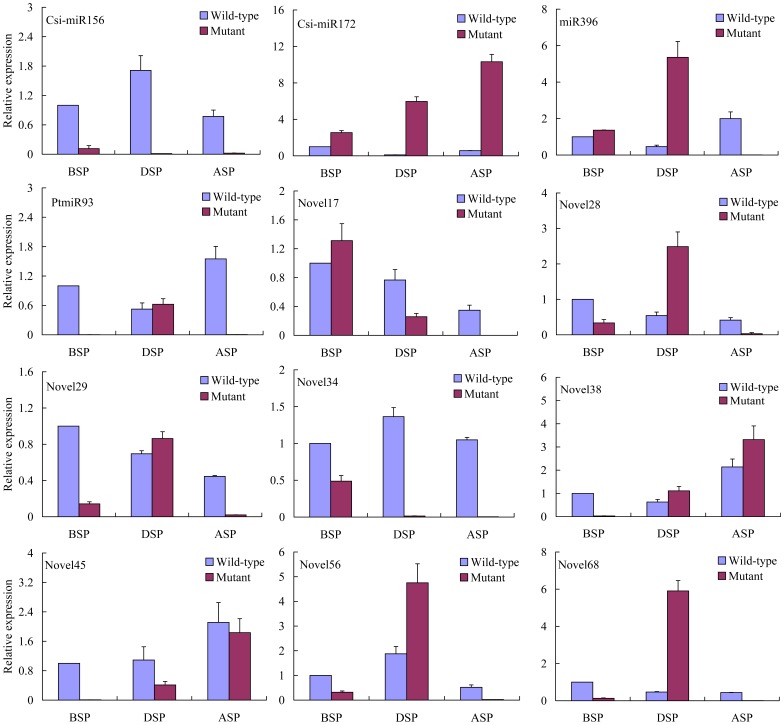
The expression pattern of conserved and novel miRNAs during self pruning. The relative quantities of 12 miRNAs during floral transition in spring shoots of both the MT and the WT were investigated by real-time PCR. *β-actin* was used as a housekeeping control. Real-time PCR experiments were conducted using the primers displayed in Supporting Table 1. BSP: 15 days before self-pruning; DSP: during self-pruning; ASP: 20 days after self-pruning.

Self-pruning is a necessary but not sufficient condition for floral bud initiation. Cytological observation revealed that the floral buds in the two-year-old MT initiated their differentiation immediately after self-pruning on spring shoots. In two-year-old WT, the spring shoots, which do not form floral buds, begin to produce vegetative buds after self-pruning. Thus, the self-pruning appears to be a demarcation point for the meristem to initiate leaf bud or floral bud development. To understand the role of miRNAs in the floral transition, the dynamic expression of above twelve miRNAs during self-pruning was monitored by real-time PCR. As shown in Figure 8, miR156 expression was markedly down-regulated during the floral transition and miR172 increased to higher levels after the lateral buds were differentiated (20 days after self-pruning) in the flowering-competent shoots (spring shoots of the MT). However, in the flowering incompetent shoot (spring shoots of the WT), miR156 and miR172 were expressed in inverse patterns during self-pruning compared with the prior stage. The dynamic expression of miR156 and miR172 in flowering-competent and -incompetent shoots suggested that they were involved in the floral transition (Figure 8). Of the other miRNAs, the Novel34 and Novel38 expression patterns were similar to that of miR156 and miR172, indicating that they may perform a similar role during the flowering process (Figure 8). In *Arabidopsis*, miR396-targeted *AtGRF* transcription factors were required for coordination of cell division and differentiation during leaf development, In MT tissue, they showed high transcript levels at the undetermined stage (from before self-pruning to during self-pruning), the amount decreased at the floral bud initiation stage. However, compared with the levels in MT tissue, the miR396 was also detected and present at low levels in the WT tissue, followed by increasing a high level of expression at the floral bud initiation stage. A similar trend of changes in transcript level was seen in Novel28, Novel56 and Novel68 (Figure 8). One possible explanation for this observation is that these miRNAs may play a similar role in inducing early flowering in precocious trifoliate orange.

## Discussion

Nowadays, characterization of the vital roles of miRNAs play in plant development is an active research field. Although many studies have demonstrated that plant miRNAs function as important regulators in environmental stress tolerance, more reports indicated that plant miRNAs were also involved in development and morphogenesis processes. Deep sequencing of the small RNA transcriptome yields an incredible amount of data, from which we can not only determine known miRNAs, but also successfully explore novel miRNAs with high accuracy and efficiency. Several studies have reported on miRNAs from other economically important tree crops by deep sequencing. More recently, deep sequencing was used to analyze a sRNA library generated from *Poncirus trifoliata* flowers and fruit. Sixty-three sequences of 42 highly conserved miRNA families and 10 novel miRNAs were identified based on citrus EST database [52]. Likewise, Xu et al. (2010) constructed sRNA libraries from fruit of a red flesh bud sport and its wild type in sweet orange. Deep sequencing identified 85 known miRNAs (48 families) and 12 novel miRNAs [16]. Several sequencings of small RNA libraries from different grapevine cultivars or tissues have been also reported in recent years [53], [54]. For example, Pantaleo et al. (2010) used deep sequencing, computational and molecular methods to identify, profile, and describe 40 known miRNA families and 21 novel miRNA candidates in grapevine tissues [54]; Wang et al (2012) identified 72 new potential miRNAs and 34 known but non-conserved miRNAs by deep sequencing of short RNAs from Amur grape flowers and fruits [53]. In a separate study, a total of 130 conserved grapevine miRNA belonging to 28 miRNA families were validated, other 80 unconserved miRNAs including 72 novel potential and 8 known but unconserved ones were found by deep sequencing [55]. However, there was a relatively large number of citrus miRNAs compared with grapevine. On the other hand, the number of miRNA was significantly fewer based on EST database than genome-wide analysis by deep sequencing. Thus, the recent release of the genome sequence will dramatically enhance the efficiency of discover new miRNAs in citrus. In this study, the use of Solexa sequencing technology was efficient to identify 216 known and novel miRNAs of trifoliate orange base on citrus genome. These miRNAs were identified from four different libraries from precursors with stem-loop secondary structures that also map to the citrus genome. They were detected from the MT and WT libraries and were characterized as following: detected for the first time, already detected in some plant species, conserved in citrus, or a variant of a known miRNA (isoform). From these analyses, we found 75 novel miRNAs that had not been detected before, 36 families that had already been detected in *Citrus sinensis*, 67 trifoliate orange-specific miRNAs and 24 conserved miRNAs in other plants. Previous studies validated the conserved miRNAs based on homology to known miRNAs in miRBase. However, as opposed to some studies that only blast the candidate to the known miRNA mature sequence, our identifications were determined by precursor sequence folding and verification of the genuine hairpin structures.

Both miRNAs and miRNA*s are generated from step-loop hairpin structures. It has been widely accepted that after processing from precursors, the functional strand of the short double-stranded RNA duplex will be retained as miRNAs, whereas their complementary miRNA* sequences will be quickly degraded [56]. MiRNAs are stable and participate in translational repression or cleavage of mRNA by binding or anchoring to the coding region of mRNA sequences [11]. Khvorova et al inferred from the considerably low abundance of miRNA*s that these strands are typically destroyed when released from pre-miRNA stem [56]. Therefore, in most cases, the abundance of miRNAs is much higher than that of their corresponding miRNA*s. In this study, the low expression levels of miRNA*s sequences, such as csi-miR162*, csi-miR166e*, and csi-miR172a*, further support the miRNA synthesis hypothesis. The correlation between miRNA*s and its flexible expression may reveal its particular regulated function. Csi-miR482* may be involved in regulating phase transition. Two arms of a single hairpin, giving rise to RNA function isolation by different sequences, may associate with distinct biological activities.

Some highly conserved miRNA families such as miR156/157, miR167 and miR172 families were sequenced more than ten thousands or even one hundred thousands times. These highly conserved miRNAs may represent a relationship between evolutionary conservation and expression abundance [57]. The other way around, some miRNA families that are less conserved or even species-specific have very low abundance in two genotypes. From an evolutionary view, these miRNAs play a role in establishing and maintaining phenotypic diversity between different groups of organisms [57], [58]. It is plausible that the conserved miRNAs are responsible for control of the basic cellular and developmental pathways common to most eukaryotes whereas the non-conserved miRNAs are involved in regulation of the species-specific pathways and functions [57]. In addition to the identification of conserved miRNAs, 75 novel miRNAs were also identified. Only one member was identified in each species-specific miRNA family and the read number for each novel miRNA was much lower than that for the conserved miRNAs except Novel56. The low abundance of novel miRNAs might suggest that they in their turn play rather specific roles in response to a particular growth condition or a particular developmental stage [59]. In addition, deep sequencing technologies in combination with bioinformatics analysis enabled us to profile the miRNA expression patterns for further miRNA functional insights, and to elucidate the underlying molecular mechanisms and diverse physiological pathways. Second, comparing miRNA expression profiles at different developmental stages, we found significant differences in miRNA regulation patterns, with 22 conserved miRNAs altered expression patterns between the MT and its WT at juvenile and adult stage. All almost trifoliate orange-specific miRNAs (including known and novel miRNAs) had low read counts comparison with conserved miRNAs in this study, there was no significant difference on miRNAs profile between the MT and the WT for most miRNAs. There was one possible explanation why only a small number of miRNAs showed significant differences in two genotypes. Because the MT and its WT have nearly the same morphology except flowering habit [21], molecular evaluation on DNA level using a number of AFLP markers produced no polymorphism between the MT and its WT, indicating an isogenic background between them [60]. Thus, we believed that a small number of miRNAs or specific flowering-related genes from the MT (these miRNAs and genes may be key regulators controlling flowering by activating or repressing numerous flowering related genes) may be control the early flowering process. The differentially expressed miRNAs obtained can serve as a basis for further identification of the regulation roles of phase transition in citrus.

More recent studies have demonstrated that miRNAs in *Arabidopsis*, rice and other plant species target transcripts encoding proteins involved in diverse physiological processes [61], among which a set of miRNAs predominantly targeted transcription factors. Many known transcription and post-transcriptional regulatory genes were found in our library, and some known transcription factors related to flowering regulation were also found, including NAC domain transcription factor, ring zinc finger, squamosa promoter binding protein, and MYB transcription factor. NAC domain proteins were a class of transcription factors known to control multiple processes in plants, including the development of flower organ primordium [62] and flowering time [63]. Many (putative) zinc-finger transcription factors have also been implicated in flower development [64], flowering time [65], and light-regulated morphogenesis [66]. Other target genes include those encoding o-fucosyltransferase family protein, disease resistance protein family, FAR1-related sequence, LRR and NB-ARC domains-containing disease resistance protein and protein of unknown function, suggesting that trifoliate orange miRNAs were involved in a broad range of physiological functions. It would be an interesting area to identify the functions of these predicted target genes in trifoliate orange. In addition, it was hypothesized that a complementary site in the coding region of an mRNA may lead to the cleavage of the target, whereas target sites located in 3 UTRs might attenuate translation (Bartel, 2004). Consistent with this view, trifoliate orange miRNAs that target ORFs have largely been shown to be involved in the cleavage of target mRNAs. A signature of an mRNA with a target site cleaved by a miRNA-guided process was a decrease in the RT-PCR amplification of any fragment that contained a region which was upstream of the target site [34]. *NAC4* and *SPL6* mRNA may be the potential targets for Novel29 and Novel56 by RA-PCR approach, respectively. The RA-PCR technique for monitoring miRNA-guided cleavage of mRNAs had an advantage of including control reactions in the same experiment. However, RA-PCR also may produce uncertainties in data interpretation as a result of the presence of additional primer-binding sites, self-priming caused by RNA secondary structures, or unusual patterns of mRNA degradation [67].

Plant miRNAs have been reported as involving in morphology and flowering time [68], [69]. Are miRNAs the regulators in those processes? Although extensive experimentation would be needed to answer this question, at least the identity of the possibly novel miRNAs in this paper gives some potential gene targets for further investigation of the molecular basis of the regulation of growth and development in trifoliate orange. For example, molecular genetic analyses of vegetative phase change in maize and *Arabidopsis* have since revealed that miR156 plays a particularly important role in this transition [17], [70], [71], miR156 is expressed at very high levels during the juvenile phase and declines in abundance during vegetative phase change. Constitutive expression of miR156 prolongs the expression of juvenile traits, whereas loss of miR156 activity eliminates these traits, demonstrating that miR156 is both necessary and sufficient for the juvenile phase [68], [69]. MiR172 has also been implicated in the regulation of flowering time and floral organ identity in both maize and *Arabidopsis* [8], [19]. Aukerman et al (2003) demonstrated that miR172 causes early flowering and disrupts the specification of floral organ identity when over-expressed in *Arabidopsis* [47]. More recently, Song et al. (2010a) analyzed miR172 and miR156 from trifoliate orange by RACE and verified their expression patterns. Expression profiles of miRNAs indicated that the regulation mechanisms of these miRNAs were conserved in citrus. Previous studies have shown that miR156 decreases during phase development in *Arabidopsis* [46], whereas miR172 increases [47], [48]. In this study, to understand the role of miRNAs in the floral transition, the dynamic expression of twelve miRNAs during phase change and self-pruning process was monitored by real-time PCR. The fluctuation in miR156 and miR172 expression was quite significant compared with other miRNAs. Meanwhile, miR156 and miR172 were expressed in inverse patterns, consistent with previous reports on the two miRNAs. These results suggested that miR156 and miR172 not only serves as a master regulator of vegetative phase change, but as a molecular marker for this process. In addition, Wang et al. (2011) also elucidated the role of miR156 to decide the age of branch at different position and make sure the mature branch to produce the fruits [72]. It reveals that self-pruning and aging of branch were both important processes for the floral initiation in tree and may share some similar regulatory mechanism among them.

## Conclusions

The present study detected a large number of small RNA sequences that were characterized as novel and as already known citrus miRNAs. Our current study introduces an accurate and efficient approach for miRNA discovery and will aid the identification of many miRNAs in other species. We grouped some of these unique sequences into 75 novel miRNAs and further classified several of new members in known families or as new loci in citrus genome. We present an expression profile of these miRNAs at phase transition from vegetative growth to generative growth, highlighting negative correlation between miRNA and target mRNA accumulation. More importantly, based on our analysis of the small RNA expression levels in juvenile and adult development stage, we believe that some miRNAs that regulate the expression of protein coding genes in the two phases must be involved in the process of phase changes. Future investigations should use supplementary experimental approaches to verify the targets and to understand the complex gene regulatory network of these miRNAs. It is possible that we could provide insight into the phase changes and find new approaches to understanding of the regulatory mechanisms for citrus flowering development.

## Supporting Information

Supporting Information S1 **The primer sequence information.** This file lists the primers used for miRNA expression detection by stem-loop RT-PCR.
(DOC)

Supporting Information S2 **The conserved miRNA sequence information.** Supporting Table 1: 54 known miRNAs corresponding to 36 miRNA families in *Citrus sinensis*; Supporting Table 2: 24 known miRNAs corresponding to 14 miRNA families in other species; Supporting Table 3: 63 known trifoliate orange-specific miRNAs were detected in four libraries by deep sequencing. WT1, WT2, MT1 and MT2: expression of the miRNAs was detected at different stages; nt, nucleotides. MFE: minimal folding free energy; Location: miRNA location in genome; Trifoliate orange sequence: the cloned miRNA sequences by deep sequencing.
(XLS)

Supporting Information S3 **Fold-back structures for conserved miRNA.** Precursor secondary structures and dG value were produced using the mfold software. http://mfold.bioinfo.rpi.edu/. MiRNA and miRNA* sequences are highlighted in red and blue, respectively. The numbers along the structure are nucleotide sites from the 5′ end of the pre-miRNA sequence.
(DOC)

Supporting Information S4 **Fold-back structures for novel miRNA.** Precursor secondary structures and dG value were produced using the mfold software. http://mfold.bioinfo. rpi.edu/. MiRNA and miRNA* sequences are highlighted in red and blue, respectively. The numbers along the structure are nucleotide sites from the 5′ end of the pre-miRNA sequence.
(DOC)

Supporting Information S5 **Target predictions of 51 novel miRNAs from the MT and its WT.** 5′UTR indicated target site located in the predicted 5′untranslated region; 3′UTR indicated target site located in the predicted 3′untranslated region; ORF indicated target sites are located in the predicted ORFs. “|” represents a base pair; “x” represents a mismatch; “o” represents a G-U basepair; the information between brackets represents energy information and miRNA/target duplex energy ratio.
(XLS)
