# Supplementary material for: Identification and Comparative Profiling of miRNAs in an Early Flowering Mutant of Trifoliate Orange and Its Wild Type by Genome-Wide Deep Sequencing
Source: PLoS One. 2012 Aug 28;7(8):e43760. doi: 10.1371/journal.pone.0043760 (PMC3429500; doi:10.1371/journal.pone.0043760)
Supplement: Supporting Information S1 — The primer sequence information. This file lists the primers used for miRNA expression detection by stem-loop RT-PCR. (DOC) [file pone.0043760.s001.doc]

NOVEL01 5-AGATACTCATTTGAGCTAGAAG-3

RT-primer：5-GTCGTATCCAGTGCAGGGTCCGAGGTATTCGCACTGGATACGACCTTCTA-3

Forward primer： 5-CAGCGAGATACTCATTTGAGC-3

Amplicon was verified by sequencing

CACCCGCCTCTGGTCGTTTAGAATGGGCTTCATTCAGTTCCGTTCCCCACGAGATCAGCGAGTTACATGTTCCCCATGTTGTGCAAAAAAGCGTAGTCCTTCGGTCTCGATCGTGTCAGAGTAGTGGCGCAGTGTTATCACTCCATGGTATGCAGCACTGCATAATTCTTCTACTGTCATGCCCATCCGTAAGATGCTTTTCCTGTGACTGGTGAGTACTCAACCAAGTCATTCTGAGAATAGTGTATGCGGCGACCGAGTTGCTCTTGCCCGGCGTCAATACGGGATAATACCGCGCCACATAGCAGAACTTTAAAAGTGCTCATCATTGGAAAACGTTCTTCGGGGCGAAAACTCTCAAGGATCTTACCGCTGTTGAGATCCAGTTCGATGTAACCCACTCGTGCACCCAACTGATCTTCAGCATCTTTTACTTTCACCAGCGTTTCTGGGTGAGCAAAAACAGGAAGGCAAAATGCCGCAAAAAAGGGAATAAGGGCGACACGGAAATGTTGAATACTCATACTCTTCCTTTTTCAATATTATTGAAGCATTTATCAGGGTTATTGTCTCATGAGCGGATACATATTTGAATGTATTTAGAAAAATAAACAAATAGGGGTTCCGCGCACATTTCCCCGAAAAGTGCCACCTGACGTCTAAGAAACCATTATTATCATGACATTAACCTATAAAAATAGGCGTATCACGAGGCCCTTTCGTCTCGCGCGTTTCGGTGATGACGGTGAAAACCTCTGACACATGCAGCTCCCGGAGACGGTCACAGCTTGTCTGTAAGCGGATGCCGGGAGCAGACAAGCCCGTCAGGGCGCGTCAGCGGGTGTTGGCGGGTGTCGGGGCTGGCTTAACTATGCGGCATCAGAGCAGATTGTACTGAGAGTGCACCATATGCGGTGTGAAATACCGCACAGATGCGTAAGGAGAAAATACCGCATCAGGCGCCATTCGCCATTCAGGCTGCGCAACTGTTGGGAAGGGCGATCGGTGCGGGCCTCTTCGCTATTACGCCAGCTGGCGAAAGGGGGATGTGCTGCAAGGCGATTAAGTTGGGTAACGCCAGGGTTTTCCCAGTCACGACGTTGTAAAACGACGGCCAGTGCCAAGCTTGCATGCCTGCAGGTCGACGATCAGCGAGATACTCATTTGAGCTAGAAGGTCGTATCCAGTGCGAATACCTCGGACCCTGCACAATCTCTAGAGGATCCCCGGGTACCGAGCTCGAATCGTAATCATGTCATTGTCCGC

NOVEL02 5-CTGGATTCAGCTGTGGTATGGTA-3

RT-primer：5-GTCGTATCCAGTGCAGGGTCCGAGGTATTCGCACTGGATACGACTACCAT-3

Forward primer： 5-CAGCGCTGGATTCAGCTGTGGT-3

Amplicon was verified by sequencing

TCGGACATGACATGATTACGATTCGAGCTCGGTACCCGGGGATCCTCTAGAGATTCAGCGCTGGATTCAGCTGTGGTATGGTAGTCGTATCCAGTGCGAATACCTCGGACCCTGCACAATCGTCGACCTGCAGGCATGCAAGCTTGGCACTGGCCGTCGTTTTACAACGTCGTGACTGGGAAAACCCTGGCGTTACCCAACTTAATCGCCTTGCAGCACATCCCCCTTTCGCCAGCTGGCGTAATAGCGAAGAGGCCCGCACCGATCGCCCTTCCCAACAGTTGCGCAGCCTGAATGGCGAATGGCGCCTGATGCGGTATTTTCTCCTTACGCATCTGTGCGGTATTTCACACCGCATATGGTGCACTCTCAGTACAATCTGCTCTGATGCCGCATAGTTAAGCCAGCCCCGACACCCGCCAACACCCGCTGACGCGCCCTGACGGGCTTGTCTGCTCCCGGCATCCGCTTACAGACAAGCTGTGACCGTCTCCGGGAGCTGCATGTGTCAGAGGTTTTCACCGTCATCACCGAAACGCGCGAGACGAAAGGGCCTCGTGATACGCCTATTTTTATAGGTTAATGTCATGATAATAATGGTTTCTTAGACGTCAGGTGGCACTTTTCGGGGAAATGTGCGCGGAACCCCTATTTGTTTATTTTTCTAAATACATTCAAATATGTATCCGCTCATGAGACAATAACCCTGATAAATGCTTCAATAATATTGAAAAAGGAAGAGTATGAGTATTCAACATTTCCGTGTCGCCCTTATTCCCTTTTTTGCGGCATTTTGCCTTCCTGTTTTTGCTCACCCAGAAACGCTGGTGAAAGTAAAAGATGCTGAAGATCAGTTGGGTGCACGAGTGGGTTACATCGAACTGGATCTCAACAGCGGTAAGATCCTTGAGAGTTTTCGCCCCGAAGAACGTTTTCCAATGATGAGCACTTTTAAAGTTCTGCTATGTGGCGCGGTATTATCCCGTATTGACGCCGGGCAAGAGCAACTCGGTCGCCGCATACACTATTCTTCAGATGACTTGGTTGAGTACTCCACCAGTCACAGGAAAGCATCTTACGGATGGCATGACAGTAGAGAATTATGCAGTGCTGCATAACAATGAGTGATACACTGCGCACTACTCCTGACACGATCGGAGAACGAAGAGCTACGGCTTTTTTGCACAACATGGGGATCTGTACTCGTCATGATCGATGGTTCGGGACTGAATGAAGCCATATACCA

NOVEL03 5-GCAAGTCGTCTTTGGCTAGTC-3

RT-primer：5-GTCGTATCCAGTGCAGGGTCCGAGGTATTCGCACTGGATACGACGACTAG-3

Forward primer： 5-CGCCGGCAAGTCGTCTTTGG-3

Amplicon was verified by sequencing

ACCGACGAGACATGATTACGATTCGAGCTCGGTACCCGGGGATCCTCTAGAGATTCGCCGGCAAGTCGTCTTTGGCTAGTCGTCGTATCCAGTGCGAATACCTCGGACCCTGCACAATCGTCGACCTGCAGGCATGCAAGCTTGGCACTGGCCGTCGTTTTACAACGTCGTGACTGGGAAAACCCTGGCGTTACCCAACTTAATCGCCTTGCAGCACATCCCCCTTTCGCCAGCTGGCGTAATAGCGAAGAGGCCCGCACCGATCGCCCTTCCCAACAGTTGCGCAGCCTGAATGGCGAATGGCGCCTGATGCGGTATTTTCTCCTTACGCATCTGTGCGGTATTTCACACCGCATATGGTGCACTCTCAGTACAATCTGCTCTGATGCCGCATAGTTAAGCCAGCCCCGACACCCGCCAACACCCGCTGACGCGCCCTGACGGGCTTGTCTGCTCCCGGCATCCGCTTACAGACAAGCTGTGACCGTCTCCGGGAGCTGCATGTGTCAGAGGTTTTCACCGTCATCACCGAAACGCGCGAGACGAAAGGGCCTCGTGATACGCCTATTTTTATAGGTTAATGTCATGATAATAATGGTTTCTTAGACGTCAGGTGGCACTTTTCGGGGAAATGTGCGCGGAACCCCTATTTGTTTATTTTTCTAAATACATTCAAATATGTATCCGCTCATGAGACAATAACCCTGATAAATGCTTCAATAATATTGAAAAAGGAAGAGTATGAGTATTCAACATTTCCGTGTCGCCCTTATTCCCTTTTTTGCGGCATTTTGCCTTCCTGTTTTTGCTCACCCAGAAACGCTGGTGAAAGTAAAAGATGCTGAAGATCAGTTGGGTGCACGAGTGGGTTACATCGAACTGGATCTCAACAGCGGTAAGATCCTTGAGAGTTTTCGCCCCGAAGAACGTTTTCCAATGATGAGCACTTTTAAAGTTCTGCTATGTGGCGCGGTATTTATCCCGTATTGACGCCCGGGCAAGAGCAACTCGGTCGCCGCATACACTATTCTCAGATGACTTGGTTGAGTACTCACCAGTCACAGAAAAGCATCTTACGATGGCATGACAGTAGAGAATTATGCAGTGCTGCATATCAATGAATGATACACTGCGCACTTACCTCTGACACGATCGAGACGAGGAGCTACGCTTTTGCACAACATGGGGGAATCGTACTCGCTGATCGTGAACCGGACCTGAATGAGCATACCTAACGACGAGCGCGTGAAC

NOVEL04 5-ATGTGCAATAGGTCAATGTTAGG-3

RT-primer：5-GTCGTATCCAGTGCAGGGTCCGAGGTATTCGCACTGGATACGACCCTAAC-3

Forward primer： 5-CATCGATGTGCAATAGGTCAAT-3

Amplicon was verified by sequencing

GTCTTTGTGTCCGTTGGTTAATGGCTTCATTTCAGTCCAGTTCCACCGATTCCAGCGAGTTACATGATCCCCCCATGTTGTGCAAGAAGCCGTAGCTCCTCGGTCCTCCGATCGTGTCAGAGTAGTGGCGCAGTGTATCACTCCATGTATGCAGCACTGCATAATCTCTACTGTCATGCCATCCGTAGATGCTTTTTCTGTGACTGGGGAGTACTCAACCAAGTCATTCTGAGAATAGTGTATGCGGCGACCGAGTTGCTCTTGCCCGGCGTCAATACGGGATAATACCGCGCCACATAGCAGAACTTTAAAAGTGCTCATCATTGGAAAACGTTCTTCGGGGCGAAAACTCTCAAGGATCTTACCGCTGTTGAGATCCAGTTCGATGTAACCCACTCGTGCACCCAACTGATCTTCAGCATCTTTTACTTTCACCAGCGTTTCTGGGTGAGCAAAAACAGGAAGGCAAAATGCCGCAAAAAAGGGAATAAGGGCGACACGGAAATGTTGAATACTCATACTCTTCCTTTTTCAATATTATTGAAGCATTTATCAGGGTTATTGTCTCATGAGCGGATACATATTTGAATGTATTTAGAAAAATAAACAAATAGGGGTTCCGCGCACATTTCCCCGAAAAGTGCCACCTGACGTCTAAGAAACCATTATTATCATGACATTAACCTATAAAAATAGGCGTATCACGAGGCCCTTTCGTCTCGCGCGTTTCGGTGATGACGGTGAAAACCTCTGACACATGCAGCTCCCGGAGACGGTCACAGCTTGTCTGTAAGCGGATGCCGGGAGCAGACAAGCCCGTCAGGGCGCGTCAGCGGGTGTTGGCGGGTGTCGGGGCTGGCTTAACTATGCGGCATCAGAGCAGATTGTACTGAGAGTGCACCATATGCGGTGTGAAATACCGCACAGATGCGTAAGGAGAAAATACCGCATCAGGCGCCATTCGCCATTCAGGCTGCGCAACTGTTGGGAAGGGCGATCGGTGCGGGCCTCTTCGCTATTACGCCAGCTGGCGAAAGGGGGATGTGCTGCAAGGCGATTAAGTTGGGTAACGCCAGGGTTTTCCCAGTCACGACGTTGTAAAACGACGGCCAGTGCCAAGCTTGCATGCCTGCAGGTCGACGATTCATCGATGTGCAATAGGTCAATGTTAGGGTCGTATCCAGTGCGAATACCTCGGACCCTGCACAATCTCTAGAGGATCCCCGGGTACCGAGCTCGAATTCGTAATCATTTTCATGTCCC

NOVE05 5-CGCCAAAGGAGAATTGCCCTG-3

RT-primer：5-GTCGTATCCAGTGCAGGGTCCGAGGTATTCGCACTGGATACGACCAGGGC-3

Forward primer： 5-CATCGCGCCAAAGGAGAATT-3

Amplicon was verified by sequencing

CGGACATGACATGATTACGATTCGAGCTCGGTACCCGGGGATCCTCTAGAGATTCATCGCGCCAAAGGAGAATTGCCCTGGTCGTATCCAGTGCGAATACCTCGGACCCTGCACAATCGTCGACCTGCAGGCATGCAAGCTTGGCACTGGCCGTCGTTTTACAACGTCGTGACTGGGAAAACCCTGGCGTTACCCAACTTAATCGCCTTGCAGCACATCCCCCTTTCGCCAGCTGGCGTAATAGCGAAGAGGCCCGCACCGATCGCCCTTCCCAACAGTTGCGCAGCCTGAATGGCGAATGGCGCCTGATGCGGTATTTTCTCCTTACGCATCTGTGCGGTATTTCACACCGCATATGGTGCACTCTCAGTACAATCTGCTCTGATGCCGCATAGTTAAGCCAGCCCCGACACCCGCCAACACCCGCTGACGCGCCCTGACGGGCTTGTCTGCTCCCGGCATCCGCTTACAGACAAGCTGTGACCGTCTCCGGGAGCTGCATGTGTCAGAGGTTTTCACCGTCATCACCGAAACGCGCGAGACGAAAGGGCCTCGTGATACGCCTATTTTTATAGGTTAATGTCATGATAATAATGGTTTCTTAGACGTCAGGTGGCACTTTTCGGGGAAATGTGCGCGGAACCCCTATTTGTTTATTTTTCTAAATACATTCAAATATGTATCCGCTCATGAGACAATAACCCTGATAAATGCTTCAATAATATTGAAAAAGGAAGAGTATGAGTATTCAACATTTCCGTGTCGCCCTTATTCCCTTTTTTGCGGCATTTTGCCTTCCTGTTTTTGCTCACCCAGAAACGCTGGTGAAAGTAAAAGATGCTGAAGATCAGTTGGGTGCACGAGTGGGTTACATCGAACTGGATCTCAACAGCGGTAAGATCCTTGAGAGTTTTCGCCCCGAAGAACGTTTTCCAATGATGAGCACTTTTAAAGTTCTGCTATGTGGCGCGGTATTATCCCGTATTGACGCCCGGGCAAGAGCAACTCGGTCGCCGCATACACTATTCTCAGAATGACTTGGTTGAGTACTCCCCAGTCAAAAGAAAAGCATCTTACGGATGGCATGACAGTAAGAGAATTATGCAGTGCTGCATACCATGGAGTGATACCCTGGCGGCACTACTCTGACACGATCGGAGGACGAAGGAACTACGCTTTTTGCACAACTTGGGGATCATGTAACTCGCTCTGAATCGGTGGGAAACTCGGGACCTGTAATGGAAGGCCAATACTCTAA

NOVEL06 5-CATTCCTTGGATTTCCTGCATT-3

RT-primer：5-GTCGTATCCAGTGCAGGGTCCGAGGTATTCGCACTGGATACGACAATGCA-3

Forward primer： 5-CAGCGCATTCCTTGGATTTCC-3

Amplicon was verified by sequencing

TGACATGACATGATTACGATTCGAGCTCGGTACCCGGGGATCCTCTAGAGATTCAGCGCATTCCTTGGATTTCCTGCATTGTCGTATCCAGTGCGAATACCTCGGACCCTGCACTGGATACAATCGTCGACCTGCAGGCATGCAAGCTTGGCACTGGCCGTCGTTTTACAACGTCGTGACTGGGAAAACCCTGGCGTTACCCAACTTAATCGCCTTGCAGCACATCCCCCTTTCGCCAGCTGGCGTAATAGCGAAGAGGCCCGCACCGATCGCCCTTCCCAACAGTTGCGCAGCCTGAATGGCGAATGGCGCCTGATGCGGTATTTTCTCCTTACGCATCTGTGCGGTATTTCACACCGCATATGGTGCACTCTCAGTACAATCTGCTCTGATGCCGCATAGTTAAGCCAGCCCCGACACCCGCCAACACCCGCTGACGCGCCCTGACGGGCTTGTCTGCTCCCGGCATCCGCTTACAGACAAGCTGTGACCGTCTCCGGGAGCTGCATGTGTCAGAGGTTTTCACCGTCATCACCGAAACGCGCGAGACGAAAGGGCCTCGTGATACGCCTATTTTTATAGGTTAATGTCATGATAATAATGGTTTCTTAGACGTCAGGTGGCACTTTTCGGGGAAATGTGCGCGGAACCCCTATTTGTTTATTTTTCTAAATACATTCAAATATGTATCCGCTCATGAGACAATAACCCTGATAAATGCTTCAATAATATTGAAAAAGGAAGAGTATGAGTATTCAACATTTCCGTGTCGCCCTTATTCCCTTTTTTGCGGCATTTTGCCTTCCTGTTTTTGCTCACCCAGAAACGCTGGTGAAAGTAAAAGATGCTGAAGATCAGTTGGGTGCACGAGTGGGTTACATCGAACTGGATCTCAACAGCGGTAAGATCCTTGAGAGTTTTCGCCCCGAAGAACGTTTTCCAATGATGAGCACTTTTAAAGTTCTGCTATGTGGCGCGGTATTATCCCGTATTGACGCCGGCAAGAGCAACTCGGTCGCCGCATACACTATTCTCAGAATGACTTGGTTGAGTACTCACCAGTCACAGAAAAGCATCTTACGATGGCATGACAGTAGAGATTATGCAGTGCTGCATACATGAGTGAAAAACACTGCCGCACCTACTCTGACAACGATCGGAGGACGGAGGACTACCGCTTTTTTGCACAACATGGATCATGTTACTCGCCTTGATCGTGGAACGGGACCCTAAATGAAAGCCATACCTAC

NOVEL07 5-TCCTTGGGGTGATCTCGTAGT-3

RT-primer：5-GTCGTATCCAGTGCAGGGTCCGAGGTATTCGCACTGGATACGACACTACG-3

Forward primer： 5-CGGCGTCCTTGGGGTGATCT-3

Amplicon was verified by sequencing

AATGGAACATGATTACGATTCGAGCTCGGTACCCGGGGATCCTCTAGAGATTCGGCGTCCTTGGGGTGATCTCGTAGTGTCGTATCCAGTGCGAATACCTCGGACCCTGCACAATCGTCGACCTGCAGGCATGCAAGCTTGGCACTGGCCGTCGTTTTACAACGTCGTGACTGGGAAAACCCTGGCGTTACCCAACTTAATCGCCTTGCAGCACATCCCCCTTTCGCCAGCTGGCGTAATAGCGAAGAGGCCCGCACCGATCGCCCTTCCCAACAGTTGCGCAGCCTGAATGGCGAATGGCGCCTGATGCGGTATTTTCTCCTTACGCATCTGTGCGGTATTTCACACCGCATATGGTGCACTCTCAGTACAATCTGCTCTGATGCCGCATAGTTAAGCCAGCCCCGACACCCGCCAACACCCGCTGACGCGCCCTGACGGGCTTGTCTGCTCCCGGCATCCGCTTACAGACAAGCTGTGACCGTCTCCGGGAGCTGCATGTGTCAGAGGTTTTCACCGTCATCACCGAAACGCGCGAGACGAAAGGGCCTCGTGATACGCCTATTTTTATAGGTTAATGTCATGATAATAATGGTTTCTTAGACGTCAGGTGGCACTTTTCGGGGAAATGTGCGCGGAACCCCTATTTGTTTATTTTTCTAAATACATTCAAATATGTATCCGCTCATGAGACAATAACCCTGATAAATGCTTCAATAATATTGAAAAAGGAAGAGTATGAGTATTCAACATTTCCGTGTCGCCCTTATTCCCTTTTTTGCGGCATTTTGCCTTCCTGTTTTTGCTCACCCAGAAACGCTGGTGAAAGTAAAAGATGCTGAAGATCAGTTGGGTGCACGAGTGGGTTACATCGAACTGGATCTCAACAGCGGTAAGATCCTTGAGAGTTTTCGCCCCGAAGAACGTTTTCCAATGATGAGCACTTTTAAAGTTCTGCTATGTGGCGCGGTATTATCCCGTATTGACGCCCGGGCAGAGCAACTCGGTCGCCCGCATACACTATTCTCAGAATGACTTGGGTTGAGTACTCACCAGTCACAGAAAAGCATCTTACGGATGGCATGACAGTAGAGATTATGCAGTGCTGCATACAATGGAGTGATACACTGCGCAACCTACTCTGACACGAATCGGAGGAACGACGAGCCTACGGCTTTTTGGCACAACATGGGGATCTGTAACTCGCTGGATCGTGGACGGAGCTGAATGAGCCTATCCAAAAACGCACAG

NOVEL08 5-CTGGATGTAATTGTGGCACGG-3

RT-primer：5-GTCGTATCCAGTGCAGGGTCCGAGGTATTCGCACTGGATACGACCCGTGC-3

Forward primer： 5-CGCCGCTGGATGTAATTGTG-3

Amplicon was verified by sequencing

CAAAATGAACATGATTACGATTCGAGCTCGGTACCCGGGGATCCTCTAGAGATCGCCGCTGGATGTAATTGTGGCACGGGTCGTATCCAGTGCGAATACCTCGGACCCTGCACAATCGTCGACCTGCAGGCATGCAAGCTTGGCACTGGCCGTCGTTTTACAACGTCGTGACTGGGAAAACCCTGGCGTTACCCAACTTAATCGCCTTGCAGCACATCCCCCTTTCGCCAGCTGGCGTAATAGCGAAGAGGCCCGCACCGATCGCCCTTCCCAACAGTTGCGCAGCCTGAATGGCGAATGGCGCCTGATGCGGTATTTTCTCCTTACGCATCTGTGCGGTATTTCACACCGCATATGGTGCACTCTCAGTACAATCTGCTCTGATGCCGCATAGTTAAGCCAGCCCCGACACCCGCCAACACCCGCTGACGCGCCCTGACGGGCTTGTCTGCTCCCGGCATCCGCTTACAGACAAGCTGTGACCGTCTCCGGGAGCTGCATGTGTCAGAGGTTTTCACCGTCATCACCGAAACGCGCGAGACGAAAGGGCCTCGTGATACGCCTATTTTTATAGGTTAATGTCATGATAATAATGGTTTCTTAGACGTCAGGTGGCACTTTTCGGGGAAATGTGCGCGGAACCCCTATTTGTTTATTTTTCTAAATACATTCAAATATGTATCCGCTCATGAGACAATAACCCTGATAAATGCTTCAATAATATTGAAAAAGGAAGAGTATGAGTATTCAACATTTCCGTGTCGCCCTTATTCCCTTTTTTGCGGCATTTTGCCTTCCTGTTTTTGCTCACCCAGAAACGCTGGTGAAAGTAAAAGATGCTGAAGATCAGTTGGGTGCACGAGTGGGTTACATCGAACTGGATCTCAACAGCGGTAAGATCCTTGAGAGTTTTCGCCCCGAAGAACGTTTTCCAATGATGAGCACTTTTAAAGTTCTGCTATGTGGCGCGGTATTATCCCGTATTGACGCCGGGCAAGAGCAACTCGGTCGCCGCATACACTATTTCTCAGAATTGACTTGGTTTGAGTACCTCACCAGTCACAGGAAAAAGCATCTTACGATGGGCATGACAGTAGAGGAGTTAATGCAGGTGCCTGGCATTATCATGAATTGAAATAACACTGCGACCAACTACCTTCTGAACAACGGAATCGTGAGGATCCGGAATGAC

NOVEL09 5-TTTGTCCACGGATTGGGGCCA-3

RT-primer：5-GTCGTATCCAGTGCAGGGTCCGAGGTATTCGCACTGGATACGACTGGCCC-3

Forward primer： 5-CAGCGTTTGTCCACGGATTG-3

NOVEL10 5-CTTTCAGCAGCCTCCGGCGTC-3

RT-primer：5-GTCGTATCCAGTGCAGGGTCCGAGGTATTCGCACTGGATACGACGACGCC-3

Forward primer： 5-CAGCGCTTTCAGCAGCCTCC-3

Amplicon was verified by sequencing

ACACCGCTTCGTGTCGTTTGTATAGCTCATTCAGTTCCGTTCCCACGATCAGCGGTACATGATCCCCCATGTGTGCAAAAGCGTAGCCCTCGGTCTCGATCGTGTCAGAGTAAGTGCGCAGTGTTATCACTCATGATATGCAGCACTGCATAATCTCTACTGTCATGCCATCCGTAAGATGCTTTTCTGTGACTGGGGAGTACTCAACCAAGTCATTCTGAGAATAGTGTATGCGGGCGACCGAGTTGCTCTTGCCCGGCGTCAATACGGGATAATACCGCGCCACATAGCAGAACTTTAAAAGTGCTCATCATTGGAAAACGTTCTTCGGGGCGAAAACTCTCAAGGATCTTACCGCTGTTGAGATCCAGTTCGATGTAACCCACTCGTGCACCCAACTGATCTTCAGCATCTTTTACTTTCACCAGCGTTTCTGGGTGAGCAAAAACAGGAAGGCAAAATGCCGCAAAAAAGGGAATAAGGGCGACACGGAAATGTTGAATACTCATACTCTTCCTTTTTCAATATTATTGAAGCATTTATCAGGGTTATTGTCTCATGAGCGGATACATATTTGAATGTATTTAGAAAAATAAACAAATAGGGGTTCCGCGCACATTTCCCCGAAAAGTGCCACCTGACGTCTAAGAAACCATTATTATCATGACATTAACCTATAAAAATAGGCGTATCACGAGGCCCTTTCGTCTCGCGCGTTTCGGTGATGACGGTGAAAACCTCTGACACATGCAGCTCCCGGAGACGGTCACAGCTTGTCTGTAAGCGGATGCCGGGAGCAGACAAGCCCGTCAGGGCGCGTCAGCGGGTGTTGGCGGGTGTCGGGGCTGGCTTAACTATGCGGCATCAGAGCAGATTGTACTGAGAGTGCACCATATGCGGTGTGAAATACCGCACAGATGCGTAAGGAGAAAATACCGCATCAGGCGCCATTCGCCATTCAGGCTGCGCAACTGTTGGGAAGGGCGATCGGTGCGGGCCTCTTCGCTATTACGCCAGCTGGCGAAAGGGGGATGTGCTGCAAGGCGATTAAGTTGGGTAACGCCAGGGTTTTCCCAGTCACGACGTTGTAAAACGACGGCCAGTGCCAAGCTTGCATGCCTGCAGGTCGACGATTCAGCGCTTTCAGCAGCCTCCGGCGTCGTCGTATCCAGTGCGAATACCTCGGACCCTGCACAATCTCTAGAGGATCCCCGGGTACCGAGCTCGAATCGTAATCATGTCATTGGTCCT

NOVEL11 5-GTGGAAGTAGCAAAGAAAAGC-3

RT-primer：5-GTCGTATCCAGTGCAGGGTCCGAGGTATTCGCACTGGATACGACGCTTTT-3

Forward primer： 5-CCGAGGTGGAAGTAGCAAAG-3

Amplicon was verified by sequencing

AATTGTACATGCATTACGATTCGAGCTCGGTACCCGGGGATCCTCTAGAGATTCCGAGGTGGAAGTAGCAAAGGAAAAAGCGTCGTATCCAGTGCGAATACCTCGGACCCTGCACAATCGTCGACCTGCAGGCATGCAAGCTTGGCACTGGCCGTCGTTTTACAACGTCGTGACTGGGAAAACCCTGGCGTTACCCAACTTAATCGCCTTGCAGCACATCCCCCTTTCGCCAGCTGGCGTAATAGCGAAGAGGCCCGCACCGATCGCCCTTCCCAACAGTTGCGCAGCCTGAATGGCGAATGGCGCCTGATGCGGTATTTTCTCCTTACGCATCTGTGCGGTATTTCACACCGCATATGGTGCACTCTCAGTACAATCTGCTCTGATGCCGCATAGTTAAGCCAGCCCCGACACCCGCCAACACCCGCTGACGCGCCCTGACGGGCTTGTCTGCTCCCGGCATCCGCTTACAGACAAGCTGTGACCGTCTCCGGGAGCTGCATGTGTCAGAGGTTTTCACCGTCATCACCGAAACGCGCGAGACGAAAGGGCCTCGTGATACGCCTATTTTTATAGGTTAATGTCATGATAATAATGGTTTCTTAGACGTCAGGTGGCACTTTTCGGGGAAATGTGCGCGGAACCCCTATTTGTTTATTTTTCTAAATACATTCAAATATGTATCCGCTCATGAGACAATAACCCTGATAAATGCTTCAATAATATTGAAAAAGGAAGAGTATGAGTATTCAACATTTCCGTGTCGCCCTTATTCCCTTTTTTGCGGCATTTTGCCTTCCTGTTTTTGCTCACCCAGAAACGCTGGTGAAAGTAAAAGATGCTGAAGATCAGTTGGGTGCACGAGTGGGTTACATCGAACTGGATCTCAACAGCGGTAAGATCCTTGAGAGTTTTCGCCCCGAAGAACGTTTTCCAATGATGAGCACTTTTAAAGTTCTGCTATGTGGCGCGGTATTATCCCGTATTGACGCCGGGCAGAGCAACTCGGTCGCGCATACACTATTCTCAGATGACTTGTGAGTACTCCACCAGTCACAGAAAAAGCATCTTACGATGCATGGACAGTAGAGATTATGCATGCTGCATACATGATGATACACTGCCGCACCTACTCTGACACGATCGAGACGGAGGACTACCGCTTTTGCCAAATTGGGAATCTGTAACTCGCCTTGAATCGATGGATCGGGACTGATGAGCCATACA

NOVEL12 5-TTTTGTTGCATGATGCTGATAA-3

RT-primer：5-GTCGTATCCAGTGCAGGGTCCGAGGTATTCGCACTGGATACGACTTATCA-3

Forward primer： 5-CAGCCTTTTGTTGCATGATGC-3

Amplicon was verified by sequencing

CAAAATGAACATGATTACGATTCGAGCTCGGTACCCGGGGATCCTCTAGAGATTCAGCCTTTTGTTGCATGATGCTGATAAGTCGTATCCAGTGCGAATACCTCGGACCCTGCACAATCGTCGACCTGCAGGCATGCAAGCTTGGCACTGGCCGTCGTTTTACAACGTCGTGACTGGGAAAACCCTGGCGTTACCCAACTTAATCGCCTTGCAGCACATCCCCCTTTCGCCAGCTGGCGTAATAGCGAAGAGGCCCGCACCGATCGCCCTTCCCAACAGTTGCGCAGCCTGAATGGCGAATGGCGCCTGATGCGGTATTTTCTCCTTACGCATCTGTGCGGTATTTCACACCGCATATGGTGCACTCTCAGTACAATCTGCTCTGATGCCGCATAGTTAAGCCAGCCCCGACACCCGCCAACACCCGCTGACGCGCCCTGACGGGCTTGTCTGCTCCCGGCATCCGCTTACAGACAAGCTGTGACCGTCTCCGGGAGCTGCATGTGTCAGAGGTTTTCACCGTCATCACCGAAACGCGCGAGACGAAAGGGCCTCGTGATACGCCTATTTTTATAGGTTAATGTCATGATAATAATGGTTTCTTAGACGTCAGGTGGCACTTTTCGGGGAAATGTGCGCGGAACCCCTATTTGTTTATTTTTCTAAATACATTCAAATATGTATCCGCTCATGAGACAATAACCCTGATAAATGCTTCAATAATATTGAAAAAGGAAGAGTATGAGTATTCAACATTTCCGTGTCGCCCTTATTCCCTTTTTTGCGGCATTTTGCCTTCCTGTTTTTGCTCACCCAGAAACGCTGGTGAAAGTAAAAGATGCTGAAGATCAGTTGGGTGCACGAGTGGGTTACATCGAACTGGATCTCAACAGCGGTAAGATCCTTGAGAGTTTTCGCCCCGAAGAACGTTTTCCAATGATGAGCACTTTTAAAGTTCTGCTATGTGGCGCGGTATTATCCCGTATTGACGCCGGGCAAGAGCAACTCGGTCGCCGCATACACTATTTCTCAGAATTGACTTGGTTTGAGTACCTCACCAGTCACAGGAAAAAGCATCTTACGATGGGCATGACAGTAGAGGAGTTAATGCAGGTGCCTGGCATTATCATGAATTGAAATAACACTGCGACCAACTACCTTCTGAACAACGGAATCGTGAGGATCCGGAATGAC

NOVEL13 5-GCAAGTCGTCTTTGGCTATTT-3

RT-primer：5-GTCGTATCCAGTGCAGGGTCCGAGGTATTCGCACTGGATACGACAAATAG-3

Forward primer： 5-CGGCGGCAAGTCGTCTTTGG-3

Amplicon was verified by sequencing

GTACACACGCGCTCTCGGTCGTTGGTATTGTCTTCATCAGGTCCGGTACCAACGATCAAGCGAGTACTGATTCCCCATGTTGTGCAAAAAGCCGTAGCTCCTCGTCTCGATCGTGTCAGGAGTAAGTGCGCAGTGTATCATCATTGTATGCAGCACTGCATAATTCTCTACTGTCATGCCATCCGTAAGATGCTTTTCTGTGACTGGTGGAGTACTCAAACCAAGTCATTCTGAGAATAGTGTATGCGGCGACCGAGTTGCTCTTGCCCGGCGTCAATACGGGATAATAACCGCGCCACATAGCAGAACTTTAAAAGTGCTCATCATTGGAAAACGTTCTTCGGGGCGAAAACTCTCAAGGATCTTACCGCTGTTGAGATCCAGTTCGATGTAACCCACTCGTGCACCCAACTGATCTTCAGCATCTTTTACTTTCACCAGCGTTTCTGGGTGAGCAAAAACAGGAAGGCAAAATGCCGCAAAAAAGGGAATAAGGGCGACACGGAAATGTTGAATACTCATACTCTTCCTTTTTCAATATTATTGAAGCATTTATCAGGGTTATTGTCTCATGAGCGGATACATATTTGAATGTATTTAGAAAAATAAACAAATAGGGGTTCCGCGCACATTTCCCCGAAAAGTGCCACCTGACGTCTAAGAAACCATTATTATCATGACATTAACCTATAAAAATAGGCGTATCACGAGGCCCTTTCGTCTCGCGCGTTTCGGTGATGACGGTGAAAACCTCTGACACATGCAGCTCCCGGAGACGGTCACAGCTTGTCTGTAAGCGGATGCCGGGAGCAGACAAGCCCGTCAGGGCGCGTCAGCGGGTGTTGGCGGGTGTCGGGGCTGGCTTAACTATGCGGCATCAGAGCAGATTGTACTGAGAGTGCACCATATGCGGTGTGAAATACCGCACAGATGCGTAAGGAGAAAATACCGCATCAGGCGCCATTCGCCATTCAGGCTGCGCAACTGTTGGGAAGGGCGATCGGTGCGGGCCTCTTCGCTATTACGCCAGCTGGCGAAAGGGGGATGTGCTGCAAGGCGATTAAGTTGGGTAACGCCAGGGTTTTCCCAGTCACGACGTTGTAAAACGACGGCCAGTGCCAAGCTTGCATGCCTGCAGGTCGACGATTCGGCGGCAAGTCGTCTTTGGCTATTTGTCGTATCCAGTGCGAATACCTCGGACCCTGCACAATCTCTAGAGGATCCCCGGGTACCGAGCTCGAATCGTAATCATGTCCAATG

NOVEL14 5-GCAAGTCGTCTTTGGCTATT-3

RT-primer：5-GTCGTATCCAGTGCAGGGTCCGAGGTATTCGCACTGGATACGACAATAGC-3

Forward primer： 5-CAGCGGCAAGTCGTCTTTG-3

Amplicon was verified by sequencing

CTTCGTGCGGTTTGGATATAGACTTCACATCAGGTCCCGATTCCAACGATTCAAGCGAGTTACGATCCCCATGTTGGCCAAAAAGCGTAAGCTTCTCGTTCTTCGATCGTGTCAGAGTAGTTGCGCAGTGTATCATCATGTATGCAGCACTGCATATCTCTACTGTCATGCCATCCGTAAGATGCTTTTCTGTGACTGGTGAGTACTCAACCAAGTCATTCTGAGAATAGTGTATGCGGCGACCGAGTTGCTCTTGCCCGGCGTCAATACGGGATAATACCGCGCCACATAGCAGAACTTTAAAAGTGCTCATCATTGGAAAACGTTCTTCGGGGCGAAAACTCTCAAGGATCTTACCGCTGTTGAGATCCAGTTCGATGTAACCCACTCGTGCACCCAACTGATCTTCAGCATCTTTTACTTTCACCAGCGTTTCTGGGTGAGCAAAAACAGGAAGGCAAAATGCCGCAAAAAAGGGAATAAGGGCGACACGGAAATGTTGAATACTCATACTCTTCCTTTTTCAATATTATTGAAGCATTTATCAGGGTTATTGTCTCATGAGCGGATACATATTTGAATGTATTTAGAAAAATAAACAAATAGGGGTTCCGCGCACATTTCCCCGAAAAGTGCCACCTGACGTCTAAGAAACCATTATTATCATGACATTAACCTATAAAAATAGGCGTATCACGAGGCCCTTTCGTCTCGCGCGTTTCGGTGATGACGGTGAAAACCTCTGACACATGCAGCTCCCGGAGACGGTCACAGCTTGTCTGTAAGCGGATGCCGGGAGCAGACAAGCCCGTCAGGGCGCGTCAGCGGGTGTTGGCGGGTGTCGGGGCTGGCTTAACTATGCGGCATCAGAGCAGATTGTACTGAGAGTGCACCATATGCGGTGTGAAATACCGCACAGATGCGTAAGGAGAAAATACCGCATCAGGCGCCATTCGCCATTCAGGCTGCGCAACTGTTGGGAAGGGCGATCGGTGCGGGCCTCTTCGCTATTACGCCAGCTGGCGAAAGGGGGATGTGCTGCAAGGCGATTAAGTTGGGTAACGCCAGGGTTTTCCCAGTCACGACGTTGTAAAACGACGGCCAGTGCCAAGCTTGCATGCCTGCAGGTCGACGATTCAGCGGCAAGTCGTCTTTGGCTATTGTCGTATCCAGTGCGAATACCTCGGACCCTGCACAATCTCTAGAGGATCCCCGGGTACCGAGCTCGAATTCGTAAATCATTGTTCAATGTA

NOVEL15 5-TGCAACTGTGGTACGGTACCA-3

RT-primer：5-GTCGTATCCAGTGCAGGGTCCGAGGTATTCGCACTGGATACGACTGGTAC-3

Forward primer： 5-CAGCGTGCAACTGTGGTACG-3

Amplicon was verified by sequencing

AATTGGACCTGATTACGATTCGAGCTCGGTACCCGGGGATCCTCTAGAGATTCAGCGTGCAACTGTGGTACGGTACCAGTCGTACCAGTGCGAATACCTCGGACCCTGCACAATCGTCGACCTGCAGGCATGCAAGCTTGGCACTGGCCGTCGTTTTACAACGTCGTGACTGGGAAAACCCTGGCGTTACCCAACTTAATCGCCTTGCAGCACATCCCCCTTTCGCCAGCTGGCGTAATAGCGAAGAGGCCCGCACCGATCGCCCTTCCCAACAGTTGCGCAGCCTGAATGGCGAATGGCGCCTGATGCGGTATTTTCTCCTTACGCATCTGTGCGGTATTTCACACCGCATATGGTGCACTCTCAGTACAATCTGCTCTGATGCCGCATAGTTAAGCCAGCCCCGACACCCGCCAACACCCGCTGACGCGCCCTGACGGGCTTGTCTGCTCCCGGCATCCGCTTACAGACAAGCTGTGACCGTCTCCGGGAGCTGCATGTGTCAGAGGTTTTCACCGTCATCACCGAAACGCGCGAGACGAAAGGGCCTCGTGATACGCCTATTTTTATAGGTTAATGTCATGATAATAATGGTTTCTTAGACGTCAGGTGGCACTTTTCGGGGAAATGTGCGCGGAACCCCTATTTGTTTATTTTTCTAAATACATTCAAATATGTATCCGCTCATGAGACAATAACCCTGATAAATGCTTCAATAATATTGAAAAAGGAAGAGTATGAGTATTCAACATTTCCGTGTCGCCCTTATTCCCTTTTTTGCGGCATTTTGCCTTCCTGTTTTTGCTCACCCAGAAACGCTGGTGAAAGTAAAAGATGCTGAAGATCAGTTGGGTGCACGAGTGGGTTACATCGAACTGGATCTCAACAGCGGTAAGATCCTTGAGAGTTTTCGCCCCGAAGAACGTTTTCCAATGATGAGCACTTTTAAAGTTCTGCTATGTGGCGCGGTATTATCCCGTATTGACGCCCGGGCAAGAGCAACTCGGTCGCCGCATACACTATTCTCAGAATGACTTGGTTGAGTACTCACCAGTCACAGAAAAGCATCTTACGATGGCATGACAGTAGAGAATTATGCAGTGCTGCATACATGATGATACACTGCGCACTTACCTCTGACACGATCGAGAACGAGGAGCTACGCTTTTTGCCAACATGGGGGAATCGGTACTCGCTTGATCGTTGGTACGACTTGAATTGAAGCCATATACTAACGACGGAGCCGGTTACCAT

NOVEL16 5-AGCAAGCATCCTGGGCTAAT-3

RT-primer：5-GTCGTATCCAGTGCAGGGTCCGAGGTATTCGCACTGGATACGACATTAGC-3

Forward primer： 5-CGCCGAGCAAGCATCCTGG-3

Amplicon was verified by sequencing

TAATGGGACCTTGATTACGATTCGAGCTCGGTACCCGGGGATCCTCTAGAGATTCGCCGAGCAAGCATCCTGGGCTAATGTCGTATCCAGTGCGAATACCTCGGACCCTGCACAATCGTCGACCTGCAGGCATGCAAGCTTGGCACTGGCCGTCGTTTTACAACGTCGTGACTGGGAAAACCCTGGCGTTACCCAACTTAATCGCCTTGCAGCACATCCCCCTTTCGCCAGCTGGCGTAATAGCGAAGAGGCCCGCACCGATCGCCCTTCCCAACAGTTGCGCAGCCTGAATGGCGAATGGCGCCTGATGCGGTATTTTCTCCTTACGCATCTGTGCGGTATTTCACACCGCATATGGTGCACTCTCAGTACAATCTGCTCTGATGCCGCATAGTTAAGCCAGCCCCGACACCCGCCAACACCCGCTGACGCGCCCTGACGGGCTTGTCTGCTCCCGGCATCCGCTTACAGACAAGCTGTGACCGTCTCCGGGAGCTGCATGTGTCAGAGGTTTTCACCGTCATCACCGAAACGCGCGAGACGAAAGGGCCTCGTGATACGCCTATTTTTATAGGTTAATGTCATGATAATAATGGTTTCTTAGACGTCAGGTGGCACTTTTCGGGGAAATGTGCGCGGAACCCCTATTTGTTTATTTTTCTAAATACATTCAAATATGTATCCGCTCATGAGACAATAACCCTGATAAATGCTTCAATAATATTGAAAAAGGAAGAGTATGAGTATTCAACATTTCCGTGTCGCCCTTATTCCCTTTTTTGCGGCATTTTGCCTTCCTGTTTTTGCTCACCCAGAAACGCTGGTGAAAGTAAAAGATGCTGAAGATCAGTTGGGTGCACGAGTGGGTTACATCGAACTGGATCTCAACAGCGGTAAGATCCTTGAGAGTTTTCGCCCCGAAGAACGTTTTCCAATGATGAGCACTTTTAAAGTTCTGCTATGTGGCGCGGTATTATCCCGTATTGACGCCCGGGCAAGAGCAACTCGGTCGCCGCATACACTATTCCTCAGAATGACTTGGTTGAGTACTCACCAGTCACAGAAAAGCATCTACGGATGGCATGACAGTAGAGATTATGCAGTGCTGCAATACCATGATGATACCACTGCGGCACTTACCTTCTGACACGATCGAGACGAGGACTAACGGCTTTTGCACAACATGGGGGATCTGTACTCGCTGATCGTGGTCGGACTGAATGAGCCATACCAACGACACGAAGGCGGTTGTAC

NOVEL17 5-TGTGTATGTTACGTTCAACGT-3

RT-primer：5-GTCGTATCCAGTGCAGGGTCCGAGGTATTCGCACTGGATACGACACGTTG-3

Forward primer： 5-CAGCCTGTGTATGTTACGTT-3

Amplicon was verified by sequencing

CAAACCATATGACATGATTACGATTCGAGCTCGGTACCCGGGGATCCTCTAGAGATCAGCCTGTGTATGTTACGTTCAACGTGTCGTATCCAGTGCGCATACCTCGGACCCTGCACAATCGTCGACCTGCAGGCATGCAAGCTTGGCACTGGCCGTCGTTTTACAACGTCGTGACTGGGAAAACCCTGGCGTTACCCAACTTAATCGCCTTGCAGCACATCCCCCTTTCGCCAGCTGGCGTAATAGCGAAGAGGCCCGCACCGATCGCCCTTCCCAACAGTTGCGCAGCCTGAATGGCGAATGGCGCCTGATGCGGTATTTTCTCCTTACGCATCTGTGCGGTATTTCACACCGCATATGGTGCACTCTCAGTACAATCTGCTCTGATGCCGCATAGTTAAGCCAGCCCCGACACCCGCCAACACCCGCTGACGCGCCCTGACGGGCTTGTCTGCTCCCGGCATCCGCTTACAGACAAGCTGTGACCGTCTCCGGGAGCTGCATGTGTCAGAGGTTTTCACCGTCATCACCGAAACGCGCGAGACGAAAGGGCCTCGTGATACGCCTATTTTTATAGGTTAATGTCATGATAATAATGGTTTCTTAGACGTCAGGTGGCACTTTTCGGGGAAATGTGCGCGGAACCCCTATTTGTTTATTTTTCTAAATACATTCAAATATGTATCCGCTCATGAGACAATAACCCTGATAAATGCTTCAATAATATTGAAAAAGGAAGAGTATGAGTATTCAACATTTCCGTGTCGCCCTTATTCCCTTTTTTGCGGCATTTTGCCTTCCTGTTTTTGCTCACCCAGAAACGCTGGTGAAAGTAAAAGATGCTGAAGATCAGTTGGGTGCACGAGTGGGTTACATCGAACTGGATCTCAACAGCGGTAAGATCCCTTGAGAGTTTTCGCCCCGAAGAACGTTTTCCAATGATGAGCACTTTTAAAGTTCTGCTATGTGGCGCGGTATTATCCCGTATTGACGCCCGGGCAAGAGCAACTCGGTCGCCGCATACACTAATTCTCAGAATGACTTGGTTGAGTACTCCACCAGTCACAGAAAAGCATCTTACGGATGGCATGACAGTAGAGAATTATGCAGTGCTGCAATAACATGATGATAACACTGCGCAACTACCTCTGACACGATCGGAGGAACGGATGACCTAACCGGCTTTTTGCACAACTTGGATCGTAACTCGCCTGATCGTGGGAACCGACTAGTATGTGAGCCATATCACACGTGA

NOVEL18 5-AGGCAGTCTCCTTGGCTAAG-3

RT-primer：5-GTCGTATCCAGTGCAGGGTCCGAGGTATTCGCACTGGATACGACCTTAGC-3

Forward primer： 5-CATCGAGGCAGTCTCCTTG-3

Amplicon was verified by sequencing

CATACTAAGGCGGTTAAGGTCCATCCGGTCCTCCCGATCGTGTCCGAGTAAGTTTGGACCGGCAGGTGTTATCATTCATGGGTAATGCAGCATGGCATAAATCTCTAACTGGGTCATGGCCATTCCGTAAGGATGCTTTTTCTTGAGAACTGGTGGAGTACTCCAACCAAAGTCAATTCTGAGAATAGGTGTATGCGGCGGACCCGAGTTGCTCTTGCCCGGCGTCAATACGGGAATAATACCGCGGCCACATAGCAGAACTTTAAAAAGTGCTCATCATTGGAAAACGTTCTTCGGGGGCGAAAAACTCTCAAGGATCTTACCGCTGTTGAGATCCAGTTCGATGTAACCCACTCGTGCACCCAACTGATCTTCAGCATCTTTTACTTTCACCAGCGTTTCTGGGTGAGCAAAAACAGGAAGGCAAAATGCCGCAAAAAAGGGAATAAGGGCGACACGGAAATGTTGAATACTCATACTCTTCCTTTTTCAATATTATTGAAGCATTTATCAGGGTTATTGTCTCATGAGCGGATACATATTTGAATGTATTTAGAAAAATAAACAAATAGGGGTTCCGCGCACATTTCCCCGAAAAGTGCCACCTGACGTCTAAGAAACCATTATTATCATGACATTAACCTATAAAAATAGGCGTATCACGAGGCCCTTTCGTCTCGCGCGTTTCGGTGATGACGGTGAAAACCTCTGACACATGCAGCTCCCGGAGACGGTCACAGCTTGTCTGTAAGCGGATGCCGGGAGCAGACAAGCCCGTCAGGGCGCGTCAGCGGGTGTTGGCGGGTGTCGGGGCTGGCTTAACTATGCGGCATCAGAGCAGATTGTACTGAGAGTGCACCATATGCGGTGTGAAATACCGCACAGATGCGTAAGGAGAAAATACCGCATCAGGCGCCATTCGCCATTCAGGCTGCGCAACTGTTGGGAAGGGCGATCGGTGCGGGCCTCTTCGCTATTACGCCAGCTGGCGAAAGGGGGATGTGCTGCAAGGCGATTAAGTTGGGTAACGCCAGGGTTTTCCCAGTCACGACGTTGTAAAACGACGGCCAGTGCCAAGCTTGCATGCCTGCAGGTCGACGATTCATCGAGGCAGTCTCCTTGGCTAAGGTCGTATCCAGTGCGAATACCTCGGACCCTGCACAATCTCTAGAGGATCCCCGGGTACCGAGCTCGAATTCGTAATCAGGGTCCATG

NOVEL19 5-TAGCCAAGGATGACTTGCCT-3

RT-primer：5-GTCGTATCCAGTGCAGGGTCCGAGGTATTCGCACTGGATACGACAGGCAA-3

Forward primer： 5-CATCGTAGCCAAGGATGAC-3

Amplicon was verified by sequencing

GGGGAACCTTTGATTTACGATTCGAGCTCGGTACCCGGGGATCCTCTAGAGATTCATCGTAGCCAAGGATGACTTGCCTGTCGTATCCAGTGCGAATACCTCGGACCCTGCACAATCGTCGACCTGCAGGCATGCAAGCTTGGCACTGGCCGTCGTTTTACAACGTCGTGACTGGGAAAACCCTGGCGTTACCCAACTTAATCGCCTTGCAGCACATCCCCCTTTCGCCAGCTGGCGTAATAGCGAAGAGGCCCGCACCGATCGCCCTTCCCAACAGTTGCGCAGCCTGAATGGCGAATGGCGCCTGATGCGGTATTTTCTCCTTACGCATCTGTGCGGTATTTCACACCGCATATGGTGCACTCTCAGTACAATCTGCTCTGATGCCGCATAGTTAAGCCAGCCCCGACACCCGCCAACACCCGCTGACGCGCCCTGACGGGCTTGTCTGCTCCCGGCATCCGCTTACAGACAAGCTGTGACCGTCTCCGGGAGCTGCATGTGTCAGAGGTTTTCACCGTCATCACCGAAACGCGCGAGACGAAAGGGCCTCGTGATACGCCTATTTTTATAGGTTAATGTCATGATAATAATGGTTTCTTAGACGTCAGGTGGCACTTTTCGGGGAAATGTGCGCGGAACCCCTATTTGTTTATTTTTCTAAATACATTCAAATATGTATCCGCTCATGAGACAATAACCCTGATAAATGCTTCAATAATATTGAAAAAGGAAGAGTATGAGTATTCAACATTTCCGTGTCGCCCTTATTCCCTTTTTTGCGGCATTTTGCCTTCCTGTTTTTGCTCACCCAGAAACGCTGGTGAAAGTAAAAGATGCTGAAGATCAGTTGGGTGCACGAGTGGGTTACATCGAACTGGATCTCAACAGCGGTAAGATCCTTGAGAGTTTTCGCCCCGAAGAACGTTTTCCAATGATGAGCACTTTTAAAGTTCTGCTATGTGGCGCGGTATTATCCCGTATTGACGCCGGGCAAGAGCAACTCGGGTCGCCGCATACCACTATTCTCAGATGACTTGGTTGAGTACTCCACAGTCACAGAAAAAGCATCTTACGGATGGCATGACAGTAAGAGAATTATGCATGCTGCATACATGGATGAATAACACTGCCGACACCTACCTCTGACACGATCGAGACGAGGAACTACGCTTTTTGCCAACATGGGATCAGTACCTCGCCCTGAATCGTGGCACGGAGCTTGAATGTAAGTCCT

NOVEL20 5-GGACGCTCAGGATTGCGCCATGT-3

RT-primer：5-GTCGTATCCAGTGCAGGGTCCGAGGTATTCGCACTGGATACGACACATGG-3

Forward primer： 5-CAGCGGGACGCTCAGGATTGCG-3

Amplicon was verified by sequencing

CAAACCATATGACATGATTACGATTCGAGCTCGGTACCCGGGGATCCTCTAGAGATTCAGCGGGACGCTCAGGATTGCGCCATGTGTCGTATCCAGTGCGCATACCTCGGACCCTGCACAATCGTCGACCTGCAGGCATGCAAGCTTGGCACTGGCCGTCGTTTTACAACGTCGTGACTGGGAAAACCCTGGCGTTACCCAACTTAATCGCCTTGCAGCACATCCCCCTTTCGCCAGCTGGCGTAATAGCGAAGAGGCCCGCACCGATCGCCCTTCCCAACAGTTGCGCAGCCTGAATGGCGAATGGCGCCTGATGCGGTATTTTCTCCTTACGCATCTGTGCGGTATTTCACACCGCATATGGTGCACTCTCAGTACAATCTGCTCTGATGCCGCATAGTTAAGCCAGCCCCGACACCCGCCAACACCCGCTGACGCGCCCTGACGGGCTTGTCTGCTCCCGGCATCCGCTTACAGACAAGCTGTGACCGTCTCCGGGAGCTGCATGTGTCAGAGGTTTTCACCGTCATCACCGAAACGCGCGAGACGAAAGGGCCTCGTGATACGCCTATTTTTATAGGTTAATGTCATGATAATAATGGTTTCTTAGACGTCAGGTGGCACTTTTCGGGGAAATGTGCGCGGAACCCCTATTTGTTTATTTTTCTAAATACATTCAAATATGTATCCGCTCATGAGACAATAACCCTGATAAATGCTTCAATAATATTGAAAAAGGAAGAGTATGAGTATTCAACATTTCCGTGTCGCCCTTATTCCCTTTTTTGCGGCATTTTGCCTTCCTGTTTTTGCTCACCCAGAAACGCTGGTGAAAGTAAAAGATGCTGAAGATCAGTTGGGTGCACGAGTGGGTTACATCGAACTGGATCTCAACAGCGGTAAGATCCCTTGAGAGTTTTCGCCCCGAAGAACGTTTTCCAATGATGAGCACTTTTAAAGTTCTGCTATGTGGCGCGGTATTATCCCGTATTGACGCCCGGGCAAGAGCAACTCGGTCGCCGCATACACTAATTCTCAGAATGACTTGGTTGAGTACTCCACCAGTCACAGAAAAGCATCTTACGGATGGCATGACAGTAGAGAATTATGCAGTGCTGCAATAACATGATGATAACACTGCGCAACTACCTCTGACACGATCGGAGGAACGGATGACCTAACCGGCTTTTTGCACAACTTGGATCGTAACTCGCCTGATCGTGGGAACCGACTAGTATGTGAGCCATATCACACGTGA

NOVEL21 5-TATGTTGCAACTGTGGTATGGTA-3

RT-primer：5-GTCGTATCCAGTGCAGGGTCCGAGGTATTCGCACTGGATACGACTACCAT-3

Forward primer： 5-CGGCGTATGTTGCAACTGTGGT-3

Amplicon was verified by sequencing

ATGAACTTTCGAGCTCGGTACCCGGGGATCCTCTAGAGATTCGGCGTATGTTGCAACTGTGGTATGGTAGTCGTATCCAGTGCGAATACCTCGGACCCTGCACAATCGTCGACCTGCAGGCATGCAAGCTTGGCACTGGCCGTCGTTTTACAACGTCGTGACTGGGAAAACCCTGGCGTTACCCAACTTAATCGCCTTGCAGCACATCCCCCTTTCGCCAGCTGGCGTAATAGCGAAGAGGCCCGCACCGATCGCCCTTCCCAACAGTTGCGCAGCCTGAATGGCGAATGGCGCCTGATGCGGTATTTTCTCCTTACGCATCTGTGCGGTATTTCACACCGCATATGGTGCACTCTCAGTACAATCTGCTCTGATGCCGCATAGTTAAGCCAGCCCCGACACCCGCCAACACCCGCTGACGCGCCCTGACGGGCTTGTCTGCTCCCGGCATCCGCTTACAGACAAGCTGTGACCGTCTCCGGGAGCTGCATGTGTCAGAGGTTTTCACCGTCATCACCGAAACGCGCGAGACGAAAGGGCCTCGTGATACGCCTATTTTTATAGGTTAATGTCATGATAATAATGGTTTCTTAGACGTCAGGTGGCACTTTTCGGGGAAATGTGCGCGGAACCCCTATTTGTTTATTTTTCTAAATACATTCAAATATGTATCCGCTCATGAGACAATAACCCTGATAAATGCTTCAATAATATTGAAAAAGGAAGAGTATGAGTATTCAACATTTCCGTGTCGCCCTTATTCCCTTTTTTGCGGCATTTTGCCTTCCTGTTTTTGCTCACCCAGAAACGCTGGTGAAAGTAAAAGATGCTGAAGATCAGTTGGGTGCACGAGTGGGTTACATCGAACTGGATCTCAACAGCGGTAAGATCCTTGAGAGTTTTCGCCCCGAAGAACGTTTTCCAATGATGAGCACTTTTAAAGTTCTGCTATGTGGCGCGGTATTATCCCGTATTGACGCCCGGGCAAGAGCAACTCGGTCGCCGCATACACTATTCTCAGAATTGACTTGGTTGAGTACTCACCAGTCACAGAAAAGCATCTTACGGATGGCATGACAGTAGAAGAATTATGCAGTGCTGCCAAACCATGATGATACACTGGCGGCCACTACTTCTGACAACGATCGGGAGGACGAGGAGCTAACCGCTTTTGCACCACACATGGGGGATCTATGTTAACTCGCCTGATCGTGGGATCCCGGACTGTAATGGAAGCCCTTACCCAACGCACCAGAGCGCGGTGGTAAAATAG

NOVEL22 5-TGTGGCAGCATATCAGTGGACGA-3

RT-primer：5-GTCGTATCCAGTGCAGGGTCCGAGGTATTCGCACTGGATACGACTCGTCC-3

Forward primer： 5-CAGCGTGTGGCAGCATATCAGT-3

Amplicon was verified by sequencing

CTTATGCTACACCGAGGACATCTCGTGGTGTGTCGAACCGCTCGGTCGTGTAGCTTCATTCAGGTTCCGGTTCTATCGATCCAGGGCGGAGGTAAATGATCCCCCATGTTTGTCAAAAAGCGGTAGCTCCTTCGTTCTCGGATTCGTGTCAGAAGTAAGTGCCGCAGTGTTATCACTCATGGTTATGGCAGCACTGCATAATCTCTACTGTCATGCCATCCGTAAGATGCTTTTCTGTGACTGTGAGTACTCAACCAAGTCATTCTGAGAATAGTGTATGCGGCGACCGAGTTGCTCTTGCCCGGCGTCAATACGGGATAATACCGCGCCACATAGCAGAACTTTAAAAGTGCTCATCATTGGAAAACGTTCTTCGGGGCGAAAACTCTCAAGGATCTTACCGCTGTTGAGATCCAGTTCGATGTAACCCACTCGTGCACCCAACTGATCTTCAGCATCTTTTACTTTCACCAGCGTTTCTGGGTGAGCAAAAACAGGAAGGCAAAATGCCGCAAAAAAGGGAATAAGGGCGACACGGAAATGTTGAATACTCATACTCTTCCTTTTTCAATATTATTGAAGCATTTATCAGGGTTATTGTCTCATGAGCGGATACATATTTGAATGTATTTAGAAAAATAAACAAATAGGGGTTCCGCGCACATTTCCCCGAAAAGTGCCACCTGACGTCTAAGAAACCATTATTATCATGACATTAACCTATAAAAATAGGCGTATCACGAGGCCCTTTCGTCTCGCGCGTTTCGGTGATGACGGTGAAAACCTCTGACACATGCAGCTCCCGGAGACGGTCACAGCTTGTCTGTAAGCGGATGCCGGGAGCAGACAAGCCCGTCAGGGCGCGTCAGCGGGTGTTGGCGGGTGTCGGGGCTGGCTTAACTATGCGGCATCAGAGCAGATTGTACTGAGAGTGCACCATATGCGGTGTGAAATACCGCACAGATGCGTAAGGAGAAAATACCGCATCAGGCGCCATTCGCCATTCAGGCTGCGCAACTGTTGGGAAGGGCGATCGGTGCGGGCCTCTTCGCTATTACGCCAGCTGGCGAAAGGGGGATGTGCTGCAAGGCGATTAAGTTGGGTAACGCCAGGGTTTTCCCAGTCACGACGTTGTAAAACGACGGCCAGTGCCAAGCTTGCATGCCTGCAGGTCGACGATTCAGCGTGTGGCAGCATATCAGTGGACGAGTCGTATCCAGTGCGAATACCTCGGACCCTGCACAATCTCTAGAGGATCCCCGGGTACCGAGCTCGAATTCGTAATCAGGTCCAATT

NOVEL23 5-TTCGGGATTTTAAAGTGCGGG-3

RT-primer：5-GTCGTATCCAGTGCAGGGTCCGAGGTATTCGCACTGGATACGACCCCGCA-3

Forward primer： 5-CAGCGTTCGGGATTTTAAAG-3

Amplicon was verified by sequencing

AGCTACCACGAGAATCCTGTGGTGTTCACCGTCGTCCGTTGTAAGCTCATCAGGTCCGTCCCAACGATCAAGGCGAGTTACATGAATCCCCCCATGTGTGCAAAAGCGGTAGCTCCTCCGTCCTCCGATCGGTGTCAGAGTAAGTGCCGCCAGTGTTATCATCATGGGTTATGGCAGCATGCATAATTCTCTTACTGTCATGCCATCCGTAAGATGCTTTCTGGGACTGGTGAGTACTCAACCAAGTCATTCTGAGAATAGTGTATGCGGCGACCGAGTTGCTCCTGCCCGGCGTCAATACGGGATAATACCGCGCCACATAGCAGAACTTTAAAAGTGCTCATCATTGGAAAACGTTCTTCGGGGCGAAAACTCTCAAGGATCTTACCGCTGTTGAGATCCAGTTCGATGTAACCCACTCGTGCACCCAACTGATCTTCAGCATCTTTTACTTTCACCAGCGTTTCTGGGTGAGCAAAAACAGGAAGGCAAAATGCCGCAAAAAAGGGAATAAGGGCGACACGGAAATGTTGAATACTCATACTCTTCCTTTTTCAATATTATTGAAGCATTTATCAGGGTTATTGTCTCATGAGCGGATACATATTTGAATGTATTTAGAAAAATAAACAAATAGGGGTTCCGCGCACATTTCCCCGAAAAGTGCCACCTGACGTCTAAGAAACCATTATTATCATGACATTAACCTATAAAAATAGGCGTATCACGAGGCCCTTTCGTCTCGCGCGTTTCGGTGATGACGGTGAAAACCTCTGACACATGCAGCTCCCGGAGACGGTCACAGCTTGTCTGTAAGCGGATGCCGGGAGCAGACAAGCCCGTCAGGGCGCGTCAGCGGGTGTTGGCGGGTGTCGGGGCTGGCTTAACTATGCGGCATCAGAGCAGATTGTACTGAGAGTGCACCATATGCGGTGTGAAATACCGCACAGATGCGTAAGGAGAAAATACCGCATCAGGCGCCATTCGCCATTCAGGCTGCGCAACTGTTGGGAAGGGCGATCGGTGCGGGCCTCTTCGCTATTACGCCAGCTGGCGAAAGGGGGATGTGCTGCAAGGCGATTAAGTTGGGTAACGCCAGGGTTTTCCCAGTCACGACGTTGTAAAACGACGGCCAGTGCCAAGCTTGCATGCCTGCAGGTCGACGATTCAGCGTTCGGGATTTTAAAGTGCGGGGTCGTATCCAGTGCGAATACCTCGGACCCTGCACAATCTCTAGAGGATCCCCGGGTACCGAGCTCGAATCGTAATCCATGTTCCAAG

NOVEL24 5-GTGGATTGGATGCGGATTTGA-3

RT-primer：5-GTCGTATCCAGTGCAGGGTCCGAGGTATTCGCACTGGATACGACTCAAAT-3

Forward primer： 5-CATAGGTGGATTGGATGCGG-3

Amplicon was verified by sequencing

GCTACCACTACGGCCACTTCGGGGTGTACACCGGCTTCGTCGTTAGTATGGCTTCATTCAGGTCCCGGTTCACGATCAAGGCGAGTAACATGATCCCCATGTTGTGGCAAAAAGCGGTTAGCTCCTCGTTCTCGATCGTTGTCAGAAGTAAGTGCGCAGTGTATCATCATGGTATGGCAGCACTGCATAATCTCTACTGTCATGCCATCCGTAGATGCTTTTCTGTGACTGGTGAGTACTCAAACCAAGTCATTCTGAGAATAGTGTATGCGGCGACCGAGTTGCTCTTGCCCGGCGTCAATACGGGATAATACCGCGCCACATAGCAGAACTTTAAAAGTGCTCATCATTGGAAAACGTTCTTCGGGGCGAAAACTCTCAAGGATCTTACCGCTGTTGAGATCCAGTTCGATGTAACCCACTCGTGCACCCAACTGATCTTCAGCATCTTTTACTTTCACCAGCGTTTCTGGGTGAGCAAAAACAGGAAGGCAAAATGCCGCAAAAAAGGGAATAAGGGCGACACGGAAATGTTGAATACTCATACTCTTCCTTTTTCAATATTATTGAAGCATTTATCAGGGTTATTGTCTCATGAGCGGATACATATTTGAATGTATTTAGAAAAATAAACAAATAGGGGTTCCGCGCACATTTCCCCGAAAAGTGCCACCTGACGTCTAAGAAACCATTATTATCATGACATTAACCTATAAAAATAGGCGTATCACGAGGCCCTTTCGTCTCGCGCGTTTCGGTGATGACGGTGAAAACCTCTGACACATGCAGCTCCCGGAGACGGTCACAGCTTGTCTGTAAGCGGATGCCGGGAGCAGACAAGCCCGTCAGGGCGCGTCAGCGGGTGTTGGCGGGTGTCGGGGCTGGCTTAACTATGCGGCATCAGAGCAGATTGTACTGAGAGTGCACCATATGCGGTGTGAAATACCGCACAGATGCGTAAGGAGAAAATACCGCATCAGGCGCCATTCGCCATTCAGGCTGCGCAACTGTTGGGAAGGGCGATCGGTGCGGGCCTCTTCGCTATTACGCCAGCTGGCGAAAGGGGGATGTGCTGCAAGGCGATTAAGTTGGGTAACGCCAGGGTTTTCCCAGTCACGACGTTGTAAAACGACGGCCAGTGCCAAGCTTGCATGCCTGCAGGTCGACGATTCATAGGTGGATTGGATGCGGATTTGAGTCGTATCCAGTGCGAATACCTCGGACCCTGCACAATCTCTAGAGGATCCCCGGGTACCGAGCTCGAATTCGTAATCATGTTCCCATTT

NOVEL25 5-GCGTACGAGGAGCCAAGCATA-3

RT-primer：5-GTCGTATCCAGTGCAGGGTCCGAGGTATTCGCACTGGATACGACTATGCT-3

Forward primer： 5-CAGCGGCGTACGAGGAGCCA-3

Amplicon was verified by sequencing

CTTATGCTACACCGAGGACATCTCGTGGTGTGTCGAACCGCTCGGTCGTGTAGCTTCATTCAGGTTCCGGTTCTATCGATCCAGGGCGGAGGTAAATGATCCCCCATGTTTGTCAAAAAGCGGTAGCTCCTTCGTTCTCGGATTCGTGTCAGAAGTAAGTGCCGCAGTGTTATCACTCATGGTTATGGCAGCACTGCATAATCTCTACTGTCATGCCATCCGTAAGATGCTTTTCTGTGACTGTGAGTACTCAACCAAGTCATTCTGAGAATAGTGTATGCGGCGACCGAGTTGCTCTTGCCCGGCGTCAATACGGGATAATACCGCGCCACATAGCAGAACTTTAAAAGTGCTCATCATTGGAAAACGTTCTTCGGGGCGAAAACTCTCAAGGATCTTACCGCTGTTGAGATCCAGTTCGATGTAACCCACTCGTGCACCCAACTGATCTTCAGCATCTTTTACTTTCACCAGCGTTTCTGGGTGAGCAAAAACAGGAAGGCAAAATGCCGCAAAAAAGGGAATAAGGGCGACACGGAAATGTTGAATACTCATACTCTTCCTTTTTCAATATTATTGAAGCATTTATCAGGGTTATTGTCTCATGAGCGGATACATATTTGAATGTATTTAGAAAAATAAACAAATAGGGGTTCCGCGCACATTTCCCCGAAAAGTGCCACCTGACGTCTAAGAAACCATTATTATCATGACATTAACCTATAAAAATAGGCGTATCACGAGGCCCTTTCGTCTCGCGCGTTTCGGTGATGACGGTGAAAACCTCTGACACATGCAGCTCCCGGAGACGGTCACAGCTTGTCTGTAAGCGGATGCCGGGAGCAGACAAGCCCGTCAGGGCGCGTCAGCGGGTGTTGGCGGGTGTCGGGGCTGGCTTAACTATGCGGCATCAGAGCAGATTGTACTGAGAGTGCACCATATGCGGTGTGAAATACCGCACAGATGCGTAAGGAGAAAATACCGCATCAGGCGCCATTCGCCATTCAGGCTGCGCAACTGTTGGGAAGGGCGATCGGTGCGGGCCTCTTCGCTATTACGCCAGCTGGCGAAAGGGGGATGTGCTGCAAGGCGATTAAGTTGGGTAACGCCAGGGTTTTCCCAGTCACGACGTTGTAAAACGACGGCCAGTGCCAAGCTTGCATGCCTGCAGGTCGACGATCAGCGGCGTACGAGGAGCCAAGCATAGTCGTATCCAGTGCGAATACCTCGGACCCTGCACAATCTCTAGAGGATCCCCGGGTACCGAGCTCGAATTCGTAATCAGGTCCAATT

NOVEL26 5-TTTGACTACAATTGCATGACA-3

RT-primer：5-GTCGTATCCAGTGCAGGGTCCGAGGTATTCGCACTGGATACGACTGTCAT-3

Forward primer： 5-CAGCCTTTGACTACAATTGC-3

Amplicon was verified by sequencing

TCCCTTTGTCTACACAGGACTGTGGTTACCCGCTCGTCGTAGGATGTCTCATCAGGTTCCGGTTCAGAATCAAGGCGAGTACATGATCCCCATGTTGTGCAAAAGCGGTAGTCCTCGTCTCCGATCGTGTCAGAAGTAGTGCCGGCAGTGTTATCATCATGGTTATGGCAGCATGCATATTCTCTACTGTCATGCCATCCGTAAGATGCTTTTCTGTGACTGTGAGTACTCAACCAAGTCATTCTGAGAATAGTGTATGCGGCGACCGAGTTGCTCTTGCCCGGCGTCAATACGGGATAATACCGCGCCACATAGCAGAACTTTAAAAGTGCTCATCATTGGAAAACGTTCTTCGGGGCGAAAACTCTCAAGGATCTTACCGCTGTTGAGATCCAGTTCGATGTAACCCACTCGTGCACCCAACTGATCTTCAGCATCTTTTACTTTCACCAGCGTTTCTGGGTGAGCAAAAACAGGAAGGCAAAATGCCGCAAAAAAGGGAATAAGGGCGACACGGAAATGTTGAATACTCATACTCTTCCTTTTTCAATATTATTGAAGCATTTATCAGGGTTATTGTCTCATGAGCGGATACATATTTGAATGTATTTAGAAAAATAAACAAATAGGGGTTCCGCGCACATTTCCCCGAAAAGTGCCACCTGACGTCTAAGAAACCATTATTATCATGACATTAACCTATAAAAATAGGCGTATCACGAGGCCCTTTCGTCTCGCGCGTTTCGGTGATGACGGTGAAAACCTCTGACACATGCAGCTCCCGGAGACGGTCACAGCTTGTCTGTAAGCGGATGCCGGGAGCAGACAAGCCCGTCAGGGCGCGTCAGCGGGTGTTGGCGGGTGTCGGGGCTGGCTTAACTATGCGGCATCAGAGCAGATTGTACTGAGAGTGCACCATATGCGGTGTGAAATACCGCACAGATGCGTAAGGAGAAAATACCGCATCAGGCGCCATTCGCCATTCAGGCTGCGCAACTGTTGGGAAGGGCGATCGGTGCGGGCCTCTTCGCTATTACGCCAGCTGGCGAAAGGGGGATGTGCTGCAAGGCGATTAAGTTGGGTAACGCCAGGGTTTTCCCAGTCACGACGTTGTAAAACGACGGCCAGTGCCAAGCTTGCATGCCTGCAGGTCGACGATTCAGCCTTTGACTACAATTGCATGACAGTCGTATCCAGTGCGAATACCTCGGACCCTGCACAATCTCTAGAGGATCCCCGGGTACCGAGCTCGAATCGTAATCATTTTCATTTTT

NOVEL27 5-TCGGACCAGGCTTCATTCCCCT-3

RT-primer：5-GTCGTATCCAGTGCAGGGTCCGAGGTATTCGCACTGGATACGACAGGGGA-3

Forward primer： 5-CGGCGTCGGACCAGGCTTCAT-3

Amplicon was verified by sequencing

ATGGAAAATGATTACGAATTCGAGCTCGGTACCCGGGGATCCTCTAGAGATTCGGCGTCGGACCAGGCTTCATCCCCTGTCGTATCCAGTGCGAATACCTCGGACCCTGCACAATCGTCGACCTGCAGGCATGCAAGCTTGGCACTGGCCGTCGTTTTACAACGTCGTGACTGGGAAAACCCTGGCGTTACCCAACTTAATCGCCTTGCAGCACATCCCCCTTTCGCCAGCTGGCGTAATAGCGAAGAGGCCCGCACCGATCGCCCTTCCCAACAGTTGCGCAGCCTGAATGGCGAATGGCGCCTGATGCGGTATTTTCTCCTTACGCATCTGTGCGGTATTTCACACCGCATATGGTGCACTCTCAGTACAATCTGCTCTGATGCCGCATAGTTAAGCCAGCCCCGACACCCGCCAACACCCGCTGACGCGCCCTGACGGGCTTGTCTGCTCCCGGCATCCGCTTACAGACAAGCTGTGACCGTCTCCGGGAGCTGCATGTGTCAGAAGTTTTCACCGTCATCACCGAAACGCGCGAGACGAAAGGGCCTCGTGATACGCCTATTTTTATAGGTTAATGTCATGATAATAATGGTTTCTTAGACGTCAGGTGGCACTTTTCGGGGAAATGTGCGCGGAACCCCTATTTGTTTATTTTTCTAAATACATTCAAATATGTATCCGCTCATGAGACAATAACCCTGATAAATGCTTCAATAATATTGAAAAAGGAAGAGTATGAGTATTCAACATTTCCGTGTCGCCCTTATTCCCTTTTTTGCGGCATTTTGCCTTCCTGTTTTTGCTCACCCAGAAACGCTGGTGAAAGTAAAAGATGCTGAAGATCAGTTGGGTGCACGAGTGGGTTACATCGAACTGGATCTCAACAGCGGTAAGATCCTTGAGAGTTTTCGCCCCGAAGAACGTTTTCCAATGATGAGCACTTTTAAAGTTCTGCTATGTGGCGCGGTATTATCCCGTATTGACGCCGGCAGAGCAACTCGGTCGCCGCATACACTATTCTCAGATGACTTGGTTGAGTACTCACCAGTCACAGAAGCATCTTACGATGGCATGACAGTAGAGATTATGCATGCTGCATACATGATGATAACACTGCGGCCACTACTTCTGACACGATCGAGACGGAGGAACTACGCTTTGCACAACATGGATCATGTAACTCGCTTGTATCTATGGACGGACTGATGACCATCCAACGCAGAGCGTCAACATCACGATCCTGGTAGCAATG

NOVEL28 5-TTGGACAGAGAAATCACGGTCA-3

RT-primer：5-GTCGTATCCAGTGCAGGGTCCGAGGTATTCGCACTGGATACGACTGACCG-3

Forward primer： 5-CGGCGTTGGACAGAGAAATCA-3

Amplicon was verified by sequencing

GGTATATGGCGTTCATTTCAGGCTCCCGGTCCCAACGATACAGGCGAGTAACATGATTCCCCCATGTTGTGCAAAAAGCGTAGTTCGTCGTCCTCCGATCGTGTCAGAAGGTAGGTGGCCGCCAGTGTATCATCATGTATGCAGCACTGCATAATCTCTACTGTCATGCCATCGTAAGATGCTTTCTGTGACTGGTGAGTACTCAACCAAGTCATCCTGAGAATAGTGTATGCGGCGACCGAGTTGCTCTGCCCGGGCGTCAATACGGGATAATACCGCGCCACATAGCAGAACTTTAAAAGTGCTCATCATTGGAAAACGTTCTTCGGGGCGAAAACTCTCAAGGATCTTACCGCTGTTGAGATCCAGTTCGATGTAACCCACTCGTGCACCCAACTGATCTTCAGCATCTTTTACTTTCACCAGCGTTTCTGGGTGAGCAAAAACAGGAAGGCAAAATGCCGCAAAAAAGGGAATAAGGGCGACACGGAAATGTTGAATACTCATACTCTTCCTTTTTCAATATTATTGAAGCATTTATCAGGGTTATTGTCTCATGAGCGGATACATATTTGAATGTATTTAGAAAAATAAACAAATAGGGGTTCCGCGCACATTTCCCCGAAAAGTGCCACCTGACGTCTAAGAAACCATTATTATCATGACATTAACCTATAAAAATAGGCGTATCACGAGGCCCTTTCGTCTCGCGCGTTTCGGTGATGACGGTGAAAACCTCTGACACATGCAGCTCCCGGAGACGGTCACAGCTTGTCTGTAAGCGGATGCCGGGAGCAGACAAGCCCGTCAGGGCGCGTCAGCGGGTGTTGGCGGGTGTCGGGGCTGGCTTAACTATGCGGCATCAGAGCAGATTGTACTGAGAGTGCACCATATGCGGTGTGAAATACCGCACAGATGCGTAAGGAGAAAATACCGCATCAGGCGCCATTCGCCATTCAGGCTGCGCAACTGTTGGGAAGGGCGATCGGTGCGGGCCTCTTCGCTATTACGCCAGCTGGCGAAAGGGGGATGTGCTGCAAGGCGATTAAGTTGGGTAACGCCAGGGTTTTCCCAGTCACGACGTTGTAAAACGACGGCCAGTGCCAAGCTTGCATGCCTGCAGGTCGACGATTCGCCGTTGGACAGAGAAATCACGGTCAGTCGTATCCAGTGCGAATACCTCGGACCCTGCACAATCTCTAGAGGATCCCCGGGTACCGAGCTCGAATCGTAATCATCCAATGC

NOVEL29 5-TCGCTTGGTGCAGGTCGGGAA-3

RT-primer：5-GTCGTATCCAGTGCAGGGTCCGAGGTATTCGCACTGGATACGACTTCCCG-3

Forward primer： 5-CATAGTCGCTTGGTGCAGGT-3

Amplicon was verified by sequencing

ATGGAAAATGATTACGAATTCGAGCTCGGTACCCGGGGATCCTCTAGAGATTCATAGTCGCTTGGTGCAGGTCGGGAAGTCGTATCCAGTGCGAATACCTCGGACCCTGCACAATCGTCGACCTGCAGGCATGCAAGCTTGGCACTGGCCGTCGTTTTACAACGTCGTGACTGGGAAAACCCTGGCGTTACCCAACTTAATCGCCTTGCAGCACATCCCCCTTTCGCCAGCTGGCGTAATAGCGAAGAGGCCCGCACCGATCGCCCTTCCCAACAGTTGCGCAGCCTGAATGGCGAATGGCGCCTGATGCGGTATTTTCTCCTTACGCATCTGTGCGGTATTTCACACCGCATATGGTGCACTCTCAGTACAATCTGCTCTGATGCCGCATAGTTAAGCCAGCCCCGACACCCGCCAACACCCGCTGACGCGCCCTGACGGGCTTGTCTGCTCCCGGCATCCGCTTACAGACAAGCTGTGACCGTCTCCGGGAGCTGCATGTGTCAGAAGTTTTCACCGTCATCACCGAAACGCGCGAGACGAAAGGGCCTCGTGATACGCCTATTTTTATAGGTTAATGTCATGATAATAATGGTTTCTTAGACGTCAGGTGGCACTTTTCGGGGAAATGTGCGCGGAACCCCTATTTGTTTATTTTTCTAAATACATTCAAATATGTATCCGCTCATGAGACAATAACCCTGATAAATGCTTCAATAATATTGAAAAAGGAAGAGTATGAGTATTCAACATTTCCGTGTCGCCCTTATTCCCTTTTTTGCGGCATTTTGCCTTCCTGTTTTTGCTCACCCAGAAACGCTGGTGAAAGTAAAAGATGCTGAAGATCAGTTGGGTGCACGAGTGGGTTACATCGAACTGGATCTCAACAGCGGTAAGATCCTTGAGAGTTTTCGCCCCGAAGAACGTTTTCCAATGATGAGCACTTTTAAAGTTCTGCTATGTGGCGCGGTATTATCCCGTATTGACGCCGGCAGAGCAACTCGGTCGCCGCATACACTATTCTCAGATGACTTGGTTGAGTACTCACCAGTCACAGAAGCATCTTACGATGGCATGACAGTAGAGATTATGCATGCTGCATACATGATGATAACACTGCGGCCACTACTTCTGACACGATCGAGACGGAGGAACTACGCTTTGCACAACATGGATCATGTAACTCGCTTGTATCTATGGACGGACTGATGACCATCCAACGCAGAGCGTCAACATCACGATCCTGGTAGCAATG

NOVEL30 5-AAGCCAAAATGACACCCTTTCCT-3

RT-primer：5-GTCGTATCCAGTGCAGGGTCCGAGGTATTCGCACTGGATACGACAGGAAA-3

Forward primer： 5-CAGCGAAGCCAAAATGACACCC-3

Amplicon was verified by sequencing

AACCTGGAACATGATTACGATTCGAGCTCGGTACCCGGGGATCCTCTAGAGATTCAGCGAAGCCAAAATGACACCCTTCCTGTCGTATCCAGTGCGAATACCTCGGACCCTGCACAATCGTCGACCTGCAGGCATGCAAGCTTGGCACTGGCCGTCGTTTTACAACGTCGTGACTGGGAAAACCCTGGCGTTACCCAACTTAATCGCCTTGCAGCACATCCCCCTTTCGCCAGCTGGCGTAATAGCGAAGAGGCCCGCACCGATCGCCCTTCCCAACAGTTGCGCAGCCTGAATGGCGAATGGCGCCTGATGCGGTATTTTCTCCTTACGCATCTGTGCGGTATTTCACACCGCATATGGTGCACTCTCAGTACAATCTGCTCTGATGCCGCATAGTTAAGCCAGCCCCGACACCCGCCAACACCCGCTGACGCGCCCTGACGGGCTTGTCTGCTCCCGGCATCCGCTTACAGACAAGCTGTGACCGTCTCCGGGAGCTGCATGTGTCAGAGGTTTTCACCGTCATCACCGAAACGCGCGAGACGAAAGGGCCTCGTGATACGCCTATTTTTATAGGTTAATGTCATGATAATAATGGTTTCTTAGACGTCAGGTGGCACTTTTCGGGGAAATGTGCGCGGAACCCCTATTTGTTTATTTTTCTAAATACATTCAAATATGTATCCGCTCATGAGACAATAACCCTGATAAATGCTTCAATAATATTGAAAAAGGAAGAGTATGAGTATTCAACATTTCCGTGTCGCCCTTATTCCCTTTTTTGCGGCATTTTGCCTTCCTGTTTTTGCTCACCCAGAAACGCTGGTGAAAGTAAAAGATGCTGAAGATCAGTTGGGTGCACGAGTGGGTTACATCGAACTGGATCTCAACAGCGGTAAGATCCTTGAGAGTTTTCGCCCCGAAGAACGTTTTCCAATGATGAGCACTTTTAAAGTTCTGCTATGTGGCGCGGTATTATCCCGTATTGACGCCCGGGCAAGAGCAACTCGGTCGCCCGCATACACTATTCTCAGAATGACTTGGTTTGAGTACCTCCACCAGTCACAAGAAAAAGCATTCTTACGGATGGGCATGACAGGTAAGGAGAATTATGCAGTGCTGCCATAACCATGGAGTGGATAACACTGCCGACAACTTACTTTCTGACAACGAATCCGAAGAACGAGGAGCTATCGCTTTTGGCCAACATGGGGGTATCATGTTACTCCGCCCCTTGTAATCGTGGGGAACCCCGGGAACCT

NOVEL31 5-AAGGATGTATCTTTTGTCTTTGA-3

RT-primer：5-GTCGTATCCAGTGCAGGGTCCGAGGTATTCGCACTGGATACGACTCAAAG-3

Forward primer： 5-CAGCGAAGGATGTATCTTTTGT-3

Amplicon was verified by sequencing

TAATATGTTTTAAAAATTCTAGCTCGGTACCCGGGGACCTCTAGAGATTCAGCGAAGGATGTATCTTTTGTCTTTGAGTCGTATTCAGTGCGAATACCTCGGACCCTGCACAATCGTCGACCTGCAGGCATGCAAGCTTGGCACTGGCCGTCGTTTTACAACGTCGTGACTGGGAAAACCCTGGCGTTACCCAACTTAATCGCCTTGCAGCACATCCCCCTTTCGCCAGCTGGCGTAATAGCGAAGAGGCCCGCACCGATCGCCCTTCCCAACAGTTGCGCAGCCTGAATGGCGAATGGCGCCTGATGCGGTATTTTCTCCTTACGCATCTGTGCGGTATTTCACACCGCATATGGTGCACTCTCAGTACAATCTGCTCTGATGCCGCATAGTTAAGCCAGCCCCGACACCCGCCAACACCCGCTGACGCGCCCTGACGGGCTTGTCTGCTCCCGGCATCCGCTTACAGACAAGCTGTGACCGTCTCCGGGAGCTGCATGTGTCAGAGGTTTTCACCGTCATCACCGAAACGCGCGAGACGAAAGGGCCTCGTGATACGCCTATTTTTATAGGTTAATGTCATGATAATAATGGTTTCTTAGACGTCAGGTGGCACTTTTCGGGGAAATGTGCGCGGAACCCCTATTTGTTTATTTTTCTAAATACATTCAAATATGTATCCGCTCATGAGACAATAACCCTGATAAATGCTTCAATAATATTGAAAAAGGAAGAGTATGAGTATTCAACATTTCCGTGTCGCCCTTATTCCCTTTTTTGCGGCATTTTGCCTTCCTGTTTTTGCTCACCCAGAAACGCTGGTGAAAGTAAAAGATGCTGAAAGATCAGTTGGGTGCACGAGTGGGTTACATCGAACTGGATCTCAACAGCGGTAAGATCCTTGAGAGTTTTCGCCCCGAAAGAACGTTTTCCAATGATGAGCACTTTTAAAGTTCTGCTATGTGGCGCGTATTATCCCGTATTGACGCCGGGCAAGAGCAACTCGGTCGCGCATTACACTAATTCTCAGAATGACTTGCTTGAGTACTCAACAGTCAAAGAAAAAGCATCTTACGATTGCATGACAGTAGGAAGAATATGCAATGCTGCATAAACCATGAATGAAAAACACTTGCCGCCAACCTAACTTCTTGACAACCGATCGAGAACGAGGACCTAACGCCTTTTTGCAAACATGAGGATCAGTTAACCTCGCATGATCTCGTGGGGTAAACCCGCGAACGCT

NOVEL32 5-CGCACCCCAGCGTGGAACCATC-3

RT-primer：5-GTCGTATCCAGTGCAGGGTCCGAGGTATTCGCACTGGATACGACGATGGT-3

Forward primer： 5-CGCCGCGCACCCCAGCGTGGA-3

Amplicon was verified by sequencing

GGTATATGGCGTTCATTTCAGGCTCCCGGTCCCAACGATACAGGCGAGTAACATGATTCCCCCATGTTGTGCAAAAAGCGTAGTTCGTCGTCCTCCGATCGTGTCAGAAGGTAGGTGGCCGCCAGTGTATCATCATGTATGCAGCACTGCATAATCTCTACTGTCATGCCATCGTAAGATGCTTTCTGTGACTGGTGAGTACTCAACCAAGTCATCCTGAGAATAGTGTATGCGGCGACCGAGTTGCTCTGCCCGGGCGTCAATACGGGATAATACCGCGCCACATAGCAGAACTTTAAAAGTGCTCATCATTGGAAAACGTTCTTCGGGGCGAAAACTCTCAAGGATCTTACCGCTGTTGAGATCCAGTTCGATGTAACCCACTCGTGCACCCAACTGATCTTCAGCATCTTTTACTTTCACCAGCGTTTCTGGGTGAGCAAAAACAGGAAGGCAAAATGCCGCAAAAAAGGGAATAAGGGCGACACGGAAATGTTGAATACTCATACTCTTCCTTTTTCAATATTATTGAAGCATTTATCAGGGTTATTGTCTCATGAGCGGATACATATTTGAATGTATTTAGAAAAATAAACAAATAGGGGTTCCGCGCACATTTCCCCGAAAAGTGCCACCTGACGTCTAAGAAACCATTATTATCATGACATTAACCTATAAAAATAGGCGTATCACGAGGCCCTTTCGTCTCGCGCGTTTCGGTGATGACGGTGAAAACCTCTGACACATGCAGCTCCCGGAGACGGTCACAGCTTGTCTGTAAGCGGATGCCGGGAGCAGACAAGCCCGTCAGGGCGCGTCAGCGGGTGTTGGCGGGTGTCGGGGCTGGCTTAACTATGCGGCATCAGAGCAGATTGTACTGAGAGTGCACCATATGCGGTGTGAAATACCGCACAGATGCGTAAGGAGAAAATACCGCATCAGGCGCCATTCGCCATTCAGGCTGCGCAACTGTTGGGAAGGGCGATCGGTGCGGGCCTCTTCGCTATTACGCCAGCTGGCGAAAGGGGGATGTGCTGCAAGGCGATTAAGTTGGGTAACGCCAGGGTTTTCCCAGTCACGACGTTGTAAAACGACGGCCAGTGCCAAGCTTGCATGCCTGCAGGTCGACGATTCGCCGCGCACCCCAGCGTGGAACCATCGTCGTATCCAGTGCGAATACCTCGGACCCTGCACAATCTCTAGAGGATCCCCGGGTACCGAGCTCGAATCGTAATCATCCAATGC

NOVEL33 5-CAGCCAAGGATGACTTGCCGG-3

RT-primer：5-GTCGTATCCAGTGCAGGGTCCGAGGTATTCGCACTGGATACGACCCGGCA-3

Forward primer： 5-CATCGCAGCCAAGGATGACT-3

Amplicon was verified by sequencing

TGACATTGAACATGATTACGATTCGAGCTCGGTACCCGGGGATCCTCTAGAGATTCATCGCAGCCAAGGATGACTTGCCGGGTCGTATCCAGTGCGAATACCTCGGACCCTGCACAATCGTCGACCTGCAGGCATGCAAGCTTGGCACTGGCCGTCGTTTTACAACGTCGTGACTGGGAAAACCCTGGCGTTACCCAACTTAATCGCCTTGCAGCACATCCCCCTTTCGCCAGCTGGCGTAATAGCGAAGAGGCCCGCACCGATCGCCCTTCCCAACAGTTGCGCAGCCTGAATGGCGAATGGCGCCTGATGCGGTATTTTCTCCTTACGCATCTGTGCGGTATTTCACACCGCATATGGTGCACTCTCAGTACAATCTGCTCTGATGCCGCATAGTTAAGCCAGCCCCGACACCCGCCAACACCCGCTGACGCGCCCTGACGGGCTTGTCTGCTCCCGGCATCCGCTTACAGACAAGCTGTGACCGTCTCCGGGAGCTGCATGTGTCAGAGGTTTTCACCGTCATCACCGAAACGCGCGAGACGAAAGGGCCTCGTGATACGCCTATTTTTATAGGTTAATGTCATGATAATAATGGTTTCTTAGACGTCAGGTGGCACTTTTCGGGGAAATGTGCGCGGAACCCCTATTTGTTTATTTTTCTAAATACATTCAAATATGTATCCGCTCATGAGACAATAACCCTGATAAATGCTTCAATAATATTGAAAAAGGAAGAGTATGAGTATTCAACATTTCCGTGTCGCCCTTATTCCCTTTTTTGCGGCATTTTGCCTTCCTGTTTTTGCTCACCCAGAAACGCTGGTGAAAGTAAAAGATGCTGAAGATCAGTTGGGTGCACGAGTGGGTTACATCGAACTGGATCTCAACAGCGGTAAGATCCTTGAGAGTTTTCGCCCCGAAGAACGTTTTCCAATGATGAGCACTTTTAAAGTTCTGCTATGTGGCGCGGTATTATCCCGTATTGACGCCGGGCAAGAGCAACTCGGTCGCCGCATACACTATTCTCAGATGACTTGGTTGAGTACTCACAGTCACAGAAAAGCATCTTACGGATGGCATGACAGTAGAGATTATGCATGCTGCATAACCATGGAGGGATAACACTGCCGCACTACTTCTGACACGATCGGAGGACGGAGGACTAACCGCTTTTTGCACAACATGGGGATCATGTACTCGCCTTGTATGTGGAACCGGACCTGATGACAAACATCGACGAGCGTAACCCAATGTCCGTTACACATATGGGA

NOVEL34 5-TGAAGCTGCCAGCATGATCTA-3

RT-primer：5-GTCGTATCCAGTGCAGGGTCCGAGGTATTCGCACTGGATACGACTAGATC-3

Forward primer： 5-CATCGTGAAGCTGCCAGCAT-3

Amplicon was verified by sequencing

CATTGAACCTGCATTACGATTCGAGCTCGGTACCCGGGGATCCTCTAGAGATTCATCGTGAAGCTGCCAGCATGATCTAGTCGTATCCAGTGCGAATACCTCGGACCCTGCACAATCGTCGACCTGCAGGCATGCAAGCTTGGCACTGGCCGTCGTTTTACAACGTCGTGACTGGGAAAACCCTGGCGTTACCCAACTTAATCGCCTTGCAGCACATCCCCCTTTCGCCAGCTGGCGTAATAGCGAAGAGGCCCGCACCGATCGCCCTTCCCAACAGTTGCGCAGCCTGAATGGCGAATGGCGCCTGATGCGGTATTTTCTCCTTACGCATCTGTGCGGTATTTCACACCGCATATGGTGCACTCTCAGTACAATCTGCTCTGATGCCGCATAGTTAAGCCAGCCCCGACACCCGCCAACACCCGCTGACGCGCCCTGACGGGCTTGTCTGCTCCCGGCATCCGCTTACAGACAAGCTGTGACCGTCTCCGGGAGCTGCATGTGTCAGAGGTTTTCACCGTCATCACCGAAACGCGCGAGACGAAAGGGCCTCGTGATACGCCTATTTTTATAGGTTAATGTCATGATAATAATGGTTTCTTAGACGTCAGGTGGCACTTTTCGGGGAAATGTGCGCGGAACCCCTATTTGTTTATTTTTCTAAATACATTCAAATATGTATCCGCTCATGAGACAATAACCCTGATAAATGCTTCAATAATATTGAAAAAGGAAGAGTATGAGTATTCAACATTTCCGTGTCGCCCTTATTCCCTTTTTTGCGGCATTTTGCCTTCCTGTTTTTGCTCACCCAGAAACGCTGGTGAAAGTAAAAGATGCTGAAGATCAGTTGGGTGCACGAGTGGGTTACATCGAACTGGATCTCAACAGCGGTAAGATCCTTGAGAGTTTTCGCCCCGAAGAACGTTTTCCAATGATGAGCACTTTTAAAGTTCTGCTATGTGGCGCGGTATTATCCCGTATTGACGCCGGGCAAGAGCAACTCGGTCGCCGCATACACTATCTCAGAATGACTTGGTTGAGTACTCACAGTCACAGAAAAGCATCTTACGGATGGCATGACAGTAGAGATTATGCAGTGCTGCCATAACCATGGAGTGATAACACTGCGCAACTAACTTCTGACACGATCGGAGACGACGAGCTACGGCTTTTTGCACACCATGGGGATCATGTACTCGCCCTTGTATCGTTGAACGGACCTGAATGAAGCCATTACCTAACGACGCGAGCGGTGATCACCACGAAATGTCCCGTGGTATACCAATAGTAGGGGC

NOVEL35 5-GTGCTCTCTACCATTGTCATA-3

RT-primer：5-GTCGTATCCAGTGCAGGGTCCGAGGTATTCGCACTGGATACGACTATGAC-3

Forward primer： 5-CATCGGTGCTCTCTACCATT-3

Amplicon was verified by sequencing

AAAAATGAAATGATTACGATTCGAGCTCGGTACCCGGGGATCCTCTAGAGATTCATCGGTGCTCTCTACCATTGTCATAGTCGTATCCAGTGCGAATACCTCGGACCCTGCACAATCGTCGACCTGCAGGCATGCAAGCTTGGCACTGGCCGTCGTTTTACAACGTCGTGACTGGGAAAACCCTGGCGTTACCCAACTTAATCGCCTTGCAGCACATCCCCCTTTCGCCAGCTGGCGTAATAGCGAAGAGGCCCGCACCGATCGCCCTTCCCAACAGTTGCGCAGCCTGAATGGCGAATGGCGCCTGATGCGGTATTTTCTCCTTACGCATCTGTGCGGTATTTCACACCGCATATGGTGCACTCTCAGTACAATCTGCTCTGATGCCGCATAGTTAAGCCAGCCCCGACACCCGCCAACACCCGCTGACGCGCCCTGACGGGCTTGTCTGCTCCCGGCATCCGCTTACAGACAAGCTGTGACCGTCTCCGGGAGCTGCATGTGTCAGAGGTTTTCACCGTCATCACCGAAACGCGCGAGACGAAAGGGCCTCGTGATACGCCTATTTTTATAGGTTAATGTCATGATAATAATGGTTTCTTAGACGTCAGGTGGCACTTTTCGGGGAAATGTGCGCGGAACCCCTATTTGTTTATTTTTCTAAATACATTCAAATATGTATCCGCTCATGAGACAATAACCCTGATAAATGCTTCAATAATATTGAAAAAGGAAGAGTATGAGTATTCAACATTTCCGTGTCGCCCTTATTCCCTTTTTTGCGGCATTTTGCCTTCCTGTTTTTGCTCACCCAGAAACGCTGGTGAAAGTAAAAGATGCTGAAGATCAGTTGGGTGCACGAGTGGGTTACATCGAACTGGATCTCAACAGCGGTAAGATCCTTGAGAGTTTTCGCCCCGAAGAACGTTTTCCAATGATGAGCACTTTTAAAGTTCTGCTATGTGGCGCGGTATTATCCCGTATTGACGCCGGGCAAGAGCAACTCGGTCGCCGCATACACTATTCTCAGAATGACTTGGTTGAGTACTCACCAGTCACAGAAAAGCATCTTACGGATGGCATGACAGTAGAGATTATGCATGCTGCCATAACCATGGAGTGAAAACACTGCGCACTACTTCTGACAACGATCGGAGGACCGAGGACTACGCTTTTGCCCAACATGGGGGAACATGTAACTCGCCTTGATTCGTTGGGACCGACCTGATGGCCACCAACGGACGAGGCGTTAATCACAGAAGTTCCTGGTTACAAATGTGGCGAACACACAACATGTC

NOVEL36 5-TTGTGCTGAGCACCGGATCAA-3

RT-primer：5-GTCGTATCCAGTGCAGGGTCCGAGGTATTCGCACTGGATACGACTTGATC-3

Forward primer： 5-CAGCGTTGTGCTGAGCACCG-3

Amplicon was verified by sequencing

ATGGAACCATGATTACGATTCGAGCTCGGTACCCGGGGATCCTCTAGAGATTCAGCGTTGTGCTGAGCACCGGATCAAGTCGTATCCAGTGCGAATACCTCGGACCCTGCACAATCGTCGACCTGCAGGCATGCAAGCTTGGCACTGGCCGTCGTTTTACAACGTCGTGACTGGGAAAACCCTGGCGTTACCCAACTTAATCGCCTTGCAGCACATCCCCCTTTCGCCAGCTGGCGTAATAGCGAAGAGGCCCGCACCGATCGCCCTTCCCAACAGTTGCGCAGCCTGAATGGCGAATGGCGCCTGATGCGGTATTTTCTCCTTACGCATCTGTGCGGTATTTCACACCGCATATGGTGCACTCTCAGTACAATCTGCTCTGATGCCGCATAGTTAAGCCAGCCCCGACACCCGCCAACACCCGCTGACGCGCCCTGACGGGCTTGTCTGCTCCCGGCATCCGCTTACAGACAAGCTGTGACCGTCTCCGGGAGCTGCATGTGTCAGAGGTTTTCACCGTCATCACCGAAACGCGCGAGACGAAAGGGCCTCGTGATACGCCTATTTTTATAGGTTAATGTCATGATAATAATGGTTTCTTAGACGTCAGGTGGCACTTTTCGGGGAAATGTGCGCGGAACCCCTATTTGTTTATTTTTCTAAATACATTCAAATATGTATCCGCTCATGAGACAATAACCCTGATAAATGCTTCAATAATATTGAAAAAGGAAGAGTATGAGTATTCAACATTTCCGTGTCGCCCTTATTCCCTTTTTTGCGGCATTTTGCCTTCCTGTTTTTGCTCACCCAGAAACGCTGGTGAAAGTAAAAGATGCTGAAGATCAGTTGGGTGCACGAGTGGGTTACATCGAACTGGATCTCAACAGCGGTAAGATCCTTGAGAGTTTTCGCCCCGAAGAACGTTTTCCAATGATGAGCACTTTTAAAGTTCTGCTATGTGGCGCGGTATTATCCCGTATTGACGCCGGGCAAGAGCAACTCGGTCGCCGCATACACTATTCTCAGAATGACTTGGTTGAGTACTCACAGTCACAGAAAAGCATCTTACGGATGGCATGACAGTAGAGATTATGCAGTGCTGCCATAACCATGAGTGATACACTGCGCACTACTTCTGACACGATCGGAAGACCGAGAGCTACCGCTTTTTTGCACAAACATGGGGGATCATGTACTCGCCTTGATCGTTGGACCGAACCTGAATGAGCCAAGACGAACGAGCGTGACACACCGAAGGCCGTGTACAAAATGG

NOVEL37 5-AACAAGCGTGTAGATCAGCTG-3

RT-primer：5-GTCGTATCCAGTGCAGGGTCCGAGGTATTCGCACTGGATACGACCAGCTG-3

Forward primer： 5-CGGCGAACAAGCGTGTAGAT-3

Amplicon was verified by sequencing

CATTGAACCTGCATTACGATTCGAGCTCGGTACCCGGGGATCCTCTAGAGATTCGGCGAACAAGCGTGTAGATCAGCTGGTCGTATCCAGTGCGAATACCTCGGACCCTGCACAATCGTCGACCTGCAGGCATGCAAGCTTGGCACTGGCCGTCGTTTTACAACGTCGTGACTGGGAAAACCCTGGCGTTACCCAACTTAATCGCCTTGCAGCACATCCCCCTTTCGCCAGCTGGCGTAATAGCGAAGAGGCCCGCACCGATCGCCCTTCCCAACAGTTGCGCAGCCTGAATGGCGAATGGCGCCTGATGCGGTATTTTCTCCTTACGCATCTGTGCGGTATTTCACACCGCATATGGTGCACTCTCAGTACAATCTGCTCTGATGCCGCATAGTTAAGCCAGCCCCGACACCCGCCAACACCCGCTGACGCGCCCTGACGGGCTTGTCTGCTCCCGGCATCCGCTTACAGACAAGCTGTGACCGTCTCCGGGAGCTGCATGTGTCAGAGGTTTTCACCGTCATCACCGAAACGCGCGAGACGAAAGGGCCTCGTGATACGCCTATTTTTATAGGTTAATGTCATGATAATAATGGTTTCTTAGACGTCAGGTGGCACTTTTCGGGGAAATGTGCGCGGAACCCCTATTTGTTTATTTTTCTAAATACATTCAAATATGTATCCGCTCATGAGACAATAACCCTGATAAATGCTTCAATAATATTGAAAAAGGAAGAGTATGAGTATTCAACATTTCCGTGTCGCCCTTATTCCCTTTTTTGCGGCATTTTGCCTTCCTGTTTTTGCTCACCCAGAAACGCTGGTGAAAGTAAAAGATGCTGAAGATCAGTTGGGTGCACGAGTGGGTTACATCGAACTGGATCTCAACAGCGGTAAGATCCTTGAGAGTTTTCGCCCCGAAGAACGTTTTCCAATGATGAGCACTTTTAAAGTTCTGCTATGTGGCGCGGTATTATCCCGTATTGACGCCGGGCAAGAGCAACTCGGTCGCCGCATACACTATCTCAGAATGACTTGGTTGAGTACTCACAGTCACAGAAAAGCATCTTACGGATGGCATGACAGTAGAGATTATGCAGTGCTGCCATAACCATGGAGTGATAACACTGCGCAACTAACTTCTGACACGATCGGAGACGACGAGCTACGGCTTTTTGCACACCATGGGGATCATGTACTCGCCCTTGTATCGTTGAACGGACCTGAATGAAGCCATTACCTAACGACGCGAGCGGTGATCACCACGAAATGTCCCGTGGTATACCAATAGTAGGGGC

NOVEL38 5-TGCCAGCATCCTGTGACAAGA-3

RT-primer：5-GTCGTATCCAGTGCAGGGTCCGAGGTATTCGCACTGGATACGACTCTTGT-3

Forward primer： 5-CATCGTGCCAGCATCCTGTG-3

Amplicon was verified by sequencing

GTTGGAACATTGATTACGATTCGAGCTCGGTACCCGGGGATCCTCTAGAGATTCATCGTGCCAGCATCCTGTGACAAGAGTCGTATCCAGTGCGAATACCTCGGACCCTGCACAATCGTCGACCTGCAGGCATGCAAGCTTGGCACTGGCCGTCGTTTTACAACGTCGTGACTGGGAAAACCCTGGCGTTACCCAACTTAATCGCCTTGCAGCACATCCCCCTTTCGCCAGCTGGCGTAATAGCGAAGAGGCCCGCACCGATCGCCCTTCCCAACAGTTGCGCAGCCTGAATGGCGAATGGCGCCTGATGCGGTATTTTCTCCTTACGCATCTGTGCGGTATTTCACACCGCATATGGTGCACTCTCAGTACAATCTGCTCTGATGCCGCATAGTTAAGCCAGCCCCGACACCCGCCAACACCCGCTGACGCGCCCTGACGGGCTTGTCTGCTCCCGGCATCCGCTTACAGACAAGCTGTGACCGTCTCCGGGAGCTGCATGTGTCAGAGGTTTTCACCGTCATCACCGAAACGCGCGAGACGAAAGGGCCTCGTGATACGCCTATTTTTATAGGTTAATGTCATGATAATAATGGTTTCTTAGACGTCAGGTGGCACTTTTCGGGGAAATGTGCGCGGAACCCCTATTTGTTTATTTTTCTAAATACATTCAAATATGTATCCGCTCATGAGACAATAACCCTGATAAATGCTTCAATAATATTGAAAAAGGAAGAGTATGAGTATTCAACATTTCCGTGTCGCCCTTATTCCCTTTTTTGCGGCATTTTGCCTTCCTGTTTTTGCTCACCCAGAAACGCTGGTGAAAGTAAAAGATGCTGAAGATCAGTTGGGTGCACGAGTGGGTTACATCGAACTGGATCTCACAGCGGTAAGATCCTTGAGAGTTTTCGCCCCGAAGAACGTTTTCCCATGATGAGCACTTTTAAAGTTCTGCTATGTGCGCGGTATTATCCGTATTGACGCCGGGCAAGAGCACTCGGTCGCGCATACACTATTCTCAGATGACTTGTTGAGTACTCACAGTCACAGAAAAGCATCTACGATGCATGACAGTAGAGATTATGCATGCTGCATAACCATGATGATACACTGCGCACTACTCTGACACGATCGGAGGACGAGACTACCGCTTTTGCACAACATGGGATCATGTACTCGCCTTGATCGTGGACGGACTGATGACTACAAGCAGCGACGTGACCCAGCATTCCTGGTTACATGGCACTACGAC

NOVEL39 5-TGCAACTGTGGTACCGTGCTA-3

RT-primer：5-GTCGTATCCAGTGCAGGGTCCGAGGTATTCGCACTGGATACGACTAGCAC-3

Forward primer： 5-CAGCGTGCAACTGTGGTACC-3

Amplicon was verified by sequencing

CGGGAACATGCATTACGATTCGAGCTCGGTACCCGGGGATCCTCTAGAGATTCAGCGTGCAACTGTGGTACCGTGCTAGTCGTATCCAGTGCGAATACCTCGGACCCTGCACAATCGTCGACCTGCAGGCATGCAAGCTTGGCACTGGCCGTCGTTTTACAACGTCGTGACTGGGAAAACCCTGGCGTTACCCAACTTAATCGCCTTGCAGCACATCCCCCTTTCGCCAGCTGGCGTAATAGCGAAGAGGCCCGCACCGATCGCCCTTCCCAACAGTTGCGCAGCCTGAATGGCGAATGGCGCCTGATGCGGTATTTTCTCCTTACGCATCTGTGCGGTATTTCACACCGCATATGGTGCACTCTCAGTACAATCTGCTCTGATGCCGCATAGTTAAGCCAGCCCCGACACCCGCCAACACCCGCTGACGCGCCCTGACGGGCTTGTCTGCTCCCGGCATCCGCTTACAGACAAGCTGTGACCGTCTCCGGGAGCTGCATGTGTCAGAGGTTTTCACCGTCATCACCGAAACGCGCGAGACGAAAGGGCCTCGTGATACGCCTATTTTTATAGGTTAATGTCATGATAATAATGGTTTCTTAGACGTCAGGTGGCACTTTTCGGGGAAATGTGCGCGGAACCCCTATTTGTTTATTTTTCTAAATACATTCAAATATGTATCCGCTCATGAGACAATAACCCTGATAAATGCTTCAATAATATTGAAAAAGGAAGAGTATGAGTATTCAACATTTCCGTGTCGCCCTTATTCCCTTTTTTGCGGCATTTTGCCTTCCTGTTTTTGCTCACCCAGAAACGCTGGTGAAAGTAAAAGATGCTGAAGATCAGTTGGGTGCACGAGTGGGTTACATCGAACTGGATCTCAACAGCGGTAAGATCCTTGAGAGTTTTCGCCCCGAAGAACGTTTTCCAATGATGAGCACTTTTAAAGTTCTGCTATGTGGCGCGGTATTATCCCGTATTGACGCCGGGCAAGAGCAACTCGGTCGCCGCATACACTATTCTCAGATGACTTGGTTGAGTACTCACCAGTCACAGAAAGCATCTTACGGATGGCATGACAGTAAGAGATTATGCAGTGCTGCCATAACCATGAGTGATAAACACTGCCGCACCTACTTCTGACACGATCGGAGGACGAGGAGCTACGCTTTTTGCACAACATGGGGATCATTACTCGCCTTTATCGTTGGCACCGACCTGAATGAAGCCATCCAAACGACAGAGCGTGTACACCACAGAGTGTCCTGTTTACACAATGATGGCACACACACCACG

NOVEL40 5-TGATTGAGCCGTGCCAATATC-3

RT-primer：5-GTCGTATCCAGTGCAGGGTCCGAGGTATTCGCACTGGATACGACGATATT-3

Forward primer： 5-CAGCGTGATTGAGCCGTGCC-3

Amplicon was verified by sequencing

GTTGGAACATTGATTACGATTCGAGCTCGGTACCCGGGGATCCTCTAGAGATTCAGCGTGATTGAGCCGTGCCAATATCGTCGTATCCAGTGCGAATACCTCGGACCCTGCACAATCGTCGACCTGCAGGCATGCAAGCTTGGCACTGGCCGTCGTTTTACAACGTCGTGACTGGGAAAACCCTGGCGTTACCCAACTTAATCGCCTTGCAGCACATCCCCCTTTCGCCAGCTGGCGTAATAGCGAAGAGGCCCGCACCGATCGCCCTTCCCAACAGTTGCGCAGCCTGAATGGCGAATGGCGCCTGATGCGGTATTTTCTCCTTACGCATCTGTGCGGTATTTCACACCGCATATGGTGCACTCTCAGTACAATCTGCTCTGATGCCGCATAGTTAAGCCAGCCCCGACACCCGCCAACACCCGCTGACGCGCCCTGACGGGCTTGTCTGCTCCCGGCATCCGCTTACAGACAAGCTGTGACCGTCTCCGGGAGCTGCATGTGTCAGAGGTTTTCACCGTCATCACCGAAACGCGCGAGACGAAAGGGCCTCGTGATACGCCTATTTTTATAGGTTAATGTCATGATAATAATGGTTTCTTAGACGTCAGGTGGCACTTTTCGGGGAAATGTGCGCGGAACCCCTATTTGTTTATTTTTCTAAATACATTCAAATATGTATCCGCTCATGAGACAATAACCCTGATAAATGCTTCAATAATATTGAAAAAGGAAGAGTATGAGTATTCAACATTTCCGTGTCGCCCTTATTCCCTTTTTTGCGGCATTTTGCCTTCCTGTTTTTGCTCACCCAGAAACGCTGGTGAAAGTAAAAGATGCTGAAGATCAGTTGGGTGCACGAGTGGGTTACATCGAACTGGATCTCACAGCGGTAAGATCCTTGAGAGTTTTCGCCCCGAAGAACGTTTTCCCATGATGAGCACTTTTAAAGTTCTGCTATGTGCGCGGTATTATCCGTATTGACGCCGGGCAAGAGCACTCGGTCGCGCATACACTATTCTCAGATGACTTGTTGAGTACTCACAGTCACAGAAAAGCATCTACGATGCATGACAGTAGAGATTATGCATGCTGCATAACCATGATGATACACTGCGCACTACTCTGACACGATCGGAGGACGAGACTACCGCTTTTGCACAACATGGGATCATGTACTCGCCTTGATCGTGGACGGACTGATGACTACAAGCAGCGACGTGACCCAGCATTCCTGGTTACATGGCACTACGAC

NOVEL41 5-TCATGCGATCCCTTCGGAATT-3

RT-primer：5-GTCGTATCCAGTGCAGGGTCCGAGGTATTCGCACTGGATACGACAATTCC-3

Forward primer： 5-CATAGTCATGCGATCCCTTC-3

Amplicon was verified by sequencing

TGGACATGGATTACGAATTCGAGCTCGGTACCCGGGGATCCTCTAGAGATTCATAGTCATGCGATCCCTTCGGAATTGTCGTATCCAGTGCGAATACCTCGGACCCTGCACAATCGTCGACCTGCAGGCATGCAAGCTTGGCACTGGCCGTCGTTTTACAACGTCGTGACTGGGAAAACCCTGGCGTTACCCAACTTAATCGCCTTGCAGCACATCCCCCTTTCGCCAGCTGGCGTAATAGCGAAGAGGCCCGCACCGATCGCCCTTCCCAACAGTTGCGCAGCCTGAATGGCGAATGGCGCCTGATGCGGTATTTTCTCCTTACGCATCTGTGCGGTATTTCACACCGCATATGGTGCACTCTCAGTACAATCTGCTCTGATGCCGCATAGTTAAGCCAGCCCCGACACCCGCCAACACCCGCTGACGCGCCCTGACGGGCTTGTCTGCTCCCGGCATCCGCTTACAGACAAGCTGTGACCGTCTCCGGGAGCTGCATGTGTCAGAGGTTTTCACCGTCATCACCGAAACGCGCGAGACGAAAGGGCCTCGTGATACGCCTATTTTTATAGGTTAATGTCATGATAATAATGGTTTCTTAGACGTCAGGTGGCACTTTTCGGGGAAATGTGCGCGGAACCCCTATTTGTTTATTTTTCTAAATACATTCAAATATGTATCCGCTCATGAGACAATAACCCTGATAAATGCTTCAATAATATTGAAAAAGGAAGAGTATGAGTATTCAACATTTCCGTGTCGCCCTTATTCCCTTTTTTGCGGCATTTTGCCTTCCTGTTTTTGCTCACCCAGAAACGCTGGTGAAAGTAAAAGATGCTGAAGATCAGTTGGGTGCACGAGTGGGTTACATCGAACTGGATCTCAACAGCGGTAAGATCCTTGAGAGTTTTCGCCCCGAAGAACGTTTTCCAATGATGAGCACTTTTAAAGTTCTGCTATGTGGCGCGGTATTATCCCGTATTGACGCCGGGCAAGAGCAACTCGGTCGCGGCATACACTATTCTCAGATGACTTGGTTGAGTACTCACCAGTCACAGAAAGCATCTTACGGATGGCATGACAGTAGAGAATTATGCAGTGCTGCCATTACCATGGAGTGATACACTGCGCTACTTACTTCTGACACGATCGGAGACGATGAGCTACCGCTTTTTGCCACATGGGGATCATGTACTCGCCTGTATCGTGGACCGGACTGATGGAAGCCTATCAATCGACGAAGCGGTGAACACCACGGAAGTGCCTGTTTTGCC

NOVEL42 5-TGGAAAATAATGATCGTAGAA-3

RT-primer：5-GTCGTATCCAGTGCAGGGTCCGAGGTATTCGCACTGGATACGACTTCTAC-3

Forward primer： 5-CAGCCTGGAAAATAATGATC-3

Amplicon was verified by sequencing

AGAACATGAACATGATTACGATTCGAGCTCGGTACCCGGGGATCCTCTAGAGATTCAGCCTGGAAAATAATGATCGTAGAAGTCGTATCCAGTGCGAATACCTCGGACCCTGCACAATCGTCGACCTGCAGGCATGCAAGCTTGGCACTGGCCGTCGTTTTACAACGTCGTGACTGGGAAAACCCTGGCGTTACCCAACTTAATCGCCTTGCAGCACATCCCCCTTTCGCCAGCTGGCGTAATAGCGAAGAGGCCCGCACCGATCGCCCTTCCCAACAGTTGCGCAGCCTGAATGGCGAATGGCGCCTGATGCGGTATTTTCTCCTTACGCATCTGTGCGGTATTTCACACCGCATATGGTGCACTCTCAGTACAATCTGCTCTGATGCCGCATAGTTAAGCCAGCCCCGACACCCGCCAACACCCGCTGACGCGCCCTGACGGGCTTGTCTGCTCCCGGCATCCGCTTACAGACAAGCTGTGACCGTCTCCGGGAGCTGCATGTGTCAGAGGTTTTCACCGTCATCACCGAAACGCGCGAGACGAAAGGGCCTCGTGATACGCCTATTTTTATAGGTTAATGTCATGATAATAATGGTTTCTTAGACGTCAGGTGGCACTTTTCGGGGAAATGTGCGCGGAACCCCTATTTGTTTATTTTTCTAAATACATTCAAATATGTATCCGCTCATGAGACAATAACCCTGATAAATGCTTCAATAATATTGAAAAAGGAAGAGTATGAGTATTCAACATTTCCGTGTCGCCCTTATTCCCTTTTTTGCGGCATTTTGCCTTCCTGTTTTTGCTCACCCAGAAACGCTGGTGAAAGTAAAAGATGCTGAAGATCAGTTGGGTGCACGAGTGGGTTACATCGAACTGGATCTCAACAGCGGTAAGATCCTTGAGAGTTTTCGCCCCGAAGAACGTTTTCCAATGATGAGCACTTTTAAAGTTCTGCTATGTGGCGCGGTATTATCCCGTATTGACGCCGGGCAAGAGCAACTCGGTCGCCGCATACACTATTCTCAGAATGACTTGGTTGAGTACTCACCAGTCACAGAAAAGCATCTACGGATGGCATGACAGTAGAGATATGCAGTGCTGCATACATGGAGTGATACCACTGGCGGCCACCTACCTCCTGACACGATCGGAGGACGAACGAGCCTACGCTTTTTGCACAACATGGGGATCATGTAACTCGCCATGAATCGTTGGGATCGGGAGCTGTAATGTGAAGGCCCTTATCCAAAGCGC

NOVEL43 5-AGGTGCAGCTGTGGTATGGTA-3

RT-primer：5-GTCGTATCCAGTGCAGGGTCCGAGGTATTCGCACTGGATACGACTACCAT-3

Forward primer： 5-CGGCGAGGTGCAGCTGTGGT-3

Amplicon was verified by sequencing

TCAAGTCGTTTTGGATATTGGCTACAGTCCAGCTTCAGGTTCCGATCGATTCAAGTCAGTTACAGATCGCACTGCTGTGCATGACGCGTTAGGCTCATGCGTCCTTCGCTTCGTGATCAGTAGTCAAGTTGGACGTCAGTGTCATGCATCATAGTCATGCTAGCAGCTAGCATAATCATATACTGTCATGGCCATCGCTAAGATGATGTTGCTGTGACTGGTGAGTACTCAAACAAGTCATGTGAGAATAGGATATGCGCGACCGAGTTGCTCTTGCTCGGCGTCAATACGAGTTAATACCGCGCCACATAGCAGAACTTTAAAAGTGATCATCATTGGAAAACGATAATCGTGGCGAAAACTCTAAAGGATATGACTGCTGTTGAGATCCAGTTCGATGTAACCCACTCGTGCACCCACATGATCTTCAGCATCTGTTACTTTCACCAGCGTTTGTGTGTGAGCAAAAACAGGAAGGCAAAATGCCGCAAAAAAGAGAATAAGGGTGACACGGAAATGTTGAATACTCATACTCTTCCTTTTTCAATATTATTGAAGCATTTATCAGGGTTATTGTCTCATGAGCGGATACATATTCGAATGTATTTAGAAAAATAAACAAATAGGGGTTCCGCGCACATTTCCCCGAAAAGTGCCACCTGACGTCTAAGAAACCATTATCATCATGACATTAACCTATAAAAATAGGAGTATCACGAGGCCCTTTCGTCTCGCGCGTTTCGGTGATGACGGTGAAAACGTATGACTCATGCAGCTCCCGGAGACGGTCACAGTTTGTCTGTAAGCGGATGCCGGGAGCAGACAAGCCCGTCAGGGCGCGTCAGCGGGTGTTGGCGGGTGTCGGGGCTGGGTTAAGTATGCGGCATCAGAGCAGATTGTAATGAGAGTGCACCATCTGCGGTGTGAAATACCGCACAGATGCGTAAGGAGAAAATAAAGCATCAGGCTCCATTCGCCATTCAGGTTGCGCAAGTGTTGGGAAGGGCGATCGGTGCGGGCCTGTTCGTTATTACGCCAGCTGGCGAAAGGGGGATGTGGTGCAAGGCGATTAAGTTGGGTAACGGCAGGGTTTTCCCAGTCACGACGTTGTAAAACGACGGCCAGTGCCAAGCTCGCATGCCCGCAGGTGGAGGATTCGGCGAGGTGCAGCTGTGGTATGGTAGTCGTATCCAGTGCGAATACCTCGGACCCTGCACAATCTCTAGAGGATCCCCGGGTACCGAGCTCGAATCGTAATCATGTAAAATTTT

NOVEL44 5-GAATCTTGATGATGCTGCAT-3

RT-primer：5-GTCGTATCCAGTGCAGGGTCCGAGGTATTCGCACTGGATACGACATGCAG-3

Forward primer： 5-CAGCGGAATCTTGATGATG-3

Amplicon was verified by sequencing

TCACATTTGATACACACCAGCATTTCGTGATGTTCACCGCCTCGCTCGTTGGTTGGCTTCATTCAGGTCCGGTTCCAACGATAAGGCGAGTACATGATCCCCCATGTTGTGCAAAAAGCGTAGCTCGTCGTCTCCGATCGTGTCAGAAGTAGTGCGCAGTGTATCACTCATGGTATGGCAGCACTGCATAATTCTCTACTGTCATGCCATCCGTAAGATGCTTTCTGTGACTGGTGAGTACTCAACCAAGTCATCTGAGAATAGTGTATGCGGCGACCGAGTTGCTCTTGCCCGGCGTCAATACGGGATAATACCGCGCCACATAGCAGAACTTTAAAAGTGCTCATCATTGGAAAACGTTCTTCGGGGCGAAAACTCTCAAGGATCTTACCGCTGTTGAGATCCAGTTCGATGTAACCCACTCGTGCACCCAACTGATCTTCAGCATCTTTTACTTTCACCAGCGTTTCTGGGTGAGCAAAAACAGGAAGGCAAAATGCCGCAAAAAAGGGAATAAGGGCGACACGGAAATGTTGAATACTCATACTCTTCCTTTTTCAATATTATTGAAGCATTTATCAGGGTTATTGTCTCATGAGCGGATACATATTTGAATGTATTTAGAAAAATAAACAAATAGGGGTTCCGCGCACATTTCCCCGAAAAGTGCCACCTGACGTCTAAGAAACCATTATTATCATGACATTAACCTATAAAAATAGGCGTATCACGAGGCCCTTTCGTCTCGCGCGTTTCGGTGATGACGGTGAAAACCTCTGACACATGCAGCTCCCGGAGACGGTCACAGCTTGTCTGTAAGCGGATGCCGGGAGCAGACAAGCCCGTCAGGGCGCGTCAGCGGGTGTTGGCGGGTGTCGGGGCTGGCTTAACTATGCGGCATCAGAGCAGATTGTACTGAGAGTGCACCATATGCGGTGTGAAATACCGCACAGATGCGTAAGGAGAAAATACCGCATCAGGCGCCATTCGCCATTCAGGCTGCGCAACTGTTGGGAAGGGCGATCGGTGCGGGCCTCTTCGCTATTACGCCAGCTGGCGAAAGGGGGATGTGCTGCAAGGCGATTAAGTTGGGTAACGCCAGGGTTTTCCCAGTCACGACGTTGTAAAACGACGGCCAGTGCCAAGCTTGCATGCCTGCAGGTCGACGATTCAGCGGAATCTTGATGATGCTGCATGTCGTATCCAGTGCGAATACCTCGGACCCTGCACAATCTCTAGAGGATCCCCGGGTACCGAGCTCGAATTCGTAATCATGTTCAATTGTC

NOVEL45 5-AGAATCTTGATGATGCTGCAT-3

RT-primer：5-GTCGTATCCAGTGCAGGGTCCGAGGTATTCGCACTGGATACGACATGCAG-3

Forward primer： 5-CAGCGAGAATCTTGATGATG-3

Amplicon was verified by sequencing

GTGTACACCGCTTTGTCGTTGTAGGCTCATCAGTTCCGTTTCACGGATCCAGGCAGGTTACATATTCCCCCCATGTTGTGCAAAAAGCGTAGCTCCTCGTCCTCCGATCGTGTCAGAAGTAAGTGCGCAGTGTATCATCATGTATGCAGCACTGCATAATCTCTACTGTCATGCCATCCGTAAGATGCTTTTCTGTGACTGGTGAGTACTCAACCAAGTCATTCTGAGAATAGTGTATGCGGCGACCGAGTTGCTCTTGCCCGGCGTCAATACGGGATAATACCGCGCCACATAGCAGAACTTTAAAAGTGCTCATCATTGGAAAACGTTCTTCGGGGCGAAAACTCTCAAGGATCTTACCGCTGTTGAGATCCAGTTCGATGTAACCCACTCGTGCACCCAACTGATCTTCAGCATCTTTTACTTTCACCAGCGTTTCTGGGTGAGCAAAAACAGGAAGGCAAAATGCCGCAAAAAAGGGAATAAGGGCGACACGGAAATGTTGAATACTCATACTCTTCCTTTTTCAATATTATTGAAGCATTTATCAGGGTTATTGTCTCATGAGCGGATACATATTTGAATGTATTTAGAAAAATAAACAAATAGGGGTTCCGCGCACATTTCCCCGAAAAGTGCCACCTGACGTCTAAGAAACCATTATTATCATGACATTAACCTATAAAAATAGGCGTATCACGAGGCCCTTTCGTCTCGCGCGTTTCGGTGATGACGGTGAAAACCTCTGACACATGCAGCTCCCGGAGACGGTCACAGCTTGTCTGTAAGCGGATGCCGGGAGCAGACAAGCCCGTCAGGGCGCGTCAGCGGGTGTTGGCGGGTGTCGGGGCTGGCTTAACTATGCGGCATCAGAGCAGATTGTACTGAGAGTGCACCATATGCGGTGTGAAATACCGCACAGATGCGTAAGGAGAAAATACCGCATCAGGCGCCATTCGCCATTCAGGCTGCGCAACTGTTGGGAAGGGCGATCGGTGCGGGCCTCTTCGCTATTACGCCAGCTGGCGAAAGGGGGATGTGCTGCAAGGCGATTAAGTTGGGTAACGCCAGGGTTTTCCCAGTCACGACGTTGTAAAACGACGGCCAGTGCCAAGCTTGCATGCCTGCAGGTCGACGATTCAGCGAGAATCTTGATGATGCTGCATGTCGTATCCAGTGCGAATACCTCGGACCCTGCACAATCTCTAGAGGATCCCCGGGTACCGAGCTCGAATCGTAATCATGTCAATGTTGT

NOVEL46 5-TTGGACCAGGCTTCATTCCAC-3

RT-primer：5-GTCGTATCCAGTGCAGGGTCCGAGGTATTCGCACTGGATACGACGTGGAA-3

Forward primer： 5-CAGCGTTGGACCAGGCTTCA-3

Amplicon was verified by sequencing

CACGCGGCACTCGGTGGTACCGCTCGTCGTTGATGCTCATAAGTCCGTCGACGATCAAGCGAGTTACATGATCCCCATGTTGGCAAAAAGCGGTAGCTCCTCCGTCTTCGATCGTGTCAGAAGTAAGTTGCGCAGTGTTATCACTCATGGTATGGCAGCACTGCATAATCTCTACTGTCATGCCATCCGTAAGATGCTTTTCTGTGACTGGTGAGTACTCAACCAAGTCATCTGAGAATAGTGTATGCGGCGACCGAGTTGCTCTTGCCCGGCGTCAATACGGGATAATACCGCGCCACATAGCAGAACTTTAAAAGTGCTCATCATTGGAAAACGTTCTTCGGGGCGAAAACTCTCAAGGATCTTACCGCTGTTGAGATCCAGTTCGATGTAACCCACTCGTGCACCCAACTGATCTTCAGCATCTTTTACTTTCACCAGCGTTTCTGGGTGAGCAAAAACAGGAAGGCAAAATGCCGCAAAAAAGGGAATAAGGGCGACACGGAAATGTTGAATACTCATACTCTTCCTTTTTCAATATTATTGAAGCATTTATCAGGGTTATTGTCTCATGAGCGGATACATATTTGAATGTATTTAGAAAAATAAACAAATAGGGGTTCCGCGCACATTTCCCCGAAAAGTGCCACCTGACGTCTAAGAAACCATTATTATCATGACATTAACCTATAAAAATAGGCGTATCACGAGGCCCTTTCGTCTCGCGCGTTTCGGTGATGACGGTGAAAACCTCTGACACATGCAGCTCCCGGAGACGGTCACAGCTTGTCTGTAAGCGGATGCCGGGAGCAGACAAGCCCGTCAGGGCGCGTCAGCGGGTGTTGGCGGGTGTCGGGGCTGGCTTAACTATGCGGCATCAGAGCAGATTGTACTGAGAGTGCACCATATGCGGTGTGAAATACCGCACAGATGCGTAAGGAGAAAATACCGCATCAGGCGCCATTCGCCATTCAGGCTGCGCAACTGTTGGGAAGGGCGATCGGTGCGGGCCTCTTCGCTATTACGCCAGCTGGCGAAAGGGGGATGTGCTGCAAGGCGATTAAGTTGGGTAACGCCAGGGTTTTCCCAGTCACGACGTTGTAAAACGACGGCCAGTGCCAAGCTTGCATGCCTGCAGGTCGACGATTCAGCGTTGGACCAGGCTTCATTCCACGTCGTATCCAGTGCGAATACCTCGGACCCTGCACAATCTCTAGAGGATCCCCGGGTACCGAGCTCGAATCGTAAACATGGTCCATT

NOVEL47 5-TTTAAAATCACACGGCTTTAA-3

RT-primer：5-GTCGTATCCAGTGCAGGGTCCGAGGTATTCGCACTGGATACGACTTAAAG-3

Forward primer： 5-CGCCGTTTAAAATCACACGG-3

NOVEL48 5-CAATCGATGGATTGGATATGGA-3

RT-primer：5-GTCGTATCCAGTGCAGGGTCCGAGGTATTCGCACTGGATACGACTCCATA-3

Forward primer： 5-CATCGCAATCGATGGATTGGA-3

NOVEL49 5-ACCCAAATCACACAATCAAAGTTT-3

RT-primer：5-GTCGTATCCAGTGCAGGGTCCGAGGTATTCGCACTGGATACGACAAACTT-3

Forward primer： 5-CATCGACCCAAATCACACAATCA-3

NOVEL50 5-TCTCATGTCGCCCCTGCGGGA-3

RT-primer：5-GTCGTATCCAGTGCAGGGTCCGAGGTATTCGCACTGGATACGACTCCCGC-3

Forward primer： 5-CAGCGTCTCATGTCGCCCCT-3

Amplicon was verified by sequencing

GTGTACACCGCTTTGTCGTTGTAGGCTCATCAGTTCCGTTTCACGGATCCAGGCAGGTTACATATTCCCCCCATGTTGTGCAAAAAGCGTAGCTCCTCGTCCTCCGATCGTGTCAGAAGTAAGTGCGCAGTGTATCATCATGTATGCAGCACTGCATAATCTCTACTGTCATGCCATCCGTAAGATGCTTTTCTGTGACTGGTGAGTACTCAACCAAGTCATTCTGAGAATAGTGTATGCGGCGACCGAGTTGCTCTTGCCCGGCGTCAATACGGGATAATACCGCGCCACATAGCAGAACTTTAAAAGTGCTCATCATTGGAAAACGTTCTTCGGGGCGAAAACTCTCAAGGATCTTACCGCTGTTGAGATCCAGTTCGATGTAACCCACTCGTGCACCCAACTGATCTTCAGCATCTTTTACTTTCACCAGCGTTTCTGGGTGAGCAAAAACAGGAAGGCAAAATGCCGCAAAAAAGGGAATAAGGGCGACACGGAAATGTTGAATACTCATACTCTTCCTTTTTCAATATTATTGAAGCATTTATCAGGGTTATTGTCTCATGAGCGGATACATATTTGAATGTATTTAGAAAAATAAACAAATAGGGGTTCCGCGCACATTTCCCCGAAAAGTGCCACCTGACGTCTAAGAAACCATTATTATCATGACATTAACCTATAAAAATAGGCGTATCACGAGGCCCTTTCGTCTCGCGCGTTTCGGTGATGACGGTGAAAACCTCTGACACATGCAGCTCCCGGAGACGGTCACAGCTTGTCTGTAAGCGGATGCCGGGAGCAGACAAGCCCGTCAGGGCGCGTCAGCGGGTGTTGGCGGGTGTCGGGGCTGGCTTAACTATGCGGCATCAGAGCAGATTGTACTGAGAGTGCACCATATGCGGTGTGAAATACCGCACAGATGCGTAAGGAGAAAATACCGCATCAGGCGCCATTCGCCATTCAGGCTGCGCAACTGTTGGGAAGGGCGATCGGTGCGGGCCTCTTCGCTATTACGCCAGCTGGCGAAAGGGGGATGTGCTGCAAGGCGATTAAGTTGGGTAACGCCAGGGTTTTCCCAGTCACGACGTTGTAAAACGACGGCCAGTGCCAAGCTTGCATGCCTGCAGGTCGACGATTCAGCGTCTCATGTCGCCCCTGCGGGAGTCGTATCCAGTGCGAATACCTCGGACCCTGCACAATCTCTAGAGGATCCCCGGGTACCGAGCTCGAATCGTAATCATGTCAATGTTGT

NOVEL51 5-TGGAGAAGCAGGGCACGTGCAAA-3

RT-primer：5-GTCGTATCCAGTGCAGGGTCCGAGGTATTCGCACTGGATACGACTTTGCA-3

Forward primer： 5-AGCAATGGAGAAGCAGGGCACG-3

Amplicon was verified by sequencing

CTTCACATTCAAGCTCGATACCAACGATCAAGGCGAGTACAGATTCCAATGTTGCAAAAGCGTAGGTTCGTCCGTTCTTCCGATCGTGTCAGAGGTAGGTGCGGCAGTGTTATCATCATGTATGCAGCATGCATATTCTCTACTGTCATGCATCGTAGATGCTTTTCTGTGACTGGTGAGTACTCAACCAAGTCATCTGAGAATAGTGTATGCGGCGACCGAGTTGCTCTTGCCCGGCGTCAATACGGGATAATACCGCGCCACATAGCAGAACTTTAAAAGTGCTCATCATTGGAAAACGTTCTTCGGGGCGAAAACTCTCAAGGATCTTACCGCTGTTGAGATCCAGTTCGATGTAACCCACTCGTGCACCCAACTGATCTTCAGCATCTTTTACTTTCACCAGCGTTTCTGGGTGAGCAAAAACAGGAAGGCAAAATGCCGCAAAAAAGGGAATAAGGGCGACACGGAAATGTTGAATACTCATACTCTTCCTTTTTCAATATTATTGAAGCATTTATCAGGGTTATTGTCTCATGAGCGGATACATATTTGAATGTATTTAGAAAAATAAACAAATAGGGGTTCCGCGCACATTTCCCCGAAAAGTGCCACCTGACGTCTAAGAAACCATTATTATCATGACATTAACCTATAAAAATAGGCGTATCACGAGGCCCTTTCGTCTCGCGCGTTTCGGTGATGACGGTGAAAACCTCTGACACATGCAGCTCCCGGAGACGGTCACAGCTTGTCTGTAAGCGGATGCCGGGAGCAGACAAGCCCGTCAGGGCGCGTCAGCGGGTGTTGGCGGGTGTCGGGGCTGGCTTAACTATGCGGCATCAGAGCAGATTGTACTGAGAGTGCACCATATGCGGTGTGAAATACCGCACAGATGCGTAAGGAGAAAATACCGCATCAGGCGCCATTCGCCATTCAGGCTGCGCAACTGTTGGGAAGGGCGATCGGTGCGGGCCTCTTCGCTATTACGCCAGCTGGCGAAAGGGGGATGTGCTGCAAGGCGATTAAGTTGGGTAACGCCAGGGTTTTCCCAGTCACGACGTTGTAAAACGACGGCCAGTGCCAAGCTTGCATGCCTGCAGGTCGACGATTCAGCAAGGAGAAGCAGGGCACGTGCAAAGTCGTATCCAGTGCGAATACCTCGGACCCTGCACAATCTCTAGAGGATCCCCGGGTACCGAGCTCGAATCGTAATCATGTCATGTCGGT

NOVEL52 5-TCCCACAGCTTTATTGAACCGC-3

RT-primer：5-GTCGTATCCAGTGCAGGGTCCGAGGTATTCGCACTGGATACGACGCGGTT-3

Forward primer： 5-CAGCGTCCCACAGCTTTATTG-3

Amplicon was verified by sequencing

AGACAAATGACCATGATTACGATTCGAGCTCGGTACCCGGGGATCCTCTAGAGATTCAGCGTCCCACAGCTTTATTGACCGCGTCGTATCCAGTGCGAATACCTCGGACCCTGCACAATCGTCGACCTGCAGGCATGCAAGCTTGGCACTGGCCGTCGTTTTACAACGTCGTGACTGGGAAAACCCTGGCGTTACCCAACTTAATCGCCTTGCAGCACATCCCCCTTTCGCCAGCTGGCGTAATAGCGAAGAGGCCCGCACCGATCGCCCTTCCCAACAGTTGCGCAGCCTGAATGGCGAATGGCGCCTGATGCGGTATTTTCTCCTTACGCATCTGTGCGGTATTTCACACCGCATATGGTGCACTCTCAGTACAATCTGCTCTGATGCCGCATAGTTAAGCCAGCCCCGACACCCGCCAACACCCGCTGACGCGCCCTGACGGGCTTGTCTGCTCCCGGCATCCGCTTACAGACAAGCTGTGACCGTCTCCGGGAGCTGCATGTGTCAGAGGTTTTCACCGTCATCACCGAAACGCGCGAGACGAAAGGGCCTCGTGATACGCCTATTTTTATAGGTTAATGTCATGATAATAATGGTTTCTTAGACGTCAGGTGGCACTTTTCGGGGAAATGTGCGCGGAACCCCTATTTGTTTATTTTTCTAAATACATTCAAATATGTATCCGCTCATGAGACAATAACCCTGATAAATGCTTCAATAATATTGAAAAAGGAAGAGTATGAGTATTCAACATTTCCGTGTCGCCCTTATTCCCTTTTTTGCGGCATTTTGCCTTCCTGTTTTTGCTCACCCAGAAACGCTGGTGAAAGTAAAAGATGCTGAAGATCAGTTGGGTGCACGAGTGGGTTACATCGAACTGGATCTCAACAGCGGTAAGATCCTTGAGAGTTTTCGCCCCGAAGAACGTTTTCCAATGATGAGCACTTTTAAAGTTCTGCTATGTGGCGCGGTATTATCCCGTATTGACGCCGGGCAAGAGCAACTCGGTCGCCGCATACACTATTCTCAGAATGACTTGGGTTGAGTACTCACCAGTCACAGAAAAGCATCTTACGGATGGCATGACAGTAGGAGGATTATGCAATGCTGCATAACCATGGAGTGGATAAACACCTGCCGCACTACTCTGACACGATCGAGGACCGGAAGGACCTACCGCTTTGGCACAACATGGGATCAGGTATTGGCTTGGATCGTGGACCGGGACCTGTATGAGCCTCATACTCAGA

NOVEL53 5-TTGAGCCGCGCCAATATCACT-3

RT-primer：5-GTCGTATCCAGTGCAGGGTCCGAGGTATTCGCACTGGATACGACAGTGAT-3

Forward primer： 5-CAGCGTTGAGCCGCGCCAAT-3

Amplicon was verified by sequencing

CGTTTAGGATTATTGGCCTTCATTTCAGCCCCGGGTCCAACGATCAGGGCGAGTAACTGATTCCCCATGTTTGTGCAAAAAAGCCGTAGCTCCTCGGTCTCGATCGTTGTCAGAAGTAGTTGCCGCCAGTGTATCACTCATGATATGCAGCACTGCATAATCTCTACTGTCATGCCATCCGTAAGATGCTTTTCTGTGACTGGTGAGTACTCAACCAAGTCATTCTGAGAATAGTGTATGCGGGCGACCGAGTTGCTCTTGCCCGGGCGTCAATACGGGATAATACCGCGCCACATAGCAGAACTTTAAAAGTGCTCATCATTGGAAAACGTTCTTCGGGGCGAAAACTCTCAAGGATCTTACCGCTGTTGAGATCCAGTTCGATGTAACCCACTCGTGCACCCAACTGATCTTCAGCATCTTTTACTTTCACCAGCGTTTCTGGGTGAGCAAAAACAGGAAGGCAAAATGCCGCAAAAAAGGGAATAAGGGCGACACGGAAATGTTGAATACTCATACTCTTCCTTTTTCAATATTATTGAAGCATTTATCAGGGTTATTGTCTCATGAGCGGATACATATTTGAATGTATTTAGAAAAATAAACAAATAGGGGTTCCGCGCACATTTCCCCGAAAAGTGCCACCTGACGTCTAAGAAACCATTATTATCATGACATTAACCTATAAAAATAGGCGTATCACGAGGCCCTTTCGTCTCGCGCGTTTCGGTGATGACGGTGAAAACCTCTGACACATGCAGCTCCCGGAGACGGTCACAGCTTGTCTGTAAGCGGATGCCGGGAGCAGACAAGCCCGTCAGGGCGCGTCAGCGGGTGTTGGCGGGTGTCGGGGCTGGCTTAACTATGCGGCATCAGAGCAGATTGTACTGAGAGTGCACCATATGCGGTGTGAAATACCGCACAGATGCGTAAGGAGAAAATACCGCATCAGGCGCCATTCGCCATTCAGGCTGCGCAACTGTTGGGAAGGGCGATCGGTGCGGGCCTCTTCGCTATTACGCCAGCTGGCGAAAGGGGGATGTGCTGCAAGGCGATTAAGTTGGGTAACGCCAGGGTTTTCCCAGTCACGACGTTGTAAAACGACGGCCAGTGCCAAGCTTGCATGCCTGCAGGTCGACGATTCAGCGTTGAGCCGCGCCAATATCACTGTCGTATCCAGTGCGAATACCTCGGACCCTGCACAATCTCTAGAGGATCCCCGGGTACCGAGCTCGAATCGTAATCATGTCATGTTCCT

NOVEL54 5-GCTCACTCTCTATCTGTCACC-3

RT-primer：5-GTCGTATCCAGTGCAGGGTCCGAGGTATTCGCACTGGATACGACGGTGAC-3

Forward primer： 5-CATAGGCTCACTCTCTATCT-3

Amplicon was verified by sequencing

CCGGACAATGACTTGATTACGATTCGAGCTCGGTACCCGGGGATCCTCTAGAGATTCATAGGCTCACTCTCTATCTGTCACCGTCGTATCCAGTGCGAATACCTCGGACCCTGCACAATCGTCGACCTGCAGGCATGCAAGCTTGGCACTGGCCGTCGTTTTACAACGTCGTGACTGGGAAAACCCTGGCGTTACCCAACTTAATCGCCTTGCAGCACATCCCCCTTTCGCCAGCTGGCGTAATAGCGAAGAGGCCCGCACCGATCGCCCTTCCCAACAGTTGCGCAGCCTGAATGGCGAATGGCGCCTGATGCGGTATTTTCTCCTTACGCATCTGTGCGGTATTTCACACCGCATATGGTGCACTCTCAGTACAATCTGCTCTGATGCCGCATAGTTAAGCCAGCCCCGACACCCGCCAACACCCGCTGACGCGCCCTGACGGGCTTGTCTGCTCCCGGCATCCGCTTACAGACAAGCTGTGACCGTCTCCGGGAGCTGCATGTGTCAGAGGTTTTCACCGTCATCACCGAAACGCGCGAGACGAAAGGGCCTCGTGATACGCCTATTTTTATAGGTTAATGTCATGATAATAATGGTTTCTTAGACGTCAGGTGGCACTTTTCGGGGAAATGTGCGCGGAACCCCTATTTGTTTATTTTTCTAAATACATTCAAATATGTATCCGCTCATGAGACAATAACCCTGATAAATGCTTCAATAATATTGAAAAAGGAAGAGTATGAGTATTCAACATTTCCGTGTCGCCCTTATTCCCTTTTTTGCGGCATTTTGCCTTCCTGTTTTTGCTCACCCAGAAACGCTGGTGAAAGTAAAAGATGCTGAAGATCAGTTGGGTGCACGAGTGGGTTACATCGAACTGGATCTCAACAGCGGTAAGATCCTTGAGAGTTTTCGCCCCGAAGAACGTTTTCCAATGATGAGCACTTTTAAAGTTCTGCTATGTGGCGCGGTATTATCCCGTATTGACGCCGGGCAAGAGCAACTCGGTCGCCGCATACACTATTCTCAGAATGACTTGGGTTGAGTACTCACCAGTCACAGAAAAGCATCTTACGGGATGGCATGACAGTAGAGAATTATGCAGTGCTGCATACATGAGTGATAACACTGGCGGCACTACTTCTGACACGATCGGAGGACGAAGGACCTACGCTTTTGCCCACATGGGGGATCATGTACTGCCTGGATCGTGGGACGGACTGAATGAGCCATACTAACGAACGAAGGCGGGT

NOVEL55 5-ATAGTCTGAAGTAGAAGATAGTT-3

RT-primer：5-GTCGTATCCAGTGCAGGGTCCGAGGTATTCGCACTGGATACGACAACTAT-3

Forward primer： 5-CGCCGATAGTCTGAAGTAGAAG -3

Amplicon was verified by sequencing

AGAACATTGACTGATTACGATTCGAGCTCGGTACCCGGGGATCCTCTAGAGATTCCGCCGTAGTCTGAAGTAGAAGCATAGTTGTCGTATCCAGTGCGAATACCTCGGACCCTGCACAATCGTCGACCTGCAGGCATGCAAGCTTGGCACTGGCCGTCGTTTTACAACGTCGTGACTGGGAAAACCCTGGCGTTACCCAACTTAATCGCCTTGCAGCACATCCCCCTTTCGCCAGCTGGCGTAATAGCGAAGAGGCCCGCACCGATCGCCCTTCCCAACAGTTGCGCAGCCTGAATGGCGAATGGCGCCTGATGCGGTATTTTCTCCTTACGCATCTGTGCGGTATTTCACACCGCATATGGTGCACTCTCAGTACAATCTGCTCTGATGCCGCATAGTTAAGCCAGCCCCGACACCCGCCAACACCCGCTGACGCGCCCTGACGGGCTTGTCTGCTCCCGGCATCCGCTTACAGACAAGCTGTGACCGTCTCCGGGAGCTGCATGTGTCAGAGGTTTTCACCGTCATCACCGAAACGCGCGAGACGAAGGGGCCTCGTGATACGCCTATTTTTATAGGTTAATGTCATGATAATAATGGTTTCTTAGACGTCGGGTGGCACTTTTCGGGGAAATGTGCGCGGAACCCCTATTTGTTTATTTTTCTAAATACATTCAAATATGTATCCGCTCATGAGACAATAACCCTGATAAATGCTTCAATAATATTGAAAAAGGAAGAGTATGAGTATTCAACATTTCCGTGTCGCCCTTATTCCCTTTTTTGCGGCATTTTGCCTTCGTGTTTTTGCTCACCCAGAAACGCTGGTGAAAGTAAAAGATGCTGAAGATCAGTTGGGTGCACGAGTGGGTTACATCGAACTGGATCTCAACAGCGGTAAGATCCTTGAGAGTTTTCGCCCCGAACACGTTTTCCAATGATGAGCACTTTTAAAGTTCTGCTATGTGGCGCGGTATATCCCGTAGTGACGCCGGGACAGAGCAACTCGGTCGCGCATACACTATTCTCAGAATGACGTGGTGAGTACTCACAGTCACAGACAGTCATCTACGGATGGCATGACAGTAGAGATGATGCAGTGCTGCATACCATGAGTGATACTCTGCCGCACTAACTTCTGACACGATCGGAGACCGAGAACTTACGCACTGGCAACTGGGAATCGGTAACTCGCTGAATCGTGGAACCGAGCTGAATGAG

NOVEL56 5-TTGACGGAAGATAGAGAGCAC-3

RT-primer：5-GTCGTATCCAGTGCAGGGTCCGAGGTATTCGCACTGGATACGACGTGCTC-3

Forward primer： 5-CAGCGTTGACGGAAGATAGA-3

Amplicon was verified by sequencing

AATTAACCTGAATTACCGATTCGAGCTCGGTACCCGGGGATCCTCTAGAGATTCAGCGTTGACGGAAGATAGAGAGCACGTCGTATCCAGTGCGAATACCTCGGACCCTGCACAATCGTCGACCTGCAGGCATGCAAGCTTGGCACTGGCCGTCGTTTTACAACGTCGTGACTGGGAAAACCCTGGCGTTACCCAACTTAATCGCCTTGCAGCACATCCCCCTTTCGCCAGCTGGCGTAATAGCGAAGAGGCCCGCACCGATCGCCCTTCCCAACAGTTGCGCAGCCTGAATGGCGAATGGCGCCTGATGCGGTATTTTCTCCTTACGCATCTGTGCGGTATTTCACACCGCATATGGTGCACTCTCAGTACAATCTGCTCTGATGCCGCATAGTTAAGCCAGCCCCGACACCCGCCAACACCCGCTGACGCGCCCTGACGGGCTTGTCTGCTCCCGGCATCCGCTTACAGACAAGCTGTGACCGTCTCCGGGAGCTGCATGTGTCAGAGGTTTTCACCGTCATCACCGAAACGCGCGAGACGAAAGGGCCTCGTGATACGCCTATTTTTATAGGTTAATGTCATGATAATAATGGTTTCTTAGACGTCAGGTGGCACTTTTCGGGGAAATGTGCGCGGAACCCCTATTTGTTTATTTTTCTAAATACATTCAAATATGTATCCGCTCATGAGACAATAACCCTGATAAATGCTTCAATAATATTGAAAAAGGAAGAGTATGAGTATTCAACATTTCCGTGTCGCCCTTATTCCCTTTTTTGCGGCATTTTGCCTTCCTGTTTTTGCTCACCCAGAAACGCTGGTGAAAGTAAAAGATGCTGAAGATCAGTTGGGTGCACGAGTGGGTTACATCGAACTGGATCTCAACAGCGGTAAGATCCTTGAGAGTTTTCGCCCCGAAGAACGTTTTCCAATGATGAGCACTTTTAAAGTTCTGCTATGTGGCGCGGTATTATCCCGTATTGACGCCCGGGCAAGAGCAACTCGGTCGCCGCATACACTATTCCTCAAGAATGACTTGGTTTGAGTACTCACCAGTCACAGAAAAGGCATCTTACGGATGGCATGACAGTTAAGAGAATTATGCATTGCTTGCCATAACATGGAGTGAATAAACACTGGCCGCACTTACCTTCTTGACACGAATTCGGGAGGAACGATGGAGCCTAACGGCTTTTTGCCAACATGGGGGAATCTGGTAACTCGCCCTGAATCGTGGACCCGGGAGCCTGAATGGTGAAGCCCTGCT

NOVEL57 5-GCAGTCTCCTTGGCTAAGC-3

RT-primer：5-GTCGTATCCAGTGCAGGGTCCGAGGTATTCGCACTGGATACGACGCTTAG-3

Forward primer： 5-CGGCGGCAGTCTCCTTGG-3

Amplicon was verified by sequencing

AGACAATGACATGATTACGAATTCGAGCTCGGTACCCGGGGATCCTCTAGAGATTCGGCGGCAGTCTCCTTGGCTAAGCGTCGTATCCAGTGCGAATACCTCGGACCCTGCACAATCGTCGACCTGCAGGCATGCAAGCTTGGCACTGGCCGTCGTTTTACAACGTCGTGACTGGGAAAACCCTGGCGTTACCCAACTTAATCGCCTTGCAGCACATCCCCCTTTCGCCAGCTGGCGTAATAGCGAAGAGGCCCGCACCGATCGCCCTTCCCAACAGTTGCGCAGCCTGAATGGCGAATGGCGCCTGATGCGGTATTTTCTCCTTACGCATCTGTGCGGTATTTCACACCGCATATGGTGCACTCTCAGTACAATCTGCTCTGATGCCGCATAGTTAAGCCAGCCCCGACACCCGCCAACACCCGCTGACGCGCCCTGACGGGCTTGTCTGCTCCCGGCATCCGCTTACAGACAAGCTGTGACCGTCTCCGGGAGCTGCATGTGTCAGAGGTTTTCACCGTCATCACCGAAACGCGCGAGACGAAAGGGCCTCGTGATACGCCTATTTTTATAGGTTAATGTCATGATAATAATGGTTTCTTAGACGTCAGGTGGCACTTTTCGGGGAAATGTGCGCGGAACCCCTATTTGTTTATTTTTCTAAATACATTCAAATATGTATCCGCTCATGAGACAATAACCCTGATAAATGCTTCAATAATATTGAAAAAGGAAGAGTATGAGTATTCAACATTTCCGTGTCGCCCTTATTCCCTTTTTTGCGGCATTTTGCCTTCCTGTTTTTGCTCACCCAGAAACGCTAGTGAAAGTAAAAGATGCTGAAGATCAGTTGGGTGCACGAGTGGGTTACATCGAACTGGATCTCAACAGCGGTAGATCCTTGAGAGTTTTCGCCCCGAAGAACGTTTTCCAATGATGAGCACTTTTAAAGTTCTGCTATGTGGCGCGTATTATCCCGTATGACGCCAGGCAGAGCACTCGGTCGCGCATACACTATTCTCAGAATGACTTGTTGGAGTACTCACCAGTCACAGAAAAGCATCTACGATGCATGACGGTAGAGATTATGCATGCTGCATACATGATGATAAACTGCGGCACTTACTCTGAAACGATGAGGACCAAGAGCTACCGCATTTTGCCACAAGGGACTGTATCGCTGACCTTGGACGACGTATGAGCTCATGACAACGGTAATCG

NOVEL58 5-TAGCCAAGGATGACTTGCCTGA-3

RT-primer：5-GTCGTATCCAGTGCAGGGTCCGAGGTATTCGCACTGGATACGACTCAGGC-3

Forward primer： 5-CAGCGTAGCCAAGGATGACTT-3

Amplicon was verified by sequencing

ACGACATGACATGATTACGATTCGAGCTCGGTACCCGGGGATCCTCTAGAGATTCAGCGTAGCCAAGGATGACTTGCCTGAGTCGTATCCAGTGCGAATACCTCGGACCCTGCACAATCGTCGACCTGCAGGCATGCAAGCTTGGCACTGGCCGTCGTTTTACAACGTCGTGACTGGGAAAACCCTGGCGTTACCCAACTTAATCGCCTTGCAGCACATCCCCCTTTCGCCAGCTGGCGTAATAGCGAAGAGGCCCGCACCGATCGCCCTTCCCAACAGTTGCGCAGCCTGAATGGCGAATGGCGCCTGATGCGGTATTTTCTCCTTACGCATCTGTGCGGTATTTCACACCGCATATGGTGCACTCTCAGTACAATCTGCTCTGATGCCGCATAGTTAAGCCAGCCCCGACACCCGCCAACACCCGCTGACGCGCCCTGACGGGCTTGTCTGCTCCCGGCATCCGCTTACAGACAAGCTGTGACCGTCTCCGGGAGCTGCATGTGTCAGAGGTTTTCACCGTCATCACCGAAACGCGCGAGACGAAAGGGCCTCGTGATACGCCTATTTTTATAGGTTAATGTCATGATAATAATGGTTTCTTAGACGTCAGGTGGCACTTTTCGGGGAAATGTGCGCGGAACCCCTATTTGTTTATTTTTCTAAATACATTCAAATATGTATCCGCTCATGAGACAATAACCCTGATAAATGCTTCAATAATATTGAAAAAGGAAGAGTATGAGTATTCAACATTTCCGTGTCGCCCTTATTCCCTTTTTTGCGGCATTTTGCCTTCCTGTTTTTGCTCACCCAGAAACGCTGGTGAAAGTAAAAGATGCTGAAGATCAGTTGGGTGCACGAGTGGGTTACATCGAACTGGATCTCAACAGCGGTAAGATCCTTGAGAGTTTTCGCCCCGAAGAACGTTTTCCAATGATGAGCACTTTTAAAGTTCTGCTATGTGGCGCGGTATTATCCCGTATTGACGCCGGGCAAGAGCAACTCGGTCGCCGCATACACTATTCTCAGATGACTTGGTTGAGTACTCACCAGTCACAGAAAAGCATCTTACGATGGCATGACAGTAGAGATTATGCAGTGCTGCATACCATGGAGTGATAAACACTGGCGCACTACTTCTGACAACGATCGAGACCGAGGAGCTACCGCCTTTTTGCACAACATTGGATCAGGTACCTGCTCTTGTATCGTGTGGGAACCGGGACCTTGTATATATATGAAGAGAAGAACCTCATCATCTCTCCA

NOVEL59 5-TTGGACCAGGCTTCATTCCTC-3

RT-primer：5-GTCGTATCCAGTGCAGGGTCCGAGGTATTCGCACTGGATACGACGAGGAA-3

Forward primer： 5-CGGCGTTGGACCAGGCTTCA-3

Amplicon was verified by sequencing

AATTAACCTGAATTACCGATTCGAGCTCGGTACCCGGGGATCCTCTAGAGATTCGGCGTTGGACCAGGCTTCATTCCTCGTCGTATCCAGTGCGAATACCTCGGACCCTGCACAATCGTCGACCTGCAGGCATGCAAGCTTGGCACTGGCCGTCGTTTTACAACGTCGTGACTGGGAAAACCCTGGCGTTACCCAACTTAATCGCCTTGCAGCACATCCCCCTTTCGCCAGCTGGCGTAATAGCGAAGAGGCCCGCACCGATCGCCCTTCCCAACAGTTGCGCAGCCTGAATGGCGAATGGCGCCTGATGCGGTATTTTCTCCTTACGCATCTGTGCGGTATTTCACACCGCATATGGTGCACTCTCAGTACAATCTGCTCTGATGCCGCATAGTTAAGCCAGCCCCGACACCCGCCAACACCCGCTGACGCGCCCTGACGGGCTTGTCTGCTCCCGGCATCCGCTTACAGACAAGCTGTGACCGTCTCCGGGAGCTGCATGTGTCAGAGGTTTTCACCGTCATCACCGAAACGCGCGAGACGAAAGGGCCTCGTGATACGCCTATTTTTATAGGTTAATGTCATGATAATAATGGTTTCTTAGACGTCAGGTGGCACTTTTCGGGGAAATGTGCGCGGAACCCCTATTTGTTTATTTTTCTAAATACATTCAAATATGTATCCGCTCATGAGACAATAACCCTGATAAATGCTTCAATAATATTGAAAAAGGAAGAGTATGAGTATTCAACATTTCCGTGTCGCCCTTATTCCCTTTTTTGCGGCATTTTGCCTTCCTGTTTTTGCTCACCCAGAAACGCTGGTGAAAGTAAAAGATGCTGAAGATCAGTTGGGTGCACGAGTGGGTTACATCGAACTGGATCTCAACAGCGGTAAGATCCTTGAGAGTTTTCGCCCCGAAGAACGTTTTCCAATGATGAGCACTTTTAAAGTTCTGCTATGTGGCGCGGTATTATCCCGTATTGACGCCCGGGCAAGAGCAACTCGGTCGCCGCATACACTATTCCTCAAGAATGACTTGGTTTGAGTACTCACCAGTCACAGAAAAGGCATCTTACGGATGGCATGACAGTTAAGAGAATTATGCATTGCTTGCCATAACATGGAGTGAATAAACACTGGCCGCACTTACCTTCTTGACACGAATTCGGGAGGAACGATGGAGCCTAACGGCTTTTTGCCAACATGGGGGAATCTGGTAACTCGCCCTGAATCGTGGACCCGGGAGCCTGAATGGTGAAGCCCTGCT

NOVEL60 5-TCAAGTTAGAGAGAGAAAGCGT-3

RT-primer：5-GTCGTATCCAGTGCAGGGTCCGAGGTATTCGCACTGGATACGACACGCTT-3

Forward primer： 5-CAGCGTCAAGTTAGAGAGAGA-3

Amplicon was verified by sequencing

TAGAATATAGGCTTCACTAATACAGGTCCTGTTCCAAACGATTACAAGGCGATTACCTGATCCCCATGTGGGCAAAAAAGCGGTAGGCTCCGTGCGGTCTCCGATTCGTTTGTCAGAAAGTAAGTCGCCGCCAGTGTTTTTCACTCCATGGTTATGGCAGCATGCATAATTTCTTCTTACTGTCATGCCCATCGTAAGATGCTTTTTCTGTGACTGGTGGAGTACTCAACCAAGTCATCTGAGAATAGTGTATGCGGGCGACCGAGTTGCTCTGCCGGCGTCAATACGGGATAATACCGCGCCACATAGCAGAACTTTAAAAGTGCTCATCATTGGAAAACGTTCTTCGGGGCGAAAACTCTCAAGGATCTACCGCTGTTGAGATCCAGTTCGATGTAACCCACTCGTGCACCCAACTGATCTTCAGCATCTTTTACTTTCACCAGCGTTTCTGGGTGAGCAAAAACAGGAAGGCAAAATGCCGCAAAAAAGGGAATAAGGGCGACACGGAAATGTTGAATACTCATACTCTTCCTTTTTCAATATTATTGAAGCATTTATCAGGGTTATTGTCTCATGAGCGGATACATATTTGAATGTATTTAGAAAAATAAACAAATAGGGGTTCCGCGCACATTTCCCCGAAAAGTGCCACCTGACGTCTAAGAAACCATTATTATCATGACATTAACCTATAAAAATAGGCGTATCACGAGGCCCTTTCGTCTCGCGCGTTTCGGTGATGACGGTGAAAACCTCTGACACATGCAGCTCCCGGAGACGGTCACAGCTTGTCTGTAAGCGGATGCCGGGAGCAGACAAGCCCGTCAGGGCGCGTCAGCGGGTGTTGGCGGGTGTCGGGGCTGGCTTAACTATGCGGCATCAGAGCAGATTGTACTGAGAGTGCACCATATGCGGTGTGAAATACCGCACAGATGCGTAAGGAGAAAATACCGCATCAGGCGCCATTCGCCATTCAGGCTGCGCAACTGTTGGGAAGGGCGATCGGTGCGGGCCTCTTCGCTATTACGCCAGCTGGCGAAAGGGGGATGTGCTGCAAGGCGATTAAGTTGGGTAACGCCAGGGTTTTCCCAGTCACGACGTTGTAAAACGACGGCCAGTGCCAAGCTTGCATGCCTGCAGGTCGACGATTCAGCGTCAAGTTAGAGAGAGAAAGCGTGTCGTATCCAGTGCGAATACCTCGGACCCTGCACAATCTCTAGAGGATCCCCGGGTACCGAGCTCGAATCGTAATCATGTCATGTCT

NOVEL61 5-TTGGACTGAAGGGAGCTCCC-3

RT-primer：5-GTCGTATCCAGTGCAGGGTCCGAGGTATTCGCACTGGATACGACGGGAGC-3

Forward primer： 5-CAGCGTTGGACTGAAGGGA-3

Amplicon was verified by sequencing

GGGACTTCATTTACAGGCTCCCGAGTTCCAACCGAATAAGGCGAGTACATGAATTCCCCCATGTTGGTGCAAAAAAGCGTAGTCCTTCGTCCTCCGATCGTTTTCAGAGTAGGTGGCCGCCAGTGTTATCACTCCATGTATGCAGCACTGCATAATCTCTACTGTCATGCCATCGTAAGATGCTTTTCTGTGACTGGTGAGTACTCAACCAAGTCATCTGAGAATAGTGTATGCGGCGACCGAGTTGCTCTTGCCCGGCGTCAATACGGGATAATACCGCGCCACATAGCAGAACTTTAAAAGTGCTCATCATTGGAAAACGTTCTTCGGGGCGAAAACTCTCAAGGATCTTACCGCTGTTGAGATCCAGTTCGATGTAACCCACTCGTGCACCCAACTGATCTTCAGCATCTTTTACTTTCACCAGCGTTTCTGGGTGAGCAAAAACAGGAAGGCAAAATGCCGCAAAAAAGGGAATAAGGGCGACACGGAAATGTTGAATACTCATACTCTTCCTTTTTCAATATTATTGAAGCATTTATCAGGGTTATTGTCTCATGAGCGGATACATATTTGAATGTATTTAGAAAAATAAACAAATAGGGGTTCCGCGCACATTTCCCCGAAAAGTGCCACCTGACGTCTAAGAAACCATTATTATCATGACATTAACCTATAAAAATAGGCGTATCACGAGGCCCTTTCGTCTCGCGCGTTTCGGTGATGACGGTGAAAACCTCTGACACATGCAGCTCCCGGAGACGGTCACAGCTTGTCTGTAAGCGGATGCCGGGAGCAGACAAGCCCGTCAGGGCGCGTCAGCGGGTGTTGGCGGGTGTCGGGGCTGGCTTAACTATGCGGCATCAGAGCAGATTGTACTGAGAGTGCACCATATGCGGTGTGAAATACCGCACAGATGCGTAAGGAGAAAATACCGCATCAGGCGCCATTCGCCATTCAGGCTGCGCAACTGTTGGGAAGGGCGATCGGTGCGGGCCTCTTCGCTATTACGCCAGCTGGCGAAAGGGGGATGTGCTGCAAGGCGATTAAGTTGGGTAACGCCAGGGTTTTCCCAGTCACGACGTTGTAAAACGACGGCCAGTGCCAAGCTTGCATGCCTGCAGGTCGACGATTCAGCGTTGGACTGAAGGGAGCTCCCGTCGTATCCAGTGCGAATACCTCGGACCCTGCACAATCTCTAGAGGATCCCCGGGTACCGAGCTCGAATTCGTAATCATGTCATTGTCCT

NOVEL62 5-CTACTTGGTAGGATACTTGGG-3

RT-primer：5-GTCGTATCCAGTGCAGGGTCCGAGGTATTCGCACTGGATACGACCCCAAG-3

Forward primer： 5-CATAGCTACTTGGTAGGATA-3

Amplicon was verified by sequencing

CCGACAATGACATGCATTACGATTCGAGCTCGGTACCCGGGGATCCTCTAGAGATTCATAGCTACTTGGTAGGATACTTGGGGTCGTATCCAGTGCGAATACCTCGGACCCTGCACAATCGTCGACCTGCAGGCATGCAAGCTTGGCACTGGCCGTCGTTTTACAACGTCGTGACTGGGAAAACCCTGGCGTTACCCAACTTAATCGCCTTGCAGCACATCCCCCTTTCGCCAGCTGGCGTAATAGCGAAGAGGCCCGCACCGATCGCCCTTCCCAACAGTTGCGCAGCCTGAATGGCGAATGGCGCCTGATGCGGTATTTTCTCCTTACGCATCTGTGCGGTATTTCACACCGCATATGGTGCACTCTCAGTACAATCTGCTCTGATGCCGCATAGTTAAGCCAGCCCCGACACCCGCCAACACCCGCTGACGCGCCCTGACGGGCTTGTCTGCTCCCGGCATCCGCTTACAGACAAGCTGTGACCGTCTCCGGGAGCTGCATGTGTCAGAGGTTTTCACCGTCATCACCGAAACGCGCGAGACGAAAGGGCCTCGTGATACGCCTATTTTTATAGGTTAATGTCATGATAATAATGGTTTCTTAGACGTCAGGTGGCACTTTTCGGGGAAATGTGCGCGGAACCCCTATTTGTTTATTTTTCTAAATACATTCAAATATGTATCCGCTCATGAGACAATAACCCTGATAAATGCTTCAATAATATTGAAAAAGGAAGAGTATGAGTATTCAACATTTCCGTGTCGCCCTTATTCCCTTTTTTGCGGCATTTTGCCTTCCTGTTTTTGCTCACCCAGAAACGCTGGTGAAAGTAAAAGATGCTGAAGATCAGTTGGGTGCACGAGTGGGTTACATCGAACTGGATCTCAACAGCGGTAAGATCCTTGAGAGTTTTCGCCCCGAAGAACGTTTTCCAATGATGAGCACTTTTAAAGTTCTGCTATGTGGCGCGGTATTATCCCGTATTGACGCCGGGCAAGAGCAACTCGGTCGCCGCATACACTATTCTCAGAATGACTTGGTTGAGTACTCCCCAGTCACAGAAAAGCATCTTACGGATGGCATGACAGTAGAGAATTATGCAGTGCTGCCATACCATGGAGTGATACACTGGCGGGCCACTACTCTGACACGATCGGAGGACGGAAGGAGCTACGCTTTTTGCACAACATGGGGGATCATGTAACTCCCTGGAATCGGTGACGGATCTGGAACGTGGAATGTGTGAGCCATATCCAATCGCACCACAGACGT

NOVEL63 5-TACAACTGTGGCAAGAAATGGCA-3

RT-primer：5-GTCGTATCCAGTGCAGGGTCCGAGGTATTCGCACTGGATACGACTGCCAT-3

Forward primer： 5-CAGCCTACAACTGTGGCAAGAA-3

Amplicon was verified by sequencing

CAAAATTTGACCTTGTATTACGAATTTCGAGCTCGGTACCCGGGGATCCTCTAGAGATTCAGCCTACAACTGTGGCAAGAAATGGCAGTCGTATCCAGTGCGAATACCTCGGACCCTGCACAATCGTCGACCTGCAGGCATGCAAGCTTGGCACTGGCCGTCGTTTTACAACGTCGTGACTGGGAAAACCCTGGCGTTACCCAACTTAATCGCCTTGCAGCACATCCCCCTTTCGCCAGCTGGCGTAATAGCGAAGAGGCCCGCACCGATCGCCCTTCCCAACAGTTGCGCAGCCTGAATGGCGAATGGCGCCTGATGCGGTATTTTCTCCTTACGCATCTGTGCGGTATTTCACACCGCATATGGTGCACTCTCAGTACAATCTGCTCTGATGCCGCATAGTTAAGCCAGCCCCGACACCCGCCAACACCCGCTGACGCGCCCTGACGGGCTTGTCTGCTCCCGGCATCCGCTTACAGACAAGCTGTGACCGTCTCCGGGAGCTGCATGTGTCAGAGGTTTTCACCGTCATCACCGAAACGCGCGAGACGAAAGGGCCTCGTGATACGCCTATTTTTATAGGTTAATGTCATGATAATAATGGTTTCTTAGACGTCAGGTGGCACTTTTCGGGGAAATGTGCGCGGAACCCCTATTTGTTTATTTTTCTAAATACATTCAAATATGTATCCGCTCATGAGACAATAACCCTGATAAATGCTTCAATAATATTGAAAAAGGAAGAGTATGAGTATTCAACATTTCCGTGTCGCCCTTATTCCCTTTTTTGCGGCATTTTGCCTTCCTGTTTTTGCTCACCCAGAAACGCTGGTGAAAGTAAAAGATGCTGAAGATCAGTTGGGTGCACGAGTGGGTTACATCGAACTGGATCTCAACAGCGGTAAGATCCTTTGAGAGTTTTCGCCCCGGAGAACGTTTTTCCATGATGAGCACTTTTAAAGGTTCTTGCTATGTGGCGCGGTATTATCCCGTAATTGACGCCGGACAAGAGCAACTCGGTTCCGCCGCATACACCTTATTCCTCAGATGACTTGGTTGAGTACTCCACCATGTCACAGAAAAAGCATCCTTACGATGGCATGACGTAAGAAGAATTATTGCCATGCTGCAATAACCATGAGTGATTAACACTGGCGACCACCTTACTTCTGACCACGATATCGGAGGAACCGAAGGACTTAACGCTTTTGGCACCACTTGGGAATCCGTTACTGCCCTGTATTCGTTGAACCGGAACCGTGAATGTAGCCTAATCATAGCGAT

NOVEL64 5-CAATGAGAGCTGGCCATGTGGG-3

RT-primer：5-GTCGTATCCAGTGCAGGGTCCGAGGTATTCGCACTGGATACGACCCCACA-3

Forward primer： 5-CGGCGCAATGAGAGCTGGCCA-3

Amplicon was verified by sequencing

TCGGACAAGACATGATTACGATTCGAGCTCGGTACCCGGGGATCCTCTAGAGATTCGGCGCAATGAGAGCTGGCCATGTGGGGTCGTATCCAGTGCGAATACCTCGGACCCTGCACAATCGTCGACCTGCAGGCATGCAAGCTTGGCACTGGCCGTCGTTTTACAACGTCGTGACTGGGAAAACCCTGGCGTTACCCAACTTAATCGCCTTGCAGCACATCCCCCTTTCGCCAGCTGGCGTAATAGCGAAGAGGCCCGCACCGATCGCCCTTCCCAACAGTTGCGCAGCCTGAATGGCGAATGGCGCCTGATGCGGTATTTTCTCCTTACGCATCTGTGCGGTATTTCACACCGCATATGGTGCACTCTCAGTACAATCTGCTCTGATGCCGCATAGTTAAGCCAGCCCCGACACCCGCCAACACCCGCTGACGCGCCCTGACGGGCTTGTCTGCTCCCGGCATCCGCTTACAGACAAGCTGTGACCGTCTCCGGGAGCTGCATGTGTCAGAGGTTTTCACCGTCATCACCGAAACGCGCGAGACGAAAGGGCCTCGTGATACGCCTATTTTTATAGGTTAATGTCATGATAATAATGGTTTCTTAGACGTCAGGTGGCACTTTTCGGGGAAATGTGCGCGGAACCCCTATTTGTTTATTTTTCTAAATACATTCAAATATGTATCCGCTCATGAGACAATAACCCTGATAAATGCTTCAATAATATTGAAAAAGGAAGAGTATGAGTATTCAACATTTCCGTGTCGCCCTTATTCCCTTTTTTGCGGCATTTTGCCTTCCTGTTTTTGCTCACCCAGAAACGCTGGTGAAAGTAAAAGATGCTGAAGATCAGTTGGGTGCACGAGTGGGTTACATCGAACTGGATCTCAACAGCGGTAAGATCCTTGAGAGTTTTCGCCCCGAAGAACGTTTTCCAATGATGAGCACTTTTAAAGTTCTGCTATGTGGCGCGGTATTATCCCGTATTGACGCCGGGCAAGAGCAACTCGGTCGCCGCATACACTATTCCTCAGATTGACTTGGGTTGAGTACTCACCAGTCACAGAAAAGCATCCTTACGGATGGCATGACAGTAGAGAATTATGCAGTGCTGCCATACCATGATTGATACACTGCCGCCACCTACCTTCTGACACCGATCGGAGAACGAGGAGCCTACCCGCTTTTTTGCCCAACATGGGGGATCTAGTATCTCGTCCTTGAATGCGGTGGGATCCGGATACCT

NOVEL65 5-CGAGCAGATCCTGACACGCTT-3

RT-primer：5-GTCGTATCCAGTGCAGGGTCCGAGGTATTCGCACTGGATACGACAAGCGT-3

Forward primer： 5-CAGCGCGAGCAGATCCTGAC-3

NOVEL66 5-AAATTACGTTCTCGTTGGGTA-3

RT-primer：5-GTCGTATCCAGTGCAGGGTCCGAGGTATTCGCACTGGATACGACTACCCA-3

Forward primer： 5-CAGCGAAATTACGTTCTCGT-3

Amplicon was verified by sequencing

TCGGACTTTGGACATGATTACGATTCGAGCTCGGTACCCGGGGATCCTCTAGAGATTCAGCGAAATTACGTTCTCGTTGGGTAGTCGTATCCAGTGCGAATACCTCGGACCCTGCACAATCGTCGACCTGCAGGCATGCAAGCTTGGCACTGGCCGTCGTTTTACAACGTCGTGACTGGGAAAACCCTGGCGTTACCCAACTTAATCGCCTTGCAGCACATCCCCCTTTCGCCAGCTGGCGTAATAGCGAAGAGGCCCGCACCGATCGCCCTTCCCAACAGTTGCGCAGCCTGAATGGCGAATGGCGCCTGATGCGGTATTTTCTCCTTACGCATCTGTGCGGTATTTCACACCGCATATGGTGCACTCTCAGTACAATCTGCTCTGATGCCGCATAGTTAAGCCAGCCCCGACACCCGCCAACACCCGCTGACGCGCCCTGACGGGCTTGTCTGCTCCCGGCATCCGCTTACAGACAAGCTGTGACCGTCTCCGGGAGCTGCATGTGTCAGAGGTTTTCACCGTCATCACCGAAACGCGCGAGACGAAAGGGCCTCGTGATACGCCTATTTTTATAGGTTAATGTCATGATAATAATGGTTTCTTAGACGTCAGGTGGCACTTTTCGGGGAAATGTGCGCGGAACCCCTATTTGTTTATTTTTCTAAATACATTCAAATATGTATCCGCTCATGAGACAATAACCCTGATAAATGCTTCAATAATATTGAAAAAGGAAGAGTATGAGTATTCAACATTTCCGTGTCGCCCTTATTCCCTTTTTTGCGGCATTTTGCCTTCCTGTTTTTGCTCACCCAGAAACGCTGGTGAAAGTAAAAGATGCTGAAGATCAGTTGGGTGCACGAGTGGGTTACATCGAACTGGATCTCAACAGCGGTAAGATCCTTGAGAGTTTTCGCCCCGAAGAACGTTTTCCAATGATGAGCACTTTTAAAGTTCTGCTATGTGGCGCGGTATTATCCCGTATTGACGCCCGGGCAAGAGCAACTCGGTCGCCGCATACACTATTCTCAGATGACTTGGTTGAGTACTCACCAGTCACAGAAAAGCATCTTACGATGGCATGACAGTAGGAGATTATGCAGTGCTGCATACCATGAGTGATACACTGCGCACTACTCTGACAACGATCGGAGGAACGAAGAAGCTACGGCTTTTTTGGCACACATGGGATCTCAGTAACTCGCCTGGATCGTGGACCGGGAGCTGAATGTGAAGC

NOVEL67 5-AGCAGATGATGATACAAAAACA-3

RT-primer：5-GTCGTATCCAGTGCAGGGTCCGAGGTATTCGCACTGGATACGACTGTTTT-3

Forward primer： 5-CGCCGAGCAGATGATGATACA-3

Amplicon was verified by sequencing

CTGGATTCCCCCCATGTTTGTGCAAAAAGCGGTAGCTCCTCGTCCTCGATCGTGTCAGGAGGTAGTGCGCAGTGTATCATCATGTTATGCAGCACTGCATATTCTCTACTGTCATGCCATCCGTAAGATGCTTTTCTGTGACTGGTGGAGTACTCAACCAAGTCATTCTGAGAATAGTGTATGCGGCGACCGAGTTGCTCTTGCCCGGCGTCAATACGGGATAATACCGCGCCACATAGCAGAACTTTAAAAGTGCTCATCATTGGAAAACGTTCTTCGGGGCGAAAACTCTCAAGGATCTTACCGCTGTTGAGATCCAGTTCGATGTAACCCACTCGTGCACCCAACTGATCTTCAGCATCTTTTACTTTCACCAGCGTTTCTGGGTGAGCAAAAACAGGAAGGCAAAATGCCGCAAAAAAGGGAATAAGGGCGACACGGAAATGTTGAATACTCATACTCTTCCTTTTTCAATATTATTGAAGCATTTATCAGGGTTATTGTCTCATGAGCGGATACATATTTGAATGTATTTAGAAAAATAAACAAATAGGGGTTCCGCGCACATTTCCCCGAAAAGTGCCACCTGACGTCTAAGAAACCATTATTATCATGACATTAACCTATAAAAATAGGCGTATCACGAGGCCCTTTCGTCTCGCGCGTTTCGGTGATGACGGTGAAAACCTCTGACACATGCAGCTCCCGGAGACGGTCACAGCTTGTCTGTAAGCGGATGCCGGGAGCAGACAAGCCCGTCAGGGCGCGTCAGCGGGTGTTGGCGGGTGTCGGGGCTGGCTTAACTATGCGGCATCAGAGCAGATTGTACTGAGAGTGCACCATATGCGGTGTGAAATACCGCACAGATGCGTAAGGAGAAAATACCGCATCAGGCGCCATTCGCCATTCAGGCTGCGCAACTGTTGGGAAGGGCGATCGGTGCGGGCCTCTTCGCTATTACGCCAGCTGGCGAAAGGGGGATGTGCTGCAAGGCGATTAAGTTGGGTAACGCCAGGGTTTTCCCAGTCACGACGTTGTAAAACGACGGCCAGTGCCAAGCTTGCATGCCTGCAGGTCGACGATTCGCCGAGCAGATGATGATACAAAAACAGTCGTATCCAGTGCGAATACCTCGGACCCTGCACTGGATACGACAATCTCTAGAGGATCCCCGGGTACCGAGCTCGAATCGTAATCATGTTAAAATGTTC

NOVEL68 5-TGCTGTGATGATTTATGAATGAAT-3

RT-primer：5-GTCGTATCCAGTGCAGGGTCCGAGGTATTCGCACTGGATACGACATTCAT-3

Forward primer： 5-CGGCGTGCTGTGATGATTTATGA-3

Amplicon was verified by sequencing

TTTACAAGGATTACGATTTCGAGCTCGGTACCCGGGGATCCTCTAGAGATTCGGCGTGCTGTGATGATTTATGAATGAATGTCATATCCAGTGCGAATACCTCGGACCCTGCACAATCGTCGACCTGCAGGCATGCAAGCTTGGCACTGGCCGTCGTTTTACAACGTCGTGACTGGGAAAACCCTGGCGTTACCCAACTTAATCGCCTTGCAGCACATCCCCCTTTCGCCAGCTGGCGTAATAGCGAAGAGGCCCGCACCGATCGCCCTTCCCAACAGTTGCGCAGCCTGAATGGCGAATGGCGCCTGATGCGGTATTTTCTCCTTACGCATCTGTGCGGTATTTCACACCGCATATGGTGCACTCTCAGTACAATCTGCTCTGATGCCGCATAGTTAAGCCAGCCCCGACACCCGCCAACACCCGCTGACGCGCCCTGACGGGCTTGTCTGCTCCCGGCATCCGCTTACAGACAAGCTGTGACCGTCTCCGGGAGCTGCATGTGTCAGAGGTTTTCACCGTCATCACCGAAACGCGCGAGACGAAAGGGCCTCGTGATACGCCTATTTTTATAGGTTAATGTCATGATAATAATGGTTTCTTAGACGTCAGGTGGCACTTTTCGGGGAAATGTGCGCGGAACCCCTATTTGTTTATTTTTCTAAATACATTCAAATATGTATCCGCTCATGAGACAATAACCCTGATAAATGCTTCAATAATATTGAAAAAGGAAGAGTATGAGTATTCAACATTTCCGTGTCGCCCTTATTCCCTTTTTTGCGGCATTTTGCCTTCCTGTTTTTGCTCACCCAGAAACGCTGGTGAAAGTAAAAGATGCTGAAGATCAGTTGGGTGCACGAGTGGGTTACATCGAACTGGATCTCAACAGCGGTAAGATCCTTGAGAGTTTTCGCCCCGAAGACGTTTTCCAATGATGAGCACTTTTAAAGTTCTGCTATGTGGCGCGGTATTATCCCGTATTGACGCCGGGCAAGAGCAACTCGGTTCGCCGCATACACTATTCTCAGAATGACTTGGGTTGAGTACTCACCAGTCACAGAAAGCATCCTTACCGGATGGCATGACAGTAGAGATTATTGCAGTGCTGCCATTACCAATGAAGTGAATAACACTGGCGGCCAACTTTACTTTCTGACAACCGAATCGAGGACGGATGGAGCCTTAACCGCTTTTTTGCACACATGGGGGAATCTTGTTACTCGCCTGATCGATTGGAACCGGAGACTTGATGTAAGCCATATACGTAGACGCAGCAGGCCCGT

NOVEL69 5-TAAAGAGGGTAAAATAGCTAACA-3

RT-primer：5-GTCGTATCCAGTGCAGGGTCCGAGGTATTCGCACTGGATACGACTGTTAG-3

Forward primer： 5-CATCGTAAAGAGGGTAAAATAG-3

Amplicon was verified by sequencing

ATTTGGACATGCATTTACGATTCGAGCTCGGTACCCGGGGATCCTCTAGAGATTCATCGTAAAGAGGGTAAAATAGCTAACAGTCGTATCCAGTGCGAATACCTCGGACCCTGCACAATCGTCGACCTGCAGGCATGCAAGCTTGGCACTGGCCGTCGTTTTACAACGTCGTGACTGGGAAAACCCTGGCGTTACCCAACTTAATCGCCTTGCAGCACATCCCCCTTTCGCCAGCTGGCGTAATAGCGAAGAGGCCCGCACCGATCGCCCTTCCCAACAGTTGCGCAGCCTGAATGGCGAATGGCGCCTGATGCGGTATTTTCTCCTTACGCATCTGTGCGGTATTTCACACCGCATATGGTGCACTCTCAGTACAATCTGCTCTGATGCCGCATAGTTAAGCCAGCCCCGACACCCGCCAACACCCGCTGACGCGCCCTGACGGGCTTGTCTGCTCCCGGCATCCGCTTACAGACAAGCTGTGACCGTCTCCGGGAGCTGCATGTGTCAGAGGTTTTCACCGTCATCACCGAAACGCGCGAGACGAAAGGGCCTCGTGATACGCCTATTTTTATAGGTTAATGTCATGATAATAATGGTTTCTTAGACGTCAGGTGGCACTTTTCGGGGAAATGTGCGCGGAACCCCTATTTGTTTATTTTTCTAAATACATTCAAATATGTATCCGCTCATGAGACAATAACCCTGATAAATGCTTCAATAATATTGAAAAAGGAAGAGTATGAGTATTCAACATTTCCGTGTCGCCCTTATTCTCTTTTTTGCGGCATTTTGCCTTCCTGTTGTTGCTCACCCAGAAACGCTGGTGAAAGTAAAAGATGCTGAAGATCAGTTGGGTGCACGAGTGGGTTACATCGAACTGGATCTCAACAGCGGTAAGATCCTTGAGAGTTTTCGCCCCGAAGAACGTTTTCCAATGATGAGCACTTTTAAAGTTCTGCTATGTGGCGCGGTATTATCCCGTATTGACGCCCGGGCAAGAGCAACTCGGTCGCCGCATACACTATTCTCAGAATGACGTGGTTTGAGTACTCACCAGTCACAGAAAAGCATCTTACTGATGGCATGACAGTAGAGATTATTGCAGTGCTGCATAACATGAGTGATAACACTGGCGGCACCTACTTCTGACACGATCGGAGGACGAAGAGCTACGCTTTTTTTGCACACATGGGGATCATGTAACTCCGCTGAATCGTTGGTAACGGCAGCTGAAATGAAGGCCATTACCCAACCGGACG

NOVEL70 5-TCAACTCGGCGATGCATTAATTA-3

RT-primer：5-GTCGTATCCAGTGCAGGGTCCGAGGTATTCGCACTGGATACGACTAATTA-3

Forward primer： 5-CATCGTCAACTCGGCGATGCAT-3

Amplicon was verified by sequencing

TTTACAAGGATTACGATTTCGAGCTCGGTACCCGGGGATCCTCTAGAGATTCATCGTCAACTCGGCGATGCATTAATTAGTCATATCCAGTGCGAATACCTCGGACCCTGCACAATCGTCGACCTGCAGGCATGCAAGCTTGGCACTGGCCGTCGTTTTACAACGTCGTGACTGGGAAAACCCTGGCGTTACCCAACTTAATCGCCTTGCAGCACATCCCCCTTTCGCCAGCTGGCGTAATAGCGAAGAGGCCCGCACCGATCGCCCTTCCCAACAGTTGCGCAGCCTGAATGGCGAATGGCGCCTGATGCGGTATTTTCTCCTTACGCATCTGTGCGGTATTTCACACCGCATATGGTGCACTCTCAGTACAATCTGCTCTGATGCCGCATAGTTAAGCCAGCCCCGACACCCGCCAACACCCGCTGACGCGCCCTGACGGGCTTGTCTGCTCCCGGCATCCGCTTACAGACAAGCTGTGACCGTCTCCGGGAGCTGCATGTGTCAGAGGTTTTCACCGTCATCACCGAAACGCGCGAGACGAAAGGGCCTCGTGATACGCCTATTTTTATAGGTTAATGTCATGATAATAATGGTTTCTTAGACGTCAGGTGGCACTTTTCGGGGAAATGTGCGCGGAACCCCTATTTGTTTATTTTTCTAAATACATTCAAATATGTATCCGCTCATGAGACAATAACCCTGATAAATGCTTCAATAATATTGAAAAAGGAAGAGTATGAGTATTCAACATTTCCGTGTCGCCCTTATTCCCTTTTTTGCGGCATTTTGCCTTCCTGTTTTTGCTCACCCAGAAACGCTGGTGAAAGTAAAAGATGCTGAAGATCAGTTGGGTGCACGAGTGGGTTACATCGAACTGGATCTCAACAGCGGTAAGATCCTTGAGAGTTTTCGCCCCGAAGACGTTTTCCAATGATGAGCACTTTTAAAGTTCTGCTATGTGGCGCGGTATTATCCCGTATTGACGCCGGGCAAGAGCAACTCGGTTCGCCGCATACACTATTCTCAGAATGACTTGGGTTGAGTACTCACCAGTCACAGAAAGCATCCTTACCGGATGGCATGACAGTAGAGATTATTGCAGTGCTGCCATTACCAATGAAGTGAATAACACTGGCGGCCAACTTTACTTTCTGACAACCGAATCGAGGACGGATGGAGCCTTAACCGCTTTTTTGCACACATGGGGGAATCTTGTTACTCGCCTGATCGATTGGAACCGGAGACTTGATGTAAGCCATATACGTAGACGCAGCAGGCCCGT

NOVEL71 5-TGCCTGGCTCCCTGTATGCTT-3

RT-primer：5-GTCGTATCCAGTGCAGGGTCCGAGGTATTCGCACTGGATACGACAAGCAT-3

Forward primer： 5-CAGCGTGCCTGGCTCCCTGT-3

Amplicon was verified by sequencing

TTGGTATTGGCTTCCTTCAGTCCGGTTCCCACGATTCAAGGCGAGTACATGATCCCCCATGTTTGTGCAAAAAGCGTAGCTCTCGGTCTCGATCGTGTCAGAGTAGTTGGCCGCCAGTGTTATCACTCCATGGTATGGCAGCACTGCATAATTCTCTTAACTGTCATGCCATCCCGTAAGATGCTTTTCTGTTGACTGGTGAGTACTCAACCAAGTCATTCTGAGAATAGTGTATGCGGCGACCGAGTTGCTCTTGCCCCGGCGTCAATACGGGATAATACCGCGCCACATAGCAGAACTTTAAAAGTGCTCATCATTGGAAAACGTTCTTCGGGGCGAAAACTCTCAAGGATCTTACCGCTGTTGAGATCCAGTTCGATGTAACCCCACTCGTGCACCCAACTGATCTTCAGCATCTTTTACTTTCACCAGCGTTTCTGGGTGAGCAAAAACAGGAAGGCAAAATGCCGCAAAAAAGGGAATAAGGGCGACACGGAAATGTTGAATACTCATACTCTTCCTTTTTCAATATTATTGAAGCATTTATCAGGGTTATTGTCTCATGAGCGGATACATATTTGAATGTATTTAGAAAAATAAACAAATAGGGGTTCCGCGCACATTTCCCCGAAAAGTGCCACCTGACGTCTAAGAAACCATTATTATCATGACATTAACCTATAAAAATAGGCGTATCACGAGGCCCTTTCGTCTCGCGCGTTTCGGTGATGACGGTGAAAACCTCTGACACATGCAGCTCCCGGAGACGGTCACAGCTTGTCTGTAAGCGGATGCCGGGAGCAGACAAGCCCGTCAGGGCGCGTCAGCGGGTGTTGGCGGGTGTCGGGGCTGGCTTAACTATGCGGCATCAGAGCAGATTGTACTGAGAGTGCACCATATGCGGTGTGAAATACCGCACAGATGCGTAAGGAGAAAATACCGCATCAGGCGCCATTCGCCATTCAGGCTGCGCAACTGTTGGGAAGGGCGATCGGTGCGGGCCTCTTCGCTATTACGCCAGCTGGCGAAAGGGGGATGTGCTGCAAGGCGATTAAGTTGGGTAACGCCAGGGTTTTCCCAGTCACGACGTTGTAAAACGACGGCCAGTGCCAAGCTTGCATGCCTGCAGGTCGACGATTCAGCGTGCCTGGCTCCCTGTATGCTTGTCGTATCCAGTGCGAATACCTCGGACCCTGCACAATCTCTAGAGGATCCCCGGGTACCGAGCTCGAATCGTAATCAATGTTCCAAT

NOVEL72 5-TTCTTATTGATTGTGTATGGTA-3

RT-primer：5-GTCGTATCCAGTGCAGGGTCCGAGGTATTCGCACTGGATACGACTACCAT-3

Forward primer： 5-CGGCGTTCTTATTGATTGTGT-3

Amplicon was verified by sequencing

GAACTTTGAACATGATTACGATTCGAGCTCGGTACCCGGGGATCCTCTAGAGATTCGGCGTTCTTATTGATTGTGTATGGTAGTCGTATCCAGTGCGAATACCTCGGACCCTGCACAATCGTCGACCTGCAGGCATGCAAGCTTGGCACTGGCCGTCGTTTTACAACGTCGTGACTGGGAAAACCCTGGCGTTACCCAACTTAATCGCCTTGCAGCACATCCCCCTTTCGCCAGCTGGCGTAATAGCGAAGAGGCCCGCACCGATCGCCCTTCCCAACAGTTGCGCAGCCTGAATGGCGAATGGCGCCTGATGCGGTATTTTCTCCTTACGCATCTGTGCGGTATTTCACACCGCATATGGTGCACTCTCAGTACAATCTGCTCTGATGCCGCATAGTTAAGCCAGCCCCGACACCCGCCAACACCCGCTGACGCGCCCTGACGGGCTTGTCTGCTCCCGGCATCCGCTTACAGACAAGCTGTGACCGTCTCCGGGAGCTGCATGTGTCAGAGGTTTTCACCGTCATCACCGAAACGCGCGAGACGAAAGGGCCTCGTGATACGCCTATTTTTATAGGTTAATGTCATGATAATAATGGTTTCTTAGACGTCAGGTGGCACTTTTCGGGGAAATGTGCGCGGAACCCCTATTTGTTTATTTTTCTAAATACATTCAAATATGTATCCGCTCATGAGACAATAACCCTGATAAATGCTTCAATAATATTGAAAAAGGAAGAGTATGAGTATTCAACATTTCCGTGTCGCCCTTATTCCCTTTTTTGCGGCATTTTGCCTTCCTGTTTTTGCTCACCCAGAAACGCTGGTGAAAGTAAAAGATGCTGAAGATCAGTTGGGTGCACGAGTGGGTTACATCGAACTGGATCTCAACAGCGGTAAGATCCTTGAGAGTTTTCGCCCCGAAGAACGTTTTCCAATGATGAGCACTTTTAAAGTTCTGCTATGTGGCGCGGTATTATCCCGTATTGACGCCGGGCAAGAGCAACTCGGTCGCCGCATACACTATCTCAGATTGACTGCTGAGTACTCACCAGTCACAGACAGCATCTACGATGCATGACAGTAGAGATATGCATGCTGCATACATGATGAATAACACTGCCGCACCTACTTCGACACGATCGAGACGAAGAACCTAACCGCATTTGCAAACTGGATCCGTTACTCGCCCTTGATCGTTGGATCGGACCTA

NOVEL73 5-AATTCGATTGGTAGATGGGTA-3

RT-primer：5-GTCGTATCCAGTGCAGGGTCCGAGGTATTCGCACTGGATACGACTACCCA-3

Forward primer： 5-CAGCGAATTCGATTGGTAGA-3

Amplicon was verified by sequencing

TAATTTGAAAGTGAAATACGAATTCGAGCTCGGTACCCGGGGATCCTCTAGAGATTCAGCGAATTCGATTGGTAGATGGGTAGTCGTATCCAGTGCGAATACCTCGGACCCTGCACAATCGTCGACCTGCAGGCATGCAAGCTTGGCACTGGCCGTCGTTTTACAACGTCGTGACTGGGAAAACCCTGGCGTTACCCAACTTAATCGCCTTGCAGCACATCCCCCTTTCGCCAGCTGGCGTAATAGCGAAGAGGCCCGCACCGATCGCCCTTCCCAACAGTTGCGCAGCCTGAATGGCGAATGGCGCCTGATGCGGTATTTTCTCCTTACGCATCTGTGCGGTATTTCACACCGCATATGGTGCACTCTCAGTACAATCTGCTCTGATGCCGCATAGTTAAGCCAGCCCCGACACCCGCCAACACCCGCTGACGCGCCCTGACGGGCTTGTCTGCTCCCGGCATCCGCTTACAGACAAGCTGTGACCGTCTCCGGGAGCTGCATGTGTCAGAGGTTTTCACCGTCATCACCGAAACGCGCGAGACGAAAGGGCCTCGTGATACGCCTATTTTTATAGGTTAATGTCATGATAATAATGGTTTCTTAGACGTCAGGTGGCACTTTTCGGGGAAATGTGCGCGGAACCCCTATTTGTTTATTTTTCTAAATACATTCAAATATGTATCCGCTCATGAGACAATAACCCTGATAAATGCTTCAATAATATTGAAAAAGGAAGAGTATGAGTATTCAACATTTCCGTGTCGCCCTTATTCCCTTTTTTGCGGCATTTTGCCTTCCTGTTTTTGCTCACCCAGAAACGCTGGTGAAAGTAAAGATGCTGAAGATCAGTTGGGTGCACGAGTGGGTTACATCGAACTGGATCTCAACAGCGGTAAGATCCTTGAGAGTTTTCGCCCCGAAGAACGTTTTCCAATGATGAGCACTTTTAAAGTTCTGCTATGTGGCGCGGTATTATCCCGTATTGACGCCGGGCAAGAGCAACTCGGTCGCCGCATACACTATTCTCAGAATGACTTGGTTGAGTACCCCACCAGTCCACAGAAAAGCATTCTTACGGATGGCATGACAGTAGAGATTATGCAGTGCTGCATACCATGATTGAATAACACCTGCGCAACTACTTCTGACACGATCGAGACCGAGGACTAACGGCATTTTGGCCCAACATGGATCTGTACTCGCTTGATCGTGAACGGAAGCTGAATTGAAGCCATATCCCAAAC

NOVEL74 5-ATTTCGATCGTCATTGCCAA-3

RT-primer：5-GTCGTATCCAGTGCAGGGTCCGAGGTATTCGCACTGGATACGACTTGGCA-3

Forward primer： 5- GGCGGATTTCGATCGTCAT -3

Amplicon was verified by sequencing

GGACATTGACTGATTACGATTCGAGCTCGGTACCCGGGGATCCTCTAGAGATTCGGCGGTTTCGATCGTCATTGCCAAGTCGTATCCAGTGCGAATACCTCGGACCCTGCACAATCGTCGACCTGCAGGCATGCAAGCTTGGCACTGGCCGTCGTTTTACAACGTCGTGACTGGGAAAACCCTGGCGTTACCCAACTTAATCGCCTTGCAGCACATCCCCCTTTCGCCAGCTGGCGTAATAGCGAATAGGCCCGCACCGATCGCCCTTCCCAACAGTTGCGCAGCCTGAATGGCGAATGGCGCCTGATGCGGTATTTTCTCCTTACGCATCTGTGCGGTATTTCACACCGCATATGGTGCACTCTCAGTACAATCTGCTCTGATGCCGCATAGTTAAGCCAGCCCCGACACCCGCCAACACCCGCTGACGCGCCCTGACGGGCTTGTCTGCTCCCGGCATCCGCTTACAGACAAGCTGTGACCGTCTCCGGGAGCTGCATGTGTCAGAGGTTTTCACCGTCATCACCGAAACGCGCGAGACGAAAGGGCCTCGTGATACGCCTATTTTTATAGGTTAATGTCATGATAATAATGGTTTCTTAGACGTCAGGTGGCACTTTTCGGGGAAATGTGCGCGGAACCCCTATTTGTTTATTTTTCTAAATACATTCAAATATGTATCCGCTCATGAGACAATAACCCTGATAAATGCTTCAATAATATTGAAAAAGGAAGAGTATGAGTATTCAACATTTCCGTGTCGCCCTTATTCCCTTTTTGTGCGGCATTTTGCCTTCCTGTTGTTGCTCACCCAGAAACGCTGGTGAAAGTAAAAGATGCTGAAGATCAGTTGGGTGCACGAGTGGGTTACATCGAACTGGATCTCAACAGCGGTAAGATCCTTGAGAGTTTTCGCCCCGAAGAACGTTTTCCAATGATGAGCACTTTTAAAGTTCTGCTATGTGGCGCGCTATTATCCCGTATTGACGCCGCGCAGAGCAACTCGGTCGCCGCATACACTAATTCTCAGACTGACTTGATGAGTACTCATCAGTCACAGATGAGCATCTACGGATGGCATGACAGTACGAGACTTATGCAGTGCATGCATAGCATGATGATACGCTGCCGACAGCTGACTCTGACCACGATCGGAGGTATCTGAACGAACCTAGCGCATTTTGCACAACATTGGGGAATCTGTTAACTCGACTTGAATCGATGGCATCGGAACTGAATGGAGGTCAATAT

NOVEL75 5-TTGAACGTAATATACACACAC-3

RT-primer：5-GTCGTATCCAGTGCAGGGTCCGAGGTATTCGCACTGGATACGACGTGTGT-3

Forward primer： 5-CATAGTTGAACGTAATATAC-3

Amplicon was verified by sequencing

CGTTCGTATGGCTCATCAGTTCTCGATTCCACGATCAGGGAGGTACATGATCGCCATGTTGGCAAAAAAGCGGTAGCTCCTCGTCCTTCGATCGTGTCAGAGTAGGTGCGCAGTGTATCATCATGTATGCAGCAATGCATAGTTCTCTACTGTCATGCCATCGTAAGATGCTTTTCTGTGACTGGTGAGTACTCAACAAGTCATTCTGAGATAGTGTATGCGGGCGACCGAGTTGCTCTTGCCCGGCGTCAATACGGGATAATAACCGCGCCACATAGCAGAACTTAAAAGTGCTCATCATTGGAAAACGTCTTCGGGGCGAAAACTCTCAAGGATCTTACCGCTGTTGAGATCCAGTTCGATGTAACCCACTCGTGCACCCAACTGATCTTCAGCATCTTTTACTTTCACCAGCGTTTCTGGGTGAGCAAAAACAGGAAGGCAAAATGCCGCAAAAAAGGGAATAAGGGCGACACGGAAATGTTGAATACTCATACTCTTCCTTTTTCAATATTATTGAAGCATTTATCAGGGTTATTGTCTCATGAGCGGATACATATTTGAATGTATTTAGAAAAATAAACAAATAGGGGTTCCGCGCACATTTCCCCGAAAAGTGCCACCTGACGTCTAAGAAACCATTATTATCATGACATTAACCTATAAAAATAGGCGTATCACGAGGCCCTTTCGTCTCGCGCGTTTCGGTGATGACGGTGAAAACCTCTGACACATGCAGCTCCCGGAGACGGTCACAGCTTGTCTGTAAGCGGATGCCGGGAGCAGACAAGCCCGTCAGGGCGCGTCAGCGGGTGTTGGCGGGTGTCGGGGCTGGCTTAACTATGCGGCATCAGAGCAGATTGTACTGAGAGTGCACCATATGCGGTGTGAAATACCGCACAGATGCGTAAGGAGAAAATACCGCATCAGGCGCCATTCGCCATTCAGGCTGCGCAACTGTTGGGAAGGGCGATCGGTGCGGGCCTCTTCGCTATTACGCCAGCTGGCGAAAGGGGGATGTGCTGCAAGGCGATTAAGTTGGGTAACGCCAGGGTTTTCCCAGTCACGACGTTGTAAAACGACGGCCAGTGCCAAGCTTGCATGCCTGCAGGTCGACGATTCATAGTTGAACGTAATATACACACACGTCGTATCCAGTGCGAATACCTCGGACCCTGCACAATCTCTAGAGGATCCCCGGGTACCGAGCA

PtmiR93 5-TGTTTTGGGTGAAACGGGTGTT-3

RT-primer：5-GTCGTATCCAGTGCAGGGTCCGAGGTATTCGCACTGGATACGACAACACC-3

Forward primer： 5-CAGCGTGTTTTGGGTGAAACG-3

Amplicon was verified by sequencing

TTTACAAGGATTACGATTTCGAGCTCGGTACCCGGGGATCCTCTAGAGATTCAGCGTGTTTTGGGTGAAACGGGTGTT GTCATATCCAGTGCGAATACCTCGGACCCTGCACAATCGTCGACCTGCAGGCATGCAAGCTTGGCACTGGCCGTCGTTTTACAACGTCGTGACTGGGAAAACCCTGGCGTTACCCAACTTAATCGCCTTGCAGCACATCCCCCTTTCGCCAGCTGGCGTAATAGCGAAGAGGCCCGCACCGATCGCCCTTCCCAACAGTTGCGCAGCCTGAATGGCGAATGGCGCCTGATGCGGTATTTTCTCCTTACGCATCTGTGCGGTATTTCACACCGCATATGGTGCACTCTCAGTACAATCTGCTCTGATGCCGCATAGTTAAGCCAGCCCCGACACCCGCCAACACCCGCTGACGCGCCCTGACGGGCTTGTCTGCTCCCGGCATCCGCTTACAGACAAGCTGTGACCGTCTCCGGGAGCTGCATGTGTCAGAGGTTTTCACCGTCATCACCGAAACGCGCGAGACGAAAGGGCCTCGTGATACGCCTATTTTTATAGGTTAATGTCATGATAATAATGGTTTCTTAGACGTCAGGTGGCACTTTTCGGGGAAATGTGCGCGGAACCCCTATTTGTTTATTTTTCTAAATACATTCAAATATGTATCCGCTCATGAGACAATAACCCTGATAAATGCTTCAATAATATTGAAAAAGGAAGAGTATGAGTATTCAACATTTCCGTGTCGCCCTTATTCCCTTTTTTGCGGCATTTTGCCTTCCTGTTTTTGCTCACCCAGAAACGCTGGTGAAAGTAAAAGATGCTGAAGATCAGTTGGGTGCACGAGTGGGTTACATCGAACTGGATCTCAACAGCGGTAAGATCCTTGAGAGTTTTCGCCCCGAAGACGTTTTCCAATGATGAGCACTTTTAAAGTTCTGCTATGTGGCGCGGTATTATCCCGTATTGACGCCGGGCAAGAGCAACTCGGTTCGCCGCATACACTATTCTCAGAATGACTTGGGTTGAGTACTCACCAGTCACAGAAAGCATCCTTACCGGATGGCATGACAGTAGAGATTATTGCAGTGCTGCCATTACCAATGAAGTGAATAACACTGGCGGCCAACTTTACTTTCTGACAACCGAATCGAGGACGGATGGAGCCTTAACCGCTTTTTTGCACACATGGGGGAATCTTGTTACTCGCCTGATCGATTGGAACCGGAGACTTGATGTAAGCCATATACGTAGACGCAGCAGGCCCGT

MiR396 5-TTCCACGGCTTTCTTGAACTT-3

RT-primer：5-GTCGTATCCAGTGCAGGGTCCGAGGTATTCGCACTGGATACGACAAGTTC-3

Forward primer： 5-CAGCGTTCCACGGCTTTCTT-3

Amplicon was verified by sequencing

CGTTCGTATGGCTCATCAGTTCTCGATTCCACGATCAGGGAGGTACATGATCGCCATGTTGGCAAAAAAGCGGTAGCTCCTCGTCCTTCGATCGTGTCAGAGTAGGTGCGCAGTGTATCATCATGTATGCAGCAATGCATAGTTCTCTACTGTCATGCCATCGTAAGATGCTTTTCTGTGACTGGTGAGTACTCAACAAGTCATTCTGAGATAGTGTATGCGGGCGACCGAGTTGCTCTTGCCCGGCGTCAATACGGGATAATAACCGCGCCACATAGCAGAACTTAAAAGTGCTCATCATTGGAAAACGTCTTCGGGGCGAAAACTCTCAAGGATCTTACCGCTGTTGAGATCCAGTTCGATGTAACCCACTCGTGCACCCAACTGATCTTCAGCATCTTTTACTTTCACCAGCGTTTCTGGGTGAGCAAAAACAGGAAGGCAAAATGCCGCAAAAAAGGGAATAAGGGCGACACGGAAATGTTGAATACTCATACTCTTCCTTTTTCAATATTATTGAAGCATTTATCAGGGTTATTGTCTCATGAGCGGATACATATTTGAATGTATTTAGAAAAATAAACAAATAGGGGTTCCGCGCACATTTCCCCGAAAAGTGCCACCTGACGTCTAAGAAACCATTATTATCATGACATTAACCTATAAAAATAGGCGTATCACGAGGCCCTTTCGTCTCGCGCGTTTCGGTGATGACGGTGAAAACCTCTGACACATGCAGCTCCCGGAGACGGTCACAGCTTGTCTGTAAGCGGATGCCGGGAGCAGACAAGCCCGTCAGGGCGCGTCAGCGGGTGTTGGCGGGTGTCGGGGCTGGCTTAACTATGCGGCATCAGAGCAGATTGTACTGAGAGTGCACCATATGCGGTGTGAAATACCGCACAGATGCGTAAGGAGAAAATACCGCATCAGGCGCCATTCGCCATTCAGGCTGCGCAACTGTTGGGAAGGGCGATCGGTGCGGGCCTCTTCGCTATTACGCCAGCTGGCGAAAGGGGGATGTGCTGCAAGGCGATTAAGTTGGGTAACGCCAGGGTTTTCCCAGTCACGACGTTGTAAAACGACGGCCAGTGCCAAGCTTGCATGCCTGCAGGTCGACGATTCAGCGTTCCACGGCTTTCTTGAACTTGTCGTATCCAGTGCGAATACCTCGGACCCTGCACAATCTCTAGAGGATCCCCGGGTACCGAGCA

Csi-miR156 5-TGACAGAAGAGAGTGAGCAC-3

RT-primer 5-GTCGTATCCAGTGCAGGGTCCGAGGTATTCGCACTGGATACGACGTGCTC-3

Forward primer：5-CGGCGGTGACAGAAGAGAGT-3

Amplicon was verified by sequencing

ATTTGGACATGCATTTACGATTCGAGCTCGGTACCCGGGGATCCTCTAGAGATTCGGCGTGACAGAAGAGAGTGAGCACGTCGTATCCAGTGCGAATACCTCGGACCCTGCACAATCGTCGACCTGCAGGCATGCAAGCTTGGCACTGGCCGTCGTTTTACAACGTCGTGACTGGGAAAACCCTGGCGTTACCCAACTTAATCGCCTTGCAGCACATCCCCCTTTCGCCAGCTGGCGTAATAGCGAAGAGGCCCGCACCGATCGCCCTTCCCAACAGTTGCGCAGCCTGAATGGCGAATGGCGCCTGATGCGGTATTTTCTCCTTACGCATCTGTGCGGTATTTCACACCGCATATGGTGCACTCTCAGTACAATCTGCTCTGATGCCGCATAGTTAAGCCAGCCCCGACACCCGCCAACACCCGCTGACGCGCCCTGACGGGCTTGTCTGCTCCCGGCATCCGCTTACAGACAAGCTGTGACCGTCTCCGGGAGCTGCATGTGTCAGAGGTTTTCACCGTCATCACCGAAACGCGCGAGACGAAAGGGCCTCGTGATACGCCTATTTTTATAGGTTAATGTCATGATAATAATGGTTTCTTAGACGTCAGGTGGCACTTTTCGGGGAAATGTGCGCGGAACCCCTATTTGTTTATTTTTCTAAATACATTCAAATATGTATCCGCTCATGAGACAATAACCCTGATAAATGCTTCAATAATATTGAAAAAGGAAGAGTATGAGTATTCAACATTTCCGTGTCGCCCTTATTCTCTTTTTTGCGGCATTTTGCCTTCCTGTTGTTGCTCACCCAGAAACGCTGGTGAAAGTAAAAGATGCTGAAGATCAGTTGGGTGCACGAGTGGGTTACATCGAACTGGATCTCAACAGCGGTAAGATCCTTGAGAGTTTTCGCCCCGAAGAACGTTTTCCAATGATGAGCACTTTTAAAGTTCTGCTATGTGGCGCGGTATTATCCCGTATTGACGCCCGGGCAAGAGCAACTCGGTCGCCGCATACACTATTCTCAGAATGACGTGGTTTGAGTACTCACCAGTCACAGAAAAGCATCTTACTGATGGCATGACAGTAGAGATTATTGCAGTGCTGCATAACATGAGTGATAACACTGGCGGCACCTACTTCTGACACGATCGGAGGACGAAGAGCTACGCTTTTTTTGCACACATGGGGATCATGTAACTCCGCTGAATCGTTGGTAACGGCAGCTGAAATGAAGGCCATTACCCAACCGGACG

Csi-miR172 5-AGAATCTTGATGATGCTGCAT-3

RT-primer：5-GTCGTATCCAGTGCAGGGTCCGAGGTATTCGCACTGGATACGACATGCAG-3

Forward primer：5-CGGCCGAGAATCTTGATGATG-3

Amplicon was verified by sequencing

GAACTTTGAACATGATTACGATTCGAGCTCGGTACCCGGGGATCCTCTAGAGATTCGGCCAGAATCTTGATGATGCTGCATGTCGTATCCAGTGCGAATACCTCGGACCCTGCACAATCGTCGACCTGCAGGCATGCAAGCTTGGCACTGGCCGTCGTTTTACAACGTCGTGACTGGGAAAACCCTGGCGTTACCCAACTTAATCGCCTTGCAGCACATCCCCCTTTCGCCAGCTGGCGTAATAGCGAAGAGGCCCGCACCGATCGCCCTTCCCAACAGTTGCGCAGCCTGAATGGCGAATGGCGCCTGATGCGGTATTTTCTCCTTACGCATCTGTGCGGTATTTCACACCGCATATGGTGCACTCTCAGTACAATCTGCTCTGATGCCGCATAGTTAAGCCAGCCCCGACACCCGCCAACACCCGCTGACGCGCCCTGACGGGCTTGTCTGCTCCCGGCATCCGCTTACAGACAAGCTGTGACCGTCTCCGGGAGCTGCATGTGTCAGAGGTTTTCACCGTCATCACCGAAACGCGCGAGACGAAAGGGCCTCGTGATACGCCTATTTTTATAGGTTAATGTCATGATAATAATGGTTTCTTAGACGTCAGGTGGCACTTTTCGGGGAAATGTGCGCGGAACCCCTATTTGTTTATTTTTCTAAATACATTCAAATATGTATCCGCTCATGAGACAATAACCCTGATAAATGCTTCAATAATATTGAAAAAGGAAGAGTATGAGTATTCAACATTTCCGTGTCGCCCTTATTCCCTTTTTTGCGGCATTTTGCCTTCCTGTTTTTGCTCACCCAGAAACGCTGGTGAAAGTAAAAGATGCTGAAGATCAGTTGGGTGCACGAGTGGGTTACATCGAACTGGATCTCAACAGCGGTAAGATCCTTGAGAGTTTTCGCCCCGAAGAACGTTTTCCAATGATGAGCACTTTTAAAGTTCTGCTATGTGGCGCGGTATTATCCCGTATTGACGCCGGGCAAGAGCAACTCGGTCGCCGCATACACTATCTCAGATTGACTGCTGAGTACTCACCAGTCACAGACAGCATCTACGATGCATGACAGTAGAGATATGCATGCTGCATACATGATGAATAACACTGCCGCACCTACTTCGACACGATCGAGACGAAGAACCTAACCGCATTTGCAAACTGGATCCGTTACTCGCCCTTGATCGTTGGATCGGACCTA

Reverse primer: 5－GTGCAGGGTCCGAGGT－3
