# Supplementary material for: Identification and Comparative Profiling of miRNAs in an Early Flowering Mutant of Trifoliate Orange and Its Wild Type by Genome-Wide Deep Sequencing
Source: PLoS One. 2012 Aug 28;7(8):e43760. doi: 10.1371/journal.pone.0043760 (PMC3429500; doi:10.1371/journal.pone.0043760)
Supplement: Supporting Information S3 — Fold-back structures for conserved miRNA. Precursor secondary structures and dG value were produced using the mfold software. http://mfold.bioinfo.rpi.edu/. MiRNA and miRNA* sequences are highlighted in red and blue, respectively. The numbers along the structure are nucleotide sites from the 5′ end of the pre-miRNA sequence. (DOC) [file pone.0043760.s003.doc]

miR171i, scaffold_1:13073631:13073724, dG= -44.23

10 20 30 40 50

GUUGAAAUUUAAAAC-- A C UAU --| A

GGAGAUGUUGG ACGGCUCAAUCAAAU AAA CCUA UG A

CCUCUAUAACC UGCCGAGUUAGUUUA UUU GGGU AC A

UCGCUUUCUUCUGCUCU G A CCU UA^ U

110 100 90 80 70 60

sRNA (5' to 3' orientation) mapped to this predicted precursor hairpin: UGUUGGAACGGCUCAAUCAAA

miR160a, scaffold_1:4611422:4611604,dG= -68.54

10 20 30 40 50

AUAUA| C C U A C A

UAGUAUGC UGGCUCC UGUAUGCU UUUGC AAG CCAUCAG A

AUCGUACG ACCGAGG GCGUGCGA AGGCG UUC GGUGGUU G

UUUAA^ U A U G U A

. 90 80 70 60

sRNA (5' to 3' orientation) mapped to this predicted precursor hairpin: UGCCUGGCUCCCUGUAUGCUU

miR168a, scaffold_1:4611433:4611594,dG= -68.54

10 20 30 40 50

AUAUA| C C U A C A

UAGUAUGC UGGCUCC UGUAUGCU UUUGC AAG CCAUCAG A

AUCGUACG ACCGAGG GCGUGCGA AGGCG UUC GGUGGUU G

UUUAA^ U A U G U A

. 90 80 70 60

sRNA (5' to 3' orientation) mapped to this predicted precursor hairpin: UGCCUGGCUCCCUGUAUGCUU

miR168a, scaffold_1:4611433:4611594,dG= -68.54

10 20 30 40 50 60 70 80 90

AG--- GG UA C U A UG G UA UUUGAA| UU GAC AG GUGUU

UUACC CGGUCUC AUUCG UUGGUGCAGG CGGGA C AUU GC GUUUUUUU AUU UU AGCG GUGGC G

AGUGG GCCAGAG UAAGU AACUACGUUC GCCCU G UAA CG UAAAAAAG UAA AA UUGC UGCUG U

UAGAG A- GC C C A GU G -- UUAG--^ UU AU- AA GUUAA

180 170 160 150 140 130 120 110 100

sRNA (5' to 3' orientation) mapped to this predicted precursor hairpin: UCGCUUGGUGCAGGUCGGGAA

miR319b, scaffold_10:5361332:5361481,dG= -49.7

10 20 30 40 50 60 70 80 90 100

GUU UUU --- --| UU CU U AA AG- G AC UC AA A AAAGG GA

GAU GAUUA AGC UGGGAGCU CUUCGGUCCA UAUGGG GGC UAGGAUUUAAUU CU CUG UCAUUCA CA UACUG GU GUC A

CUA CUAAU UCG ACCCUCGA GAAGUCAGGU GUGUCC UCG AUUCUAAGUUAA GA GGC AGUAAGU GU AUGAC CA UAG A

C-- UU- CGC UU^ GG UC U A- ACA G GU GA AA C ----- GU

200 190 180 170 160 150 140 130 120 110

sRNA (5' to 3' orientation) mapped to this predicted precursor hairpin: GAGCUUUCUUCGGUCCACUU

miR157b, scaffold_138:158623:158752,dG= -53.02

10 20 30 40 50

-| GAUG - U - UCUAAU

GGU CUGGUGACAGAAG AUAGAGAGCGCAGA GA UGAUAUGC \

CCG GACUACUGUCUUC UGUCUCUCGUGUUU CU ACUAUAUG G

A^ ---- G C C UUUGAG

100 90 80 70 60

sRNA (5' to 3' orientation) mapped to this predicted precursor hairpin: GUGACAGAAGAUAGAGAGCGC

miR168a, scaffold_1:4611433:4611594,dG= -68.54

10 20 30 40 50

AUAUA| C C U A C A

UAGUAUGC UGGCUCC UGUAUGCU UUUGC AAG CCAUCAG A

AUCGUACG ACCGAGG GCGUGCGA AGGCG UUC GGUGGUU G

UUUAA^ U A U G U A

. 90 80 70 60

sRNA (5' to 3' orientation) mapped to this predicted precursor hairpin: UGCCUGGCUCCCUGUAUGCUU

miR169h, scaffold_15:3899913:3900035,dG= -57.07

UU ----| U U - - UUGAUU C G AG

GUUUGU UAGCCAAGGA GACU GCCUG CA CC GCAAGA GAGG UUUCA U

CGGACA AUCGGUUCCU CUGA CGGAC GU GG UGUUCU CUCC AAGGU G

CU CUGA^ - - C U UU---- - G CG

110 100 90 80 70 60

sRNA (5' to 3' orientation) mapped to this predicted precursor hairpin: UAGCCAAGGAUGACUUGCCUGCA

miR396b, scaffold_159:106629:106730,dG= -56.01

10 20 30 40 50 60 70

AUUAA ------- C A CU U --| A A C U CU

GUCCUG GUCAUG UUUUCCACAGCUUUCUUGA CUUCCA GU UGC UG UU AUA ACGGC CUUG \

UAGGAC CGGUAC AGAGGGUGUCGAAAGAACU GAGGGU CG GCG AC AG UGU UGCCG GGAU A

----- UUCCACA A C AC C CC^ - A A C CG

150 140 130 120 110 100 90 80

sRNA (5' to 3' orientation) mapped to this predicted precursor hairpin: UUCCACAGCUUUCUUGAACUU

miR393a, scaffold_16:4058207:4058329,dG= -49.4

10 20 30 40 50

UUCU-- A UA UA C U AA---| C U AAU

GC AC GAGGA AAUCCAAAGGGAU GCA UGAUCCU GC UUAA UAU \

CG UG UUCCU UUAGGUUUCCCUA CGU ACUAGGA UG AAUU AUA U

UAUACU A GC CC U - GUACU^ U C CUC

120 110 100 90 80 70

sRNA (5' to 3' orientation) mapped to this predicted precursor hairpin: UCCAAAGGGAUCGCAUUGAUC

miR396h, scaffold_4:4045478:4045588,dG= -49.4

10 20 30 40 50

C G C C A C A AA-- CAU--| AG

CU UUUGUAU UU CCACAGCUUU UUGAAC GCA CA UGC GCCA U

GA AAACAUA AA GGUGUCGAAA AACUUG CGU GU ACG CGGU C

- G A A G A A AGAA UACUC^ GU

110 100 90 80 70 60

sRNA (5' to 3' orientation) mapped to this predicted precursor hairpin: UCCCACAGCUUUAUUGAACC

miR160a, scaffold_2:79290:79377,dG= -50.7

10 20 30 40 50 60

--- U AUU--| GU C C A A A- GC

AAGG GAA AAUGG UAUGC UGGCUCC UGUAUGCCGC AGCAG CG CAAUCU \

UUCC CUU UUAUC AUACG ACCGAGG GCAUGCGGUG UCGUU GC GUUAGA C

UCU - CCUUC^ -- A A G - CG GG

120 110 100 90 80 70

sRNA (5' to 3' orientation) mapped to this predicted precursor hairpin: UGCCUGGCUCCCUGUAUGCCG

miR393, scaffold_20:1575655:1575774,dG= --59.8

10 20 30 40 50 60 70

UGA U --- A A U U AACAUU| ------------- - A

UUAG GC AGGUGG GAGUUCC AAGGGAUCGCA UGAUCUGA GAU AAAUUA AUCA CUC A

AAUC CG UCUACC CUUAAGG UUCCCUAGCGU ACUAGAUU UUA UUUAGU UAGU GGG A

UA- U GUA C C - U CUUUC-^ AUAUAUAUUAUUU U C

150 140 130 120 110 100 90 80

sRNA (5' to 3' orientation) mapped to this predicted precursor hairpin: UUCCAAAGGGAUCGCAUUGAUC

miR156a, scaffold_25:3136148:3136285,dG= -49.1

10 20 30 40 50

AACAUU- A - - A GU-| A

GAA UUGACAG AAGAG AGUGAGCAC CAGAGGCA UGUAUA U

CUU GACUGUC UUCUC UCACUCGUG GUUUUCGU ACAUAU U

CCAUGAC C U G C UGU^ G

100 90 80 70 60

sRNA (5' to 3' orientation) mapped to this predicted precursor hairpin: UUGACAGAAGAGAGUGAGCAC

miR396g, scaffold_27:1236405:1236538,dG= -49.6

10 20 30 40 50 60 70

----| GU UG C U A AAUUA - UUUU C CAA

UAAAG CUUU CAUG UUUUCCACGGC UUCUUGA CUU GUAC GGUUAA UUUGUG UAUUAA \

GUUUU GAAA GUAC AAAGGGUGCCG AAGAACU GAA CGUG CUAAUU AAACGU AUAAUU G

UGCU^ UG -- A U C A---- A UCU- - AAC

. 140 130 120 110 100 90 80

sRNA (5' to 3' orientation) mapped to this predicted precursor hairpin: UUCCACGGCUUUCUUGAACUU

miR162, scaffold_29:814051:814181,dG= -52.7

10 20 30 40 50 60

-- A--| A CA G C C AC UG CAA U GAA

AUAGAG GAGUGA GU CUGGA GCAG GGUU AUCGAUC UU UG AUUUUGU GU A

UAUCUC CUCACU CG GACCU CGUC CCAA UAGCUAG AA AC UAAAACA CA A

CU AAC^ - C- A U A CU GU A-- - AUA

. 120 110 100 90 80 70

sRNA (5' to 3' orientation) mapped to this predicted precursor hairpin: GGAGGCAGCGGUUCAUCGAUC

miR171f, scaffold_39:428372:428489,dG= -38.90

10 20 30 40 50

AAAAAACGA C A C A - GA- -| AU

AAG GAUAUUGGUG GGUUCAAUC G AGA CG UUUAC AC U

UUC CUAUAACCGC CCGAGUUAG C UCU GC AAAUG UG G

AAUAUUUAA A G A - A AGA U^ AA

100 90 80 70 60

sRNA (5' to 3' orientation) mapped to this predicted precursor hairpin: CGAUAUUGGUGAGGUUCAAUC

miR398b, scaffold_4:4081925:4082032,dG= -69.90

10 20 30 40 50 60

G A U GC-| UU - AA U AU

AGAAGUCCCGCAGGGGCGAC UGAGA CACAU AACGCA GC CU UGCC AUAC \

UCUUUAGGGUGUCCCCGCUG ACUCU GUGUG UUGUGU UG GA GUGG UGUG U

- G U UAU^ U- U GG - GU

. 110 100 90 80 70

sRNA (5' to 3' orientation) mapped to this predicted precursor hairpin: GGGGCGACAUGAGAUCACA

miR170, scaffold_4:4121669:4121797,dG= -60.70

10 20 30 40 50 60

AU--- U C U C C ---------- -| UUUA U

AUGAAAGGG AG CG GAUAUUGGC UGGUUCA UCAGAC AAACC GG UUUGCUUU A

UACUUUCUC UU GC CUAUAACCG GCCGAGU AGUUUG UUUGG CC AAACGAGA U

CUUUU U - U U U GUUUUGUUUU A^ UUA- U

130 120 110 100 90 80 70

sRNA (5' to 3' orientation) mapped to this predicted precursor hairpin: UAUUGGCCUGGUUCACUCAGA

miR156f, scaffold_44:542896:542995,dG= -59.80

10 20 30 40 50 60

GUAAAAA------ AAGA A - A AUU| UU UGCUUU

GGGU GG GGUGACAGA AGAGAGUGAGCAC CAUGGU UUC GCA \

CCCA CC CCACUGUCU UCUCUCACUCGUG GUAUCG AAG CGU G

AACCCCCCGCCCC A--- - A C ---^ UU CCUAGA

120 110 100 90 80 70

sRNA (5' to 3' orientation) mapped to this predicted precursor hairpin: UGACAGAAGAGAGUGAGCAC

miR172e, scaffold_6:4465828:4465981,dG= -59.50

10 20 30 40

UGCUGC- - GGU A .-A| U

CA AUAUUUGC UGCGGCAUCAUCAAGAUUC CA GC U

GU UAUAAACG ACGUCGUAGUAGUUCUAAG GU CG U

AGCAUCA A ACU G \ -^ A

130 120 110 100 50

60 70

A------- CA G

AGGGAAUU UUGAAUA G

UUCCUUGA AACUUAU C

AAUAAACC A- C

90 80

sRNA (5' to 3' orientation) mapped to this predicted precursor hairpin: GCGGCAUCAUCAAGAUUCACA

miR157a, scaffold_63:1103294:1103397,dG= -49.50

10 20 30 40 50

---------| AU UC UGCUGU - U - A UGG

AGGC UG GAUGA UGACGGAAGA UAGAGAGCACAGA GA UGA AUGCG \

UCCG AC CUACU ACUGUCUUCU AUCUCUCGUGUUU CU ACU UACGU A

AUUUCCCUC^ -- UU ------ U C C C UCG

120 110 100 90 80 70

sRNA (5' to 3' orientation) mapped to this predicted precursor hairpin: UUGACGGAAGAUAGAGAGCAC

miR166b, scaffold_8:3530395:3530520,dG= -58.90

10 20 30 40 50 60

AAA -- U C UU CU AUUA-----| A A

AAUGA UUG UUCUGAGGGGA UG GUCUGG CGAUGC AU AUAAUUAUAAUU U

UUACU AAC AAGACUUCCCU AC CGGACC GCUAUG UA UAUUAAUAUUAA A

UUG UA U U UU AG GGAAAAAAG^ C U

130 120 110 100 90 80 70

sRNA (5' to 3' orientation) mapped to this predicted precursor hairpin: GGACUGUUGUCUGGCUCGAUG

miR390a-3p, scaffold_80:25061:25182,dG= -53.40

10 20 30 40 50 60

AA--- A U U G AUG ----------- UA--| AA

AGU AAGAAGAA CUGU AAGCUCAGGA GGAUAGCGCC GGUGCC AUGAA UGGG \

UCA UUCUUCUU GGUA UUUGAGUCCU CCUAUCGCGG UCACGG UACUU AUCU U

CUCUC - C C A --- GUAAUAAAUAG UGGG^ AG

130 120 110 100 90 80 70

sRNA (5' to 3' orientation) mapped to this predicted precursor hairpin: AAGCUCAGGAGGGAUAGCGCC

miR172a, scaffold_95:631662:631778,dG= -57.40

10 20 30 40 50 60

GAC-- GU G A - AAAUACACG -| U GU

AGUCGCU UUGC GGUGUAGCAUCAUCAAGAUUC CA UGC GC AG GAU G

UCGGCGA AACG CUACGUCGUAGUAGUUCUAAG GU ACG CG UC CUA A

AAAAU AU A A C AAG------ U^ U AU

120 110 100 90 80 70

sRNA (5' to 3' orientation) mapped to this predicted precursor hairpin: GUAGCAUCAUCAAGAUUCAC

miR169g, scaffold_95:696867:696987,dG= -47.10

10 20 30 40 50 60

G| AGAAA U - U U C --- U -- GG GAGUA

AGG UGUU GUU AGCCAAGGA GACU GCCUG GU CA GCA AGAG UUUC \

UCC ACAG CGA UCGGUUCCU CUGA CGGAC CG GU UGU UCUC AAAG G

-^ G---- U A - - - UUG U CA GA AUAUC

120 110 100 90 80 70

sRNA (5' to 3' orientation) mapped to this predicted precursor hairpin: AGCCAAGGAUGACUUGCCUGCG
