# Supplementary material for: Identification and Comparative Profiling of miRNAs in an Early Flowering Mutant of Trifoliate Orange and Its Wild Type by Genome-Wide Deep Sequencing
Source: PLoS One. 2012 Aug 28;7(8):e43760. doi: 10.1371/journal.pone.0043760 (PMC3429500; doi:10.1371/journal.pone.0043760)
Supplement: Supporting Information S4 — Fold-back structures for novel miRNA. Precursor secondary structures and dG value were produced using the mfold software. http://mfold.bioinfo. rpi.edu/. MiRNA and miRNA* sequences are highlighted in red and blue, respectively. The numbers along the structure are nucleotide sites from the 5′ end of the pre-miRNA sequence. (DOC) [file pone.0043760.s004.doc]

Novel01, scaffold_109:95168:95314, dG = -40.70

10 20 30 40 50 60 70

- UG U UC AA- CUGA UUACACAUAUUUCAUU| A AACA

U UAAUGUUUCU GCUCAAAUGAGUAU CA AACAG AUC UAU AGAA \

A AUUAUGAAGA CGAGUUUACUCAUA GU UUGUC UAG AUA UCUU A

G GU U GA CGG UUCG UUACUUCUCCUU----^ C AUAG

140 130 120 110 100 90 80

sRNA (5' to 3' orientation) mapped to this predicted precursor hairpin:UCUUGCUCAAAUGAGUAUUCCA

Novel02, scaffold_10:2672216:2672314, dG = -52.50

10 20 30 40

U A - CCA-----| U U

ACCAUAGU GUACCAUACCACAGCUGGAUUCA GC CA UUAU A

UGGUAUCA UAUGGUAUGGUGUCGACUUAGGU CG GU AAUA U

- C C UAAUCUAG^ U U

90 80 70 60 50

sRNA (5' to 3' orientation) mapped to this predicted precursor hairpin: CCAUACCACAGCUGGAUUCAGCC

Novel03, scaffold_10:4971000:4971152, dG=-61.90

10 20 30 40 50 60 70 80

AGAUA--- U U - GCUU-| AA UC UCAAAUUUAGCUU CA

AAGAGC UUGUU GA UAGCCAAGGAUGACUUGCCUU CUG GCAAGG UC AGCUG U

UUCUCG GACAA CU AUCGGUUUCUGCUGAACGGAG GAU CGUUCC AG UCGAU A

ACUUAUUA - U G UCUUU^ C- UA C------------ AU

150 140 130 120 110 100 90

sRNA (5' to 3' orientation) mapped to this predicted precursor hairpin: UAGCCAAGGAUGACUUGCCU

Novel04, scaffold_15:2215354:2215608, dG=-91.73

10 20 30 40 50 60 70 80 90

G G A UA .-AUCAAUCAAG UC UUU- UG --- C-| UC

UAUGUUUUGU CUAACGUUGACC GUUGCAC GUCAAAUAAGAUUAUCAA CUGU UAUUGA UGGG GA UAC UGGU \

AUACAAGACA GAUUGUAACUGG UAACGUG CAGUUUAUUCUGAUAGUU GACG AUGAUU ACCU CU GUG AUCA A

- G A UA \ ---------- UA UGUU GU UAC AA^ UC

250 240 230 220 210 130 120 110 100

140 150 160

AUAC---------- GUAUUAAAAAAAUAU

UCAUAGUUUC A

AGUGUUAAGG A

ACUUUUUUAACCUA AAACUAUUGGAAAAA

200 190 180 170

sRNA (5' to 3' orientation) mapped to this predicted precursor hairpin:UAACGUUGACCAGUUGCACUAGU

Novel05, scaffold_19:1984361:1984493, dG= -63.00

10 20 30 40 50 60

G GA UC AGA ---------- AC-- -| A

AG GAAU CAGGGCAAUUCUCCUUUGGC AAAUAUU GCGCUUGU GUAC ACGC \

UC CUUA GUCCCGUUAAGAGGAAACCG UUUAUAA CGCGGACA CAUG UGUG C

- AA CC CCG UAUAUAAUAU CGUU A^ G

130 120 110 100 90 80 70

sRNA (5' to 3' orientation) mapped to this predicted precursor hairpin: GGGCAAUUCUCCUUUGGCAGA

Novel06, scaffold_1:13086671:13086815, dG= -57.90

10 20 30 40 50 60 70

A| AA AAA - CU CUU- CGAUCUA U GUUCA

AGAG GAAG GAAGUG AGGAAAUUU GGAAUGGGAGG GGCAAGAAG UUU UCUU \

UCUC CUUC CUUUAC UCCUUUAGG CCUUACCCUCC CCGUUCUUU AAA AGAA A

-^ -- --- G UU CCAC UUAAAAG U AAAAU

140 130 120 110 100 90 80

sRNA (5' to 3' orientation) mapped to this predicted precursor hairpin: UGAGGAAAUUUCUGGAAUGGG

Novel07, scaffold_22:3381361:3381439, dG= -34.20

10 20 30

ACC C--| U G A UC G

GAAGG AUCC UGGG UGAUCUCGU GU CUAC \

CUUCC UAGG ACCU GCUGGGGUA CA GGUG A

UUC UUU^ U G G GA G

70 60 50 40

sRNA (5' to 3' orientation) mapped to this predicted precursor hairpin:UCCUUGGGGUGAUCUCGUAGU

Novel08, scaffold_27:847685:847792, dG=-70.70

10 20 30 40 50

U C U----| ACC

AACUGUGGUACCGUGCCACAGUUGCAUCCAGU GUUGGAUGU AGUAG \

UUGACACUAUGGCACGGUGUUAAUGUAGGUCG CAACUUACA UUAUC C

- A UGAAU^ CAC

100 90 80 70 60

sRNA (5' to 3' orientation) mapped to this predicted precursor hairpin:GUGCCACAGUUGCAUCCAGUC

Novel09, scaffold_27:1650451:1650560, dG= -73.10

10 20 30 40 50

GGGA| A GG AC GGA

UUGUGCUGGCCCCAAUCCGUGGACAAAGGAAAAUU GUA AAC UG U

AACACGACCGGGGUUAGGCACCUGUUUCCUUUUAA CGU UUG AC U

AAAG^ - A- GU AAU

. 100 90 80 70 60

sRNA (5' to 3' orientation) mapped to this predicted precursor hairpin: GCCCCAAUCCGUGGACAAAGG

Novel10, scaffold_2:3455812:3455930, dG=-44.10

10 20 30 40 50

U---| A UA U U U A UUU UUGG

UUUUA UUUUCUGUUAUG GCU GAG CU GCUGAAAGUAC UG AAAAGU \

AAAGU GAGAGACGAUAC CGG CUC GA CGACUUUCAUG AC UUUUUA A

UAUU^ - UG C C - A CU- UUUU

110 100 90 80 70

sRNA (5' to 3' orientation) mapped to this predicted precursor hairpin:AGCUUGAGUCUUGCUGAAAGUA

Novel11, scaffold_2:6255278:6255381, dG=-44.20

10 20 30 40 50

A| CCCAU C G C C UGG

CCUUU GCUU UUUUUGCUACUUCUACUGG AU UUUUUU CCU A

GGAAG CGAA AGAAACGAUGAAGGUGAUC UA AAAAAA GGA A

-^ UGUCU A G - C UUG

100 90 80 70 60

sRNA (5' to 3' orientation) mapped to this predicted precursor hairpin: UUCUUUUUGCUACUUCUACUG

Novel12, scaffold_64:1246869:1246958, dG = -34.40

10 20 30 40

-| C U UGA A U- GGUC

CUUU ACUCUUUU GUUGCAUGAUGC UA UGAA GUA \

GAAG UGAGAAAA CAACGUACUACG AU ACUU CAU A

U^ U - CCC C UU AGUU

. 80 70 60 50

sRNA (5' to 3' orientation) mapped to this predicted precursor hairpin:UUUUGUUGCAUGAUGCUGAUAA

Novel13, scaffold_73:771564:771820, dG = -79.30

10 20 30 40 50 60 70 80 90

AUC-- UG UUCCAA- CU UU CU AUUA- CU --- .-UUCUUCC| C CUU A

CUUG CAGG GGCAAGUCGU UUGGCUAUU GACAGG CUU UUCAUG AGGCUU UCUC ACAG CA CAA U

GAAU GUCU CCGUUCAGUA AACCGAUAG UUGUUU GAA AAGUAC UCUGAG AGAG UGUC GU GUU U

GGUGU GA UCCUCGC AG U- U- GUUAG AU AAA \ -------^ C U-- C

250 240 230 220 210 200 190 180 110 100

120 130 140

---- AU- G AAU-- C C

CGC GU UUCCUUAAA UA ACAGU A

GCG UA AAGGAGUUU AU UGUCA A

UUUU CAG G ACGUU A C

170 160 150

sRNA (5' to 3' orientation) mapped to this predicted precursor hairpin:GCAAGUCGUCUUUGGCUAUUU

Novel14, scaffold_73:771772:771902, dG = -54.60

10 20 30 40 50 60 70

C U- CUUUUAAAGA| UCUCUCAACUUUA U CA

CUUGUU GAUAGCCAAGGAUGACUUGCCUUG AAGG GCU AGCUA U

GGACAG UUAUCGGUUUCUGCUGAACGGAAC UUCC UGA UCGAU A

C UU CUUGGACGUG^ UA----------- - UU

. 120 110 100 90 80

sRNA (5' to 3' orientation) mapped to this predicted precursor hairpin:UAGCCAAGGAUGACUUGCCU

Novel15, scaffold_82:519444:519576, dG=-69.90

10 20 30 40 50 60

UCAAC C CUUACU G UUAA-| A

UAUGUUGCAACUGUGGUA GGUACCACA UUCA UCAUUGGAUCUAC UGC A

AUACAACGUUGACACCAU CCAUGGUGU AGGU GGUAACUUAGAUG AUG U

UUUUA A CAACGU G UCAAC^ U

130 120 110 100 90 80 70

sRNA (5' to 3' orientation) mapped to this predicted precursor hairpin: UGCAACUGUGGUACGGUACCA

Novel16, scaffold_83:437489:437629, dG = -56.00

10 20 30 40 50 60

-----| A UU UGC A A CU- CCA U- AAAUA

GAUGA GAGCU GCUU UAGCC AGGAUG CUUGC ACGU UCAU GAGGGUUUUCA A

CUAUU CUCGG UGAG AUCGG UCCUAC GAACG UGUA GGUG UUCCUAAGAGU A

ACUUU^ - -- UUA G - AGU UUG UU AAGAA

. 130 120 110 100 90 80 70

sRNA (5' to 3' orientation) mapped to this predicted precursor hairpin:UAGCCAAGGAUGACUUGCCU

Novel17, scaffold_8:587742:587859, dG=-64.80

10 20 30 40 50 60

G| AGUG ACAG U UG UG

GCC CUAUGUUACGUUGAACGUAAUAUACACACACUC CAACAAUA UAA G G

UGG GAUACAAUGCAACUUGCAUUGUAUGUGUGUGAG GUUGUUAU AUU U G

-^ ---- A--- C GU CG

110 100 90 80 70

sRNA (5' to 3' orientation) mapped to this predicted precursor hairpin: GUUGAACGUAAUAUACACACA

Novel18, scaffold_68:754320:754401, dG = -24.50

10 20 30 40

UUG CA-| C UA AG UA

ACACUA ACCCAUAUCA AUAAU AAAUU UUG C

UGUGAU UGGGUAUAGU UGUUG UUUAA AAC U

UGA AAC^ - -- AG UC

80 70 60 50

sRNA (5' to 3' orientation) mapped to this predicted precursor hairpin:ACCCAUAUCACAUAAUUAAAAUUA

Novel19, scaffold_95:743113:743226, dG=- 51.20

10 20 30 40 50

- A U - U U AU- U UA U -------| A

GCU GUU GG UAGCCAAGGA GAC UGCCUG CUC CC AG GGU UUCAA A

CGA CAG UC AUCGGUUCCU CUG ACGGAC GGG GG UC CCA AAGUU C

C - U A - - CAU U GC - AAUUUAU^ G

110 100 90 80 70 60

sRNA (5' to 3' orientation) mapped to this predicted precursor hairpin: UAGCCAAGGAUGACUUGCCU

Novel20, scaffold_118:430222:430304, dG= -21.60

10 20 30

UUAUA -----| A A A GC - A

GAU ACAUGG GUAA UC UG CGUC GGAUUA A

CUG UGUACC CGUU AG AC GCAG UUUGAU A

GACAA AUUAC^ G - G UC G A

80 70 60 50 40

sRNA (5' to 3' orientation) mapped to this predicted precursor hairpin: AUGGAGUAAAUCAUGGCCGUCGG

Novel21, scaffold_128:225135:225278, dG = -89.60

10 20 30 40 50 60 70

- AAAA U -| UACACU CAU

AGGGU CUAUGUUGCAACUGUGGUAUGGUAUCACAGUUGUUUUC GCCGUUGGA UC UG A

UCCCA GAUACAACGUUGACACCAUACCAUGGUGUCAACGAAAG CGGUAAUCU AG AC A

A GGUC U A^ UCAAUU AUU

140 130 120 110 100 90 80

sRNA (5' to 3' orientation) mapped to this predicted precursor hairpin:UAUGUUGCAACUGUGGUAUGGUA

Novel22, scaffold_129:473882:473988, dG= -23.30

10 20 30 40 50

AU--| GACG GCA G AGG A - GG AA UC

GAU UG UU ACUGAUG UGUCACA UC CUAUU GCUA AU U

CUA GC AG UGACUAU ACGGUGU AG GGUAG UGGU UG U

CGCU^ AAAA --- G ACG - U -- CA UA

100 90 80 70 60

sRNA (5' to 3' orientation) mapped to this predicted precursor hairpin: AUUGACUGAUGAGGUGUCACAAU

Novel23, scaffold_12:4547645:4547798, dG= -92.10

10 20 30 40 50 60 70

-| G A A A A G

AGUUUUCUAGUUCGGGAUUUUAAAGUGCGGGAAAGUCUGAAAAA CUUUAAAACC UGC GUUUUGA GC UUAG A

UUAAAAGGUUAGGCCCUAAAAUUUCACGCUCUUUCAGACUUUUU GAAAUUUUGG ACG CAAAAUU CG AAUU A

U^ - G C A A A

150 140 130 120 110 100 90 80

sRNA (5' to 3' orientation) mapped to this predicted precursor hairpin: UUCGGGAUUUUAAAGUGCGGG

Novel24, scaffold_14:4093405:4093478, dG=- 19.20

10 20 30

C U---- UUG C---| U CC

CAUA GUCUGUGGA GAUG GGAU UGA C

GUAU UAGACAUUU CUAC CCUA ACU A

A UUAAU UA- CCAA^ U UA

70 60 50 40

sRNA (5' to 3' orientation) mapped to this predicted precursor hairpin: GUGGAUUGGAUGCGGAUUUGA

Novel25, scaffold_15:3899911:3900025, dG = -55.00

10 20 30 40 50 60

A| UGAAAUUA GU C C A A A- GC

GAAGG AUGG UAUGC UGGCUCC UGUAUGCCGC AGCAG CG CAAUCU \

CUUCU UAUC AUACG ACCGAGG GCAUGCGGUG UCGUU GC GUUAGA C

C^ -------- -- A A G - CG GG

110 100 90 80 70

sRNA (5' to 3' orientation) mapped to this predicted precursor hairpin: UGCCUGGCUCCCUGUAUGCCG

Novel26, scaffold_16:1864426:1864631, dG= -140.00

10 20 30 40 50 60 70 80 90

G - G A-- .-A| C GAC

GUGCCAACUUUGUCAUGCAAUUGUAGUCAAAGUCUACAUGCUAGCUCAACAAUUGCAUGCUUGAA UUUG AU GUG UUCU CAUC \

CACGGUUGAAACAGUACGUUAACAUCAGUUUCGGAUGUACGAUCGAGUUGUUAACGUACGAACUU AAAC UA CAC AAGA GUAG C

- A G CGA \ -^ A GAA

200 190 180 170 160 150 140 130 100

110

GA GA

GAGGUUCU C

UUCCAAGA C

-- AC

120

sRNA (5' to 3' orientation) mapped to this predicted precursor hairpin: UCAUGCAAUUGUAGUCAAAGU

Novel27, scaffold_18:88175:88313, dG= -49.30

10 20 30 40 50

A U UCUU C UU A GC .-C| UUA

GC GU UUGAGGGGA UG GUCUGGUUC AG CAU AAUUAA U

CG CA AAUUCCCCU AC CGGACCAGG UC GUG UUAGUU A

- U UU-- U UU C UU \ -^ UCA

130 120 110 60

70

----- G A

CA GUGUGUGUGUGU U

GU UAUAUAUAUAUA G

UGUGU G U

100 90 80

sRNA (5' to 3' orientation) mapped to this predicted precursor hairpin: GGGACUGUUGUCUGGUUCAAGG

Novel28, scaffold_1:4611433:4611594, dG = -59.90

10 20 30 40 50 60 70 80

- UA C U A UG G UA UUUGAA| UU GAC AG GUGUU

GGUCUC AUUCG UUGGUGCAGG CGGGA C AUU GC GUUUUUUU AUU UU AGCG GUGGC G

CCAGAG UAAGU AACUACGUUC GCCCU G UAA CG UAAAAAAG UAA AA UUGC UGCUG U

G GC C C A GU G -- UUAG--^ UU AU- AA GUUAA

160 150 140 130 120 110 100 90

sRNA (5' to 3' orientation) mapped to this predicted precursor hairpin:UCGCUUGGUGCAGGUCGGGAA

Novel29, scaffold_27:1236405:1236538, dG = -46.70

10 20 30 40 50 60

U U A U CAU-- UGA-| U U CUG AAU U

UAUAUUUG UUGG CAGAGAAA CACGGU GAGA GUU UC AAU UACUGU CUG A

AUAUAGAC AACC GUCUCUUU GUGCCA CUCU CAG AG UUA GUGGUA GGC U

U U C - UCUUU UAGG^ C - UAA GU- A

130 120 110 100 90 80 70

sRNA (5' to 3' orientation) mapped to this predicted precursor hairpin:UUGGACAGAGAAAUCACGGUCA

Novel30, scaffold_2:4942371:4942595, dG= -99.60

10 20 30 40 50

- C - C .-A| A

AUCC ACUCCAAGCC AAAAUGACAC CUUUCCUAGCGUUUUUGGAAACCAA UUUC A

UAGG UGAGGUUCGG UUUUGCUGUG GAAAGGGUCGCAAAAACCUUUGGUU AAAG G

G U U A \ -^ U

220 210 200 190 180 60

70 80 90 100 110 120

AAU GCA AUAA A GCU UAGGAU A-- ACU AU

GGCU CAA AGG UAG GUUG GAGU UCAUU CUUU \

UCGA GUU UCC AUU CAAC CUCA GGUAA GAAA G

C-- AA- AG-- - AU- UUUAAU AAG AUU AU

170 160 150 140 130

sRNA (5' to 3' orientation) mapped to this predicted precursor hairpin: AAGCCAAAAUGACACCCUUUCCU

Novel31, scaffold_30:2196438:2196578, dG= -34.60

10 20 30 40 50 60

CCCAAAACA AGU U ---------- CU UCUA| CG CA G

AUCAGAGA AAGAUAU UCCUUGG GCAUG GGUU UAGA UUU UUAAA \

UAGUUUCU UUCUAUG AGGAAUC UGUAU CCAG AUCU GAA GAUUU A

UGUACUUAG GUU U GUUCAUUUAA UC ----^ UU AC U

. 130 120 110 100 90 80 70

sRNA (5' to 3' orientation) mapped to this predicted precursor hairpin: AGAGAAGUAAGAUAUUUCCUUGG

Novel32, scaffold_39:1391080:1391195, dG = -56.40

10 20 30 40 50

GAU- G -| CC A AAA GAUUAC

AAGAGUAAG AUU GAGGCGCAC CAGCGUGG ACCAUCA GUUGU \

UUCUUAUUC UAA UUCUGCGUG GUCGCACC UGGUAGU UAACG A

GCGU G G^ U- G GGG AGAUUG

110 100 90 80 70 60

sRNA (5' to 3' orientation) mapped to this predicted precursor hairpin:CGCACCCCAGCGUGGAACCAUC

Novel33, scaffold_39:1799757:1799942, dG = -69.70

10 20 30 40 50 60 70

GGAGA - U --- C UG CA CUUUCCUA| C .-AG AUAU

UGAA AUGA GC GGUG AGCCAAGGA ACUUGCCGG AGCCC GAA CUA CAUC \

ACUU UACU CG CCAC UCGGUUUCU UGAACGGCU UUGGG CUU GAU GUAG U

AC--- G C UCU A GU AA AUGUUCA-^ C \ -- AUUU

180 170 160 150 140 130 80

90 100

GCU-- C UA U

UCA AAU UGAGUC U

AGU UUG GUUCAG U

AUUUU - GG U

1. 110

sRNA (5' to 3' orientation) mapped to this predicted precursor hairpin: CAGCCAAGGAUGACUUGCCGG

Novel34, scaffold_3:6400070:6400194, dG= -45.80

10 20 30 40 50 60

G AA A C --| U CGCAUUU AAA AUAAAUAA

GGGAA AGUGA GCUG C AGCAUGAUCUA CUAG GGUUAGUG GUA \

UCCUU UCACU CGAC G UCGUACUGGAU GAUC CCAAUCAU CAU A

A CC C A UC^ C ------- AG- GAACGAAG

120 110 100 90 80 70

sRNA (5' to 3' orientation) mapped to this predicted precursor hairpin: UGAAGCUGCCAGCAUGAUCUA

Novel35, scaffold_49:246191:246294, dG = -40.40

10 20 30 40 50

AUA GCU AG ACCU| --- AUUCG AA

CAAAUUU UGACAAUG AGAGAGCAC GU CAGC UGC \

GUUUAAA ACUGUUAC UCUCUCGUG CA GUCG ACG A

UUC AU- CA CGAU^ UCU GA--- GU

100 90 80 70 60

sRNA (5' to 3' orientation) mapped to this predicted precursor hairpin:UGACAAUGAGAGAGAGCACAC

Novel36, scaffold_51:957676:957782, dG= -76.20

10 20 30 40 50

-| U A UUAAAA A

UCAGCUGA CUUGUGCUGAGCACCGGAUCAAU GUCAGCAAUUAU CUC C

AGUCGACU GAACACGACUCGUGGCCUAGUUA CAGUUGUUAAUG GAG A

G^ C C ------ U

100 90 80 70 60

sRNA (5' to 3' orientation) mapped to this predicted precursor hairpin:UUGUGCUGAGCACCGGAUCAA

Novel37, scaffold_52:447370:447483, dG= -37.30

10 20 30 40

G-- GCAA CG G AU .-GCAAU| UG

CUU CCAACAAG U UAG CAGCUG GGGU U

GAG GGUUGUUC A AUU GUCGAC UCCA U

UUG AG-- AU A GC \ -----^ UC

110 100 90 50

60

UAA-- U U AG

CCAA G CCUC \

GGUU C GGAG U

AUAAA - - CU

1. 70

sRNA (5' to 3' orientation) mapped to this predicted precursor hairpin:AACAAGCGUGUAGAUCAGCUG

Novel38, scaffold_53:1195181:1195275, dG = -51.70

10 20 30 40

UC AC -| GG C - CG CGUG

AGGG UCUUGUCGCAGGA GC UGGCAC UGC AC GCCGC \

UCCC AGAACAGUGUCCU CG ACCGUG ACG UG UGGUG U

AA GA A^ -- A C -- UAUG

90 80 70 60 50

sRNA (5' to 3' orientation) mapped to this predicted precursor hairpin:UUGUCGCAGGAGCGGUGGCACC

Novel39, scaffold_57:106595:106727, dG=- 87.60

10 20 30 40 50 60

CAAAUUAC| C A ACU GG

GGUGCAACUGUGGUACCGUGCUACAGUUGCAU UAGCUGUUG AUGC UAGAUU \

CCACGUUGACACCAUGGCACGGUGUUAACGUA GUCGACAAU UACG AUCUGG U

UUUGAACA^ A C GUU GG

130 120 110 100 90 80 70

sRNA (5' to 3' orientation) mapped to this predicted precursor hairpin: UGCAACUGUGGUACCGUGCUA

Novel40, scaffold_5:715526:715624, dG = -41.10

10 20 30 40 50

G| A GUU UA C UC AUU UG

GAGU AA UGAUGUUGGCA GCUCAAUC GA AAA CUCAG \

CUUA UU ACUAUAACCGU CGAGUUAG CU UUU GAGUC C

-^ C AC- GC U UA CCU GA

90 80 70 60

sRNA (5' to 3' orientation) mapped to this predicted precursor hairpin: UGUUGGCAUAGCUCAAUCCGA

Novel41, scaffold_6:4465828:4465981, dG = -59.00

10 20 30 40 50 60 70

UGA U --- A A U U AACAUU| ------------- - A

UUAG GC AGGUGG GAGUUCC AAGGGAUCGCA UGAUCUGA GAU AAAUUA AUCA CUC A

AAUC CG UCUACC CUUAAGG UUCCCUAGCGU ACUAGAUU UUA UUUAGU UAGU GGG A

UA- U GUA C C - U CUUUC-^ AUAUAUAUUAUUU U C

150 140 130 120 110 100 90 80

sRNA (5' to 3' orientation) mapped to this predicted precursor hairpin:UUCCAAAGGGAUCGCAUUGAUC

Novel42, scaffold_77:492511:492620, dG = -22.90

10 20 30 40 50

GAUACA- GU-- A AU-| U AUU UUAU AAAUA--- CA

CA UGG AAAUA GA CGUAGA CC GGA GCC U

GU ACC UUUGU CU GCAUCU GG CCU CGG G

CCGAAUA AACC G CAU^ C AU- UU-- GUACGACG AU

. 100 90 80 70 60

sRNA (5' to 3' orientation) mapped to this predicted precursor hairpin:UGGAAAAUAAUGAUCGUAGAA

Novel43, scaffold_81:895369:895480, dG= -84.00

10 20 30 40 50

U| CUA AUA A

GCAA UGGUAUCAUACCACAGCUGCACCUAGCUAUUGGAUUCAGCU GUAC U

CGUU ACCAUGGUAUGGUGUCGACGUGGAUCGAUAAUCUAAGUCGA CGUG G

-^ AAC --- U

110 100 90 80 70 60

sRNA (5' to 3' orientation) mapped to this predicted precursor hairpin: UCAUACCACAGCUGCACCUAG

Novel44, scaffold_8:1444329:1444467, dG = -56.50

10 20 30 40

UGCUGC- - GGU A .-A| U

CA AUAUUUGC UGCGGCAUCAUCAAGAUUC CA GC U

GU UAUAAACG ACGUCGUAGUAGUUCUAAG GU CG U

AGCAUCA A ACU G \ -^ A

130 120 110 100 50

60 70

A------- CA G

AGGGAAUU UUGAAUA G

UUCCUUGA AACUUAU C

AAUAAACC A- C

90 80

sRNA (5' to 3' orientation) mapped to this predicted precursor hairpin: GCGGCAUCAUCAAGAUUCACA

Novel45, scaffold_8:3530405:3530512, dG = -47.70

10 20 30 40 50

CUGU G A - AAAUACACG -| U GU

UUGC GGUGUAGCAUCAUCAAGAUUC CA UGC GC AG GAU G

AACG CUACGUCGUAGUAGUUCUAAG GU ACG CG UC CUA A

GAAU A A C AAG------ U^ U AU

100 90 80 70 60

sRNA (5' to 3' orientation) mapped to this predicted precursor hairpin: GUAGCAUCAUCAAGAUUCACA

Novel46, scaffold_9:4701627:4701969, dG = -106.60

10 20 30 40 50

U .-AG U U - - AAA -------------| AG

GUUGG UUGAGGGGAA GG GG CUGGUUCAAGGCU UC AGAGA GUUUCUUA \

CAACC AACUCUCCUU CC UC GACCAGGUUUCGA AG UCUCU CAAAGAAU A

U \ -- - U G U AAA CUCUAAUUAUUAA^ AA

340 120 110 100 90 80 70 60

130 140 150 160 170 180 190

.-AAUUAAAC - A UA CUUUUCAUUUUGUU U UC UGA UC

ACCUA UCU UUAU AAUGGCUC AAUUU UUUGCU UGAU UC \

UGGAU AGG AGUG UUAUUGAG UUAAA GAACGG GUUA AG A

\ -------- U - -- UUAUUUUCUU---- C GU UG- AU

240 230 220 210 200

250 260 270 280 290

UG- A UU CU UC- GG AAGA A

AG GGAAUG GUCUGG CAAGG AU AGG AUA A

UC CCUUAC CGGACC GUUCU UA UCC UAU U

AAA A UU AG UAU AA CGAG A

330 320 310 300

sRNA (5' to 3' orientation) mapped to this predicted precursor hairpin:GGAAUGUUGUCUGGCUCAAGG

Novel47, scaffold_11:564547:564628, dG = -38.00

10 20 30 40

C| G A C C C CA

CGGGAAA CUUUAAAAUC CA GG UUUAAAGGC UA G

GCCCUUU GAAAUUUUGG GU CC AAAUUUCCG AU U

C^ G C A A A UU

80 70 60 50

sRNA (5' to 3' orientation) mapped to this predicted precursor hairpin: UUUAAAAUCACACGGCUUUAA

Novel48, scaffold_11:2291314:2291391, dG = -18.70

10 20 30

UAAUAAUCC A--| G GGA C U

GCA UC AUGGAUU UAUGGAU UAC U

UGU AG UGCCUAA AUGCCUA AUG U

UAAAAAAAA CUA^ - AA- A U

70 60 50 40

sRNA (5' to 3' orientation) mapped to this predicted precursor hairpin:CAAUCGAUGGAUUGGAUAUGGA

Novel49, scaffold_164:51980:52061, dG = -22.20

10 20 30 40

UUGAA C-| A C CA G- A

ACUA GACCCA AUCA ACAAU AAGUUU UGGG \

UGAU CUGGGU UAGU UGUUA UUUAAA ACUC C

UGGUG AA^ A - -- GA U

80 70 60 50

sRNA (5' to 3' orientation) mapped to this predicted precursor hairpin:ACCCAAAUCACACAAUCAAAGUUU

Novel50, scaffold_20:1575648:1575778, dG = -58.90

10 20 30 40 50 60 70

U A A A C A CAUA| A AACACUCCCACCACA A A

GGUAG AGAA UCCC CAGGGGCGAC UGAGA CACA AAC CA CC AAUGU U

CCAUC UCUU AGGG GUCCCCGCUG ACUCU GUGU UUG GU GG UUACG A

- - C C U A ACG-^ C AAC------------ A G

130 120 110 100 90 80

sRNA (5' to 3' orientation) mapped to this predicted precursor hairpin: CCACAGGGGCGACCUGAGAAC

Novel51, scaffold_3:4619018:4619126, dG = -42.80

10 20 30 40 50

-| U U C CA A CACCACUU UCCU GGU

AGC CC UG UGGAGAAG GGGC CGUGCAAA UGGC UUC \

UUG GG AC ACCUCUUC CUCG GCACGUUU ACCG AAG U

C^ - U A CC C CCUAAAUC UU-- ACU

100 90 80 70 60

sRNA (5' to 3' orientation) mapped to this predicted precursor hairpin:UGGAGAAGCAGGGCACGUGCAAA

Novel52, scaffold_4:4045480:4045585, dG = -40.60

10 20 30 40 50

G C C A C A AA-- CAU--| AG

UUUGUAU UU CCACAGCUUU UUGAAC GCA CA UGC GCCA U

AAACAUA AA GGUGUCGAAA AACUUG CGU GU ACG CGGU C

G A A G A A AGAA UACUC^ GU

100 90 80 70 60

sRNA (5' to 3' orientation) mapped to this predicted precursor hairpin:UCCCACAGCUUUAUUGAACCGC

Novel53, scaffold_4:4081925:4082032, dG = -38.90

10 20 30 40 50

AAAAAACGA C A C A - GA- -| AU

AAG GAUAUUGGUG GGUUCAAUC G AGA CG UUUAC AC U

UUC CUAUAACCGC CCGAGUUAG C UCU GC AAAUG UG G

AAUAUUUAA A G A - A AGA U^ AA

100 90 80 70 60

sRNA (5' to 3' orientation) mapped to this predicted precursor hairpin:CGAUAUUGGUGAGGUUCAAUC

Novel54, scaffold_4:4121669:4121798, dG = -60.70

10 20 30 40 50

UGUAAAAA----- AAGA A - A .-G| UU

GGGU GG GGUGACAGA AGAGAGUGAGCAC CAU GUA U

CCCA CC CCACUGUCU UCUCUCACUCGUG GUA CGU U

AACCCCCCGCCCC A--- - A C \ -^ UC

. 120 110 100 90

sRNA (5' to 3' orientation) mapped to this predicted precursor hairpin:GUGACAGAAGAGAGUGAGCAC

Novel55, scaffold_76:1019946:1020071, dG = -23.60

10 20 30

- C -- A .-A UU

UUAGGGAU GAUA GUCUGAAGU GAAG UAGUU \

AAUUUUUA UUGU UAGACUUCA UUUU AUCGG A

A - GC G \ - GU

120 110 100 40

50

.-AAAA| G

AUGUA U

UACGU G

\ ----^ A

60

70 80

AAAAAAAAUAAAAAGAAUACA GU

UUGG \

GAUC U

AC------------------- AG

90

sRNA (5' to 3' orientation) mapped to this predicted precursor hairpin:AUAGUCUGAAGUAGAAGAUAGUU

Novel56, scaffold_80:25066:25172, dG = -48.60

10 20 30 40

---------| U CUGU - U - A UGG

GA GAUG UGACGGAAGA UAGAGAGCACAGA GA UGA AUGCG \

CU CUAC ACUGUCUUCU AUCUCUCGUGUUU CU ACU UACGU A

CCUCUCCGA^ U U--- U C C C UCG

100 90 80 70 60

sRNA (5' to 3' orientation) mapped to this predicted precursor hairpin:UUGACGGAAGAUAGAGAGCAC

Novel57, scaffold_95:696868:696981, dG = -44.90

10 20 30 40 50

AAA U - U U C --- U --| GG GAGUA

UGUU G UUAGCCAAGGA GAC UGCCUG GU CA GCA AGAG UUUC \

ACAG C AAUCGGUUCCU CUG ACGGAC CG GU UGU UCUC AAAG G

CCG U G - - - UUG U CA^ GA AUAUC

110 100 90 80 70 60

sRNA (5' to 3' orientation) mapped to this predicted precursor hairpin:UAGCCAAGGAUGACUUGCCU

Novel58, scaffold_95:743113:743226, dG = -51.20

10 20 30 40 50

- A U - U U AU- U UA U -------| A

GCU GUU GG UAGCCAAGGA GAC UGCCUG CUC CC AG GGU UUCAA A

CGA CAG UC AUCGGUUCCU CUG ACGGAC GGG GG UC CCA AAGUU C

C - U A - - CAU U GC - AAUUUAU^ G

110 100 90 80 70 60

sRNA (5' to 3' orientation) mapped to this predicted precursor hairpin:UAGCCAAGGAUGACUUGCCUGA

Novel59, scaffold_9:4621734:4621925, dG = -57.00

10 20 30 40 50 60

AAA AGC UU AAUUAAAC .-UC| U U UUC

GAU UUUGGACCAGGC CAUUCCUCUCAA ACCUA UAU AU AAAUGG \

CUA GAACUUGGUCUG GUAAGGAGAGUU UGGAU AUA UG UUUACU U

GUA --- UU -------- \ --^ U U UUU

190 180 170 160 80 70

90 100 110 120

UGUUUCG UG UGAU U---- UA AA

CUUU AU CUCA ACAA UUGC A

GAGA UA GAGU UGUU AACG G

UGG---- GU UU-- UAUUU UA AA

150 140 130

sRNA (5' to 3' orientation) mapped to this predicted precursor hairpin:UUGGACCAGGCUUCAUUCCUC

Novel60, scaffold_103:171417:171509, dG = -29.00

10

A UUU .-A| UG

CAGC GUA GAAG \

GUCG CGU CUUU C

- UUU \ -^ UC

90 20

30 40 50

CUUUUG C CGUCGAC

GCUUUCUC CUCUAACU U

CGAAAGAG GAGAUUGA U

UG---- A ACUUCCU

80 70 60

sRNA (5' to 3' orientation) mapped to this predicted precursor hairpin:GCUUUCUCCCUCUAACUCGUCG

Novel61, scaffold_12:3883367:3883573, dG = -86.40

10 20 30 40 50 60 70 80 90 100

GUU UUU --- --| UU CU U AA AG- G AC UC AA A AAAGG GA

GAU GAUUA AGC UGGGAGCU CUUCGGUCCA UAUGGG GGC UAGGAUUUAAUU CU CUG UCAUUCA CA UACUG GU GUC A

CUA CUAAU UCG ACCCUCGA GAAGUCAGGU GUGUCC UCG AUUCUAAGUUAA GA GGC AGUAAGU GU AUGAC CA UAG A

C-- UU- CGC UU^ GG UC U A- ACA G GU GA AA C ----- GU

200 190 180 170 160 150 140 130 120 110

sRNA (5' to 3' orientation) mapped to this predicted precursor hairpin:GAGCUUUCUUCGGUCCACUU

Novel62, scaffold_13:4483423:4483508, dG = -24.00

10 20 30 40

- ---- CA-- UG --| GGGUA G

CGGCA AAU CUACU GUAG GAUACUU GAUAAA A

GCUGU UUA GGUGA CAUC UUAUGAG UUGUUU A

G CCCA CUUC GU UU^ ----- A

80 70 60 50

sRNA (5' to 3' orientation) mapped to this predicted precursor hairpin:CUACUUGGUAGGAUACUUGGG

Novel63, scaffold_15:395989:396110, dG = -25.80

10 20 30 40 50

UGUUAAA - G- - AGAA .-AUGUCAAGAAAAAGUG| AG

UGCUACA ACU UG GCA AUGGC UUGG \

ACGAUGU UGG AC CGU UACUG GACC U

UCUAUGA G GA U AAAC \ ----------------^ AA

120 110 100 90 60

70

AAUAGAAAAGA AA

GGGU \

UCCA A

AG--------- AG

80

sRNA (5' to 3' orientation) mapped to this predicted precursor hairpin:UACAACUGUGGCAAGAAAUGGCA

Novel64, scaffold_17:2390469:2390560, dG = -63.90

10 20 30 40

AGG| A C U GGGU

GAC GCCCACG GGCCA CUCUCAUUGAGAGAGCUGUG C

CUG CGGGUGU CCGGU GAGAGUAACUCUCUUGACAC A

UUA^ A A C GAUA

90 80 70 60 50

sRNA (5' to 3' orientation) mapped to this predicted precursor hairpin:CACGCGGCCAUCUCUCAUUGAG

Novel65, scaffold_26:118376:118514, dG = -42.20

10 20 30 40 50

.-U G UG- C C- -- C UU- CU-- ---| AUA

UC CG GUCGAG AGAU CUGA CA GC GGCUU GGUUUCA CC A

AG GU UAGCUU UUUA GAUU GU CG CCGAG UCAGAGU GG A

\ - G UCG - AA CC A UUU UAGU UUC^ ACA

110 100 90 80 70 60

120

G - UUU

UCUGCUCG CC \

AGACGAGC GG U

- U UUC

130

sRNA (5' to 3' orientation) mapped to this predicted precursor hairpin: CGAGCAGAUCCUGACACGCUU

Novel66, scaffold_37:247375:247555, dG = -37.80

10 20 30 40 50 60 70

CU AGAUUAUAAACGAA GA--- UU CG UACA ACG- .-AUAUU| GA

AGAGG UCCUAU GAG CG AUUU AGGAAAAA AAG UCAU C

UUUCC AGGAUG CUC GC UAAA UCCUUUUU UUC AGUA A

AU -------------- GGUUG UU AU UAA- CAAA \ -----^ AA

. 170 160 150 140

80 90 100

UUU---- UU CUGUUG

UGAGAAAGGAG CU \

ACUUUUUCUUC GA G

GAAUCUU UU AAAAUU

130 120 110

sRNA (5' to 3' orientation) mapped to this predicted precursor hairpin: UCCUAUGAGAGUUCGCGAUUUUA

Novel67, scaffold_39:1309710:1309970, dG = -38.70

10 20 30 40 50 60 70 80

UCUU GA GA A- A .-UU UAGCU- AG .-GCUUCUAAAAGUC| U UUA

AAUCU GCAGAU UG UACAA AACA AUUCA GAUGA CCGUGAU GAGC UU A

UUAGG UGUUUG AC AUGUU UUGU UGAGU CUAUU GGUACUG CUCG AG A

UCCU -- AG GA G \ -- UUUUCU CA \ -------------^ U UCA

. 250 240 140 130 90

100

C---------- UUCA

GUAA \

CGUU U

UUCUUUCUACC CUUU

120 110

150 160

.-UAUUAUUACUAA CAU

GUUC \

CAAG C

\ ------------ CUG

170

180 190 200

AC-- A UCU UCCUAC

UUUUU GUUUUU UUU C

AAAAA UAAAAA AAA A

UAAA A UUU UCUUAU

230 220 210

sRNA (5' to 3' orientation) mapped to this predicted precursor hairpin:AGCAGAUGAUGAUACAAAAACA

Novel68, scaffold_3:1219690:1219806, dG = -25.00

10 20 30 40 50 60

UUC| UGUCU GUGAU U AAU A G AGACU G U

GAAU ACUGCU GA UUAUGAAUG UC CAU UC UCA AGAUUUUU U

CUUA UGACGA CU AAUGCUUAU AG GUA AG AGU UCUAAAAA G

UUU^ UAU-- GU--- U --- - - ----- A A

110 100 90 80 70

sRNA (5' to 3' orientation) mapped to this predicted precursor hairpin:UGCUGUGAUGAUUUAUGAAUGAAU

Novel69, scaffold_40:130452:130527, dG = -18.00

10 20 30

AU| AA G UA C UUUU

CAAUAAUUU AGA GG AAAUAGCUAA AA A

GUUAUUAAA UCU CC UUUAUUGAUU UU A

UU^ CA G -- U UUUU

70 60 50 40

sRNA (5' to 3' orientation) mapped to this predicted precursor hairpin:UAAAGAGGGUAAAAUAGCUAACA

Novel70, scaffold_43:656273:656366, dG = -33.60

10 20 30

GU- | C U A

GUGUGUGA--UCAACU GGCGAUGCAUUAA UAA \

UAUACACU AGUUGA CCGCUACGUAAUU AUU G

UCU \ ^ A C U

90 60 50 40

AU---------- G

AU A

UG A

AUAUUAAAGUUC A

1. 70

sRNA (5' to 3' orientation) mapped to this predicted precursor hairpin:UCAACUCGGCGAUGCAUUAAUUA

Novel71, scaffold_44:542896:542995, dG = -53.50

10 20 30 40 50

AUAUA| C C U A C A

UAGUAUGC UGGCUCC UGUAUGCU UUUGC AAG CCAUCAG A

AUCGUACG ACCGAGG GCGUGCGA AGGCG UUC GGUGGUU G

UUUAA^ U A U G U A

. 90 80 70 60

sRNA (5' to 3' orientation) mapped to this predicted precursor hairpin:UGCCUGGCUCCCUGUAUGCUU

Novel72, scaffold_46:1225861:1225959, dG = -53.90

10 20 30 40 50

U| C C UUA G

ACAUUGAAUGUAUCAUACACAA CAAUAAGAACCA AUAA UGUGG \

UGUAACUUACAUGGUAUGUGUU GUUAUUCUUGGU UAUU AUACC U

-^ A A UA- C

90 80 70 60

sRNA (5' to 3' orientation) mapped to this predicted precursor hairpin:UCAUACACAACCAAUAAGAACC

Novel73, scaffold_56:198262:198499, dG = -50.60

10 20 30 40 50 60 70

- G A G - - C CA .-UAUGUUCCA U .-ACCA| AA

GA UUGUUC AAUUC AUUGG UAGAU GGGUA AGA AUUGAA ACCAGAAAAG AAAA UUUC C

CU AGCAAG UUGAG UAACU AUUUA UCCAU UUU UAAUUU UGGUUUUUUU UUUU AAAG A

A A G - A G C A- \ --------- - \ ----^ AA

230 220 210 200 130 120 80

90 100

AA- -- UGA

AUUCU UCCUU \

UAGGA AGGGA G

CAA AA UCU

110

140 150 160

--- A- UG GAAAAC CUA

GAGGG AU UU GAUAAGA \

UUCCU UA AG UUGUUCU U

UUU CG GU AAGCGU AUA

190 180 170

sRNA (5' to 3' orientation) mapped to this predicted precursor hairpin:AAUUCGAUUGGUAGAUGGGUA

Novel74, scaffold_5:7200012:7200132, dG = -22.80

10 20 30 40 50

UUCUUUUGUU UCA GGC AG AAA .-UA| AA

UUGGCAAU GA UC AGUAU ACAGCUU UC U

AACCGUUA CU AG UUAUA UGUCGAA AG C

CUAUCAAAGU CUG --- CU --- \ --^ AA

. 110 100 90

60

AUU------ A

UUCUG \

AAGAC A

UAUACGGUC G

1. 70

sRNA (5' to 3' orientation) mapped to this predicted precursor hairpin: GGCAAUUCAGAGGCUCAGAGUAU

Novel75, scaffold_8:587750:587857, dG = -64.00

10 20 30 40 50

-| ACAG U UG UG

CUAUGUUACGUUGAACGUAAUAUACACACACUC CAACAAUA UAA G G

GAUACAAUGCAACUUGCAUUGUAUGUGUGUGAG GUUGUUAU AUU U G

G^ A--- C GU CG

100 90 80 70 60

sRNA (5' to 3' orientation) mapped to this predicted precursor hairpin:UUGAACGUAAUAUACACACAC
